# DDX5 (p68) and UbE2T as emerging superior cancer therapeutic targets: dual molecular glue target degradation by FL118 for conquering difficult-to-treat cancers

**DOI:** 10.1186/s13046-026-03733-3

**Published:** 2026-05-18

**Authors:** Fengzhi Li, Xiang Ling, Sayan Chakraborty, Dean G. Tang, Karen McLean, Wenjie Wu, Norbert Sule, Deepak Vadehra, Christos Fountzilas, Ajay Gupta, Clare Twist, Joyce Ohm, Gurkamal Chatta, Scott I. Abrams

**Affiliations:** 1 Department of Pharmacology & Therapeutics, Roswell Park Comprehensive Cancer Center, Buffalo, NY 14263 USA; 2 Developmental Therapeutics Program, Roswell Park Comprehensive Cancer Center, Buffalo, NY 14263 USA; 3 Pancreatic Cancer Research Center, Roswell Park Comprehensive Cancer Center, Buffalo, NY 14263 USA; 4 Canget BioTekpharma LLC, Buffalo, NY 14203 USA; 5 Department of Gynecology, Roswell Park Comprehensive Cancer Center, Buffalo, NY 14263 USA; 6 Department of Pathology, Roswell Park Comprehensive Cancer Center, Buffalo, NY 14263 USA; 7 Department of Medicine, Roswell Park Comprehensive Cancer Center, Buffalo, NY 14263 USA; 8 Department of Pediatrics, Roswell Park Comprehensive Cancer Center, Buffalo, NY 14263 USA; 9 Department of Cancer Genetics & Genomics, Roswell Park Comprehensive Cancer Center, Buffalo, NY 14263 USA; 10 Department of Immunology, Roswell Park Comprehensive Cancer Center, Buffalo, NY 14263 USA; 11 Tumor Immunology & Immunotherapy Program, Roswell Park Comprehensive Cancer Center, Buffalo, NY 14263 USA

**Keywords:** DDX5 (p68), UbE2T, FL118, Dual molecular glue degrader, Targeted protein degradation, FL118 analogs, Cancer stem cells, DNA damage repair, Difficult-to-treat cancers, Targeted cancer therapeutics

## Abstract

Growing evidence has revealed that DEAD-box RNA helicase 5 (DDX5, also known as p68) and ubiquitin-conjugating enzyme E2T (UbE2T) are two emerging and highly promising cancer therapeutic targets. This article provides the first comprehensive review of the physical and functional relationship between these two cancer targets, further refining their therapeutic potentials in solid cancers. In addition, the consequences of simultaneously degrading DDX5 and UbE2T proteins by the small-molecule dual molecular glue degrader FL118 in difficult-to-treat advanced cancers are presented.

Specifically, this article reviews: (1) the roles of DDX5 and UbE2T in diverse cancer DNA repair pathways; (2) the physical binding relationship and potential functional roles of DDX5 in topoisomerase regulation; (3) the involvement of DDX5 in EZH2- and NANOG-associated prostate cancer stem cell (PCSC)–driven neuroendocrine prostate cancer (NEPC), castration-resistant prostate cancer (CRPC), and metastatic CRPC (mCRPC); (4) the contributions of DDX5 and UbE2T to inflammatory and immune regulation within the tumor microenvironment (TME); (5) FL118 as a small-molecule dual molecular glue degrader selectively targeting both DDX5 and UbE2T; (6) the high efficacy of FL118 against multiple difficult-to-treat advanced and metastatic cancers, including advanced colorectal cancer (CRC), pancreatic ductal adenocarcinoma (PDAC), osteosarcoma, Ewing sarcoma, ovarian cancer, and glioma/glioblastoma; (7) the resistance of ABCG2-expressing cancer cells to common anticancer agents but not to FL118; (8) the favorable pharmacokinetic and toxicology profiles of FL118 in mice, rats, and dogs; (9) the distinct functions of DDX5 in normal tissues, cells, and organs versus cancer; and (10) FL118 as a drug platform enabling the development of novel analogs and derivatives.

Based on this review, we conclude that DDX5 and UbE2T represent superior anticancer therapeutic targets, and that the high efficacy of FL118 against multiple difficult-to-treat cancers is attributable to its function as a bona fide small-molecule dual molecular glue degrader that physically targets and degrades both DDX5 and UbE2T. Strikingly, this activity is observed regardless of the expression status of ABC transporter proteins, ABCG2/BCRP, ABCB1/Pgp/MDR1, and/or ABCC1/MRP1 in cancer cells.

**Supplementary Information:**

The online version contains supplementary material available at 10.1186/s13046-026-03733-3.

## Background

Recent studies indicate that DEAD-box RNA helicase 5 (DDX5) plays critical roles in cancer DNA repair, immune suppression, oncogenic metabolic rewiring, viral infection promotion, and detrimental modulation of the human microbiota [1]. These observations continue to expand and gain support. For example, DDX5 has been implicated in immune suppression [2], in aurora-A kinase interacting protein 1 (AURKAIP1)–mediated triple-negative breast cancer progression via the DDX5/β-catenin axis [3], in esophageal squamous cell carcinoma progression [4], and in the prevention of ferroptosis in glioma stem cells by maintaining glutathione peroxidase 4 (GPX4)–mediated inhibition of mitochondrial reactive oxygen species (ROS)–induced lipid peroxidation [5].

Studies of the ubiquitin-conjugating enzyme E2T (UbE2T) have likewise revealed its important roles in tumorigenesis and cancer therapeutics [6, 7]. Additional background on RNA helicases (RHs), their cellular functions, and their links to human diseases can be found in recent reviews and their cited references [1, 8].

In this article, we review: (i) the roles of DDX5 and UbE2T in diverse cancer DNA repair pathways; (ii) the physical binding relationship and potential functional roles of DDX5 in topoisomerase regulation; (iii) the involvement of DDX5 in EZH2- and NANOG-associated prostate cancer stem cell (PCSC)-driven neuroendocrine prostate cancer (NEPC), castration-resistant prostate cancer (CRPC), and metastatic CRPC (mCRPC); (iv) the roles of DDX5 and UbE2T in inflammatory and immune regulation within the tumor microenvironment (TME); (v) FL118 as a small-molecule dual molecular glue degrader targeting both DDX5 and UbE2T; (vi) FL118 shows high efficacy against difficult-to-treat advanced and metastatic cancers, including advanced CRC, PDAC, osteosarcoma, Ewing sarcoma, ovarian cancer, and glioma/glioblastoma multiforme (GBM); and (vii) the resistance of ABCG2-expressing cancer cells to common anticancer agents but not to FL118; (viii) the favorable pharmacokinetic and toxicology profiles of FL118 in mice, rats, and dogs; (ix) the distinct functions of DDX5 in normal tissues, cells, and organs versus cancer which could mitigate off-target impacts; and (x) FL118 as a drug platform enabling the development of next generation novel analogs and derivatives.

### DDX5 and UbE2T in promoting cancer DNA repair

Existing studies indicate that DDX5 plays a central, cross-pathway role in abnormal and damaged DNA repair, thereby contributing to cancer cell survival, disease progression, and treatment resistance. Based on the known DDX5-involved protein-protein interactions (PPIs), DDX5 is involved in nearly all major DNA damage response (DDR) pathways in cancer, including homologous recombination (HR) repair (HRR), non-homologous end joining (NHEJ), base excision repair (BER), nucleotide excision repair (NER), and the Fanconi anemia (FA) DNA repair pathway. In contrast, UbE2T primarily functions within the FA DNA repair pathway. Thus, these two proteins exhibit overlapping yet distinct roles in cancer-associated DNA repair processes and pathway crosstalk. Below, we review key observations that support our overall summary of the roles of DDX5 and UbE2T in cancer DNA repair. However, in many instances, particularly for DDX5, the precise mechanistic roles remain incompletely defined and warrant further investigation.

### DDX5 in HRR and NHEJ

HRR and NHEJ are two major mechanistic pathways in DDR [9].

### Role of DDX5 in BRCA1 and BRCA2

**Fig. 1:**
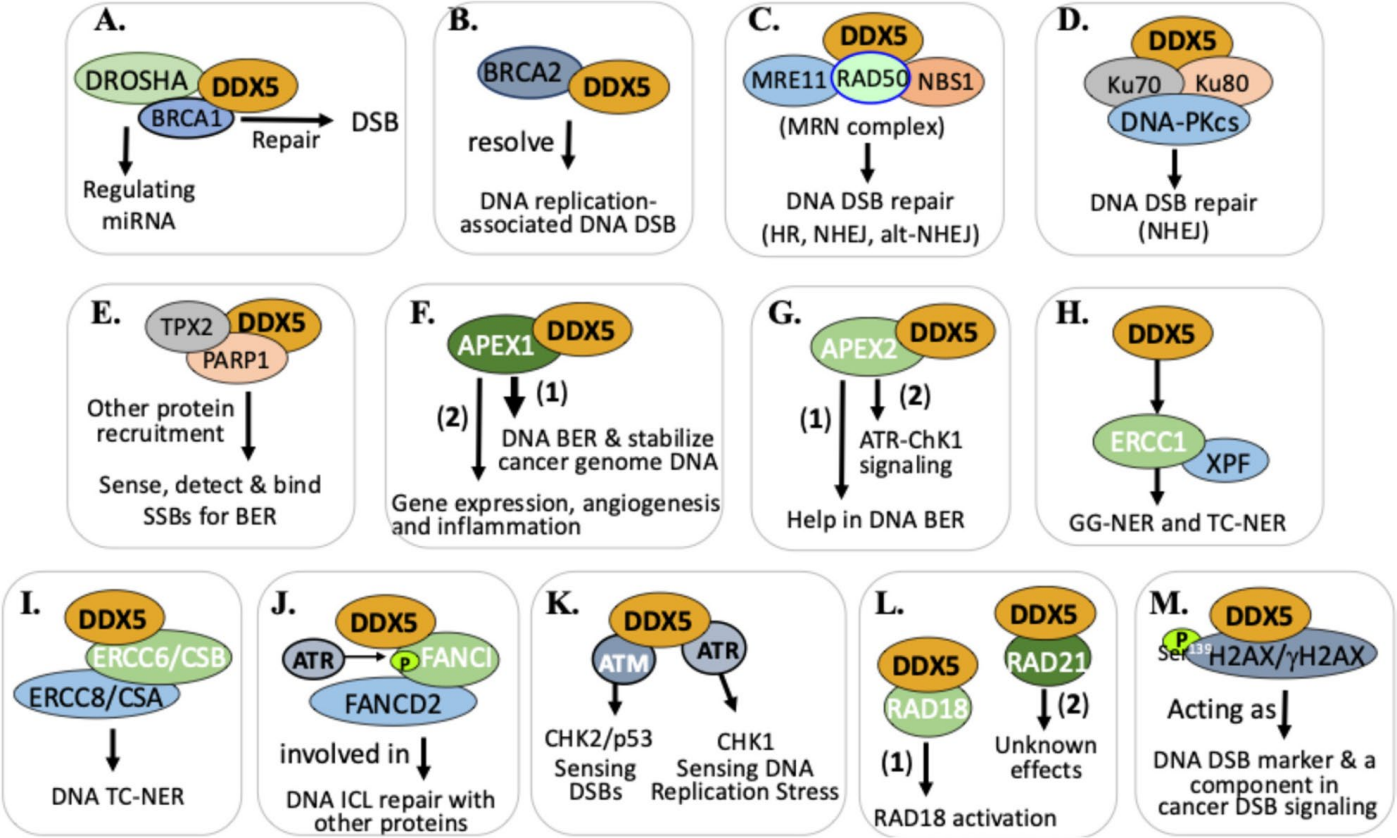
Potential involvement of DDX5 (p68) in multiple cancer DNA repair pathways: (**A**) Potential involvement of DDX5 in BRCA1-mediated DNA double-strand break (DSB) repair. **B** Potential involvement of DDX5 in BRCA2-mediated DNA DSB repair. **C** Potential involvement of DDX5 in RAD50/MRN complex–mediated DNA DSB repair. **D** Potential involvement of DDX5 in Ku70/80/DNA-PKcs complex–mediated DNA DSB repair. **E** Potential involvement of DDX5 in PARP1-mediated DNA base excision repair (BER). **F** Potential involvement of DDX5 in APEX1-mediated DNA BER. **G** Potential involvement of DDX5 in APEX2-mediated DNA BER. **H** Potential involvement of DDX5 in ERCC1-mediated DNA nucleotide excision repair (NER). **I** Potential involvement of DDX5 in ERCC6/CSB-mediated DNA NER. **J** Potential involvement of DDX5 in the FANCI-mediated Fanconi anemia (FA) pathway. **K** Potential involvement of DDX5 in ATM- and ATR-mediated DNA damage response (DDR) signaling. **L** Potential involvement of DDX5 in RAD18- and RAD21-mediated DDR signaling. **M** Potential involvement of DDX5 in γH2AX-mediated DDR signaling

The tumor suppressor breast cancer 1 (BRCA1) plays a critical role in DNA double-strand break (DSB) repair [10–12]. However, the involvement of DDX5 in BRCA1-mediated DSB repair remains relatively underexplored. Using HeLa, HEK293, and the osteosarcoma cell line MG63 as model systems, it was demonstrated that BRCA1 directly associates with DROSHA and DDX5 within the DROSHA microprocessor complex (Fig. 1A), which regulates the maturation of multiple microRNAs, including let-7a-1, miR-16-1, miR-145, and miR-34a [13]. Importantly, DDX5, together with its paralog DDX17, functions as a molecular bridge between DROSHA and other proteins such as BRCA1 within this complex in cancer cells [14]. Subsequent studies showed that DDX5 binds to DROSHA in breast cancer cells [15], whereas DROSHA reciprocally binds to DDX5 in rhabdomyosarcoma cells [16]. These findings extend the role of DDX5 beyond direct participation in DNA DSB repair to include regulation of miRNA biogenesis through interaction with the DROSHA complex (Fig. 1A).

Similarly, DDX5 has been shown to physically interact with BRCA2 in U2OS osteosarcoma cells (Fig. 1B). BRCA2 facilitates the retention of DDX5 at DNA damage sites, and the DDX5-BRCA2 complex promotes HR-mediated repair of DNA DSBs, resolving replication-associated breaks and thereby preventing cancer cell death [1, 17].

### Role of DDX5 in RAD50

RAD50 is a core component of the MRE11-RAD50-NBS1 (MRN) complex, which senses DNA DSBs and initiates damage signaling and repair through both HR and NHEJ pathways [18]. Defective RAD50 impairs DSB repair, increasing mutation burden and cancer risk. Depending on cellular context, RAD50 may suppress tumorigenesis by maintaining genome integrity or promote tumor survival by facilitating DSB repair in cancer cells; thus, its prognostic significance is cancer-type dependent [18]. Nevertheless, aberrant RAD50 expression has been linked to poor prognosis and therapeutic resistance in multiple cancers [18].

Using an inducible knockout mouse model, Legrand et al. demonstrated that DDX5 deletion in spermatogonia led to significant downregulation of RAD50, identifying DDX5 as an important regulator of RAD50 mRNA splicing [19]. These findings suggest that DDX5 may functionally and/or physically interact with RAD50 (Fig. 1C). Although the precise role of DDX5 in MRN-mediated DNA repair in cancer requires further investigation, existing evidence supports RAD50 as a key mediator through which DDX5 contributes to HR and NHEJ, consistent with the model illustrated in Fig. 1D.

### Role of DDX5 in Ku70/80

Consistent with earlier observations that Ku70 binds DDX5 in HEK293 cells [20], Le et al. recently confirmed that DDX5 interacts with the Ku70/80 heterodimer in prostate cancer cells [21]. Ku70/80 (XRCC6/5) plays a pivotal role in NHEJ-mediated DSB repair, a pathway critically implicated in cancer genomic stability, treatment resistance, and disease progression. The Ku70/80 complex binds tightly to DSB ends, protecting them from degradation, aligning broken ends, and facilitating end processing and ligation. It also recruits the DNA-dependent protein kinase catalytic subunit (DNA-PKcs), forming the DNA-PK holoenzyme that initiates NHEJ repair [22, 23] (Fig. 1D).

Taken together, the ability of DDX5 to bind both Ku70/80 and RAD50 suggests that DDX5 may coordinate classical NHEJ (c-NHEJ), primarily mediated by Ku70/80, with HR and alternative NHEJ (alt-NHEJ) pathways mediated by the MRN complex. Accordingly, degradation or inhibition of DDX5 could represent an efficient strategy to broadly suppress cancer DNA repair and induce tumor cell death, supporting the concept that DDX5 functions as a master upstream regulator of cancer DNA repair.

### DDX5 in BER

Base excision repair (BER) corrects small base lesions caused by oxidation, alkylation, or deamination.

### Role of DDX5 in PARP1

Mosler et al. employed PARP1 proximity proteomics at stressed replication forks to identify potential PARP1 regulators and identified DDX5 and TPX2 among the PARP1 proximal proteins [24]. While TPX2 was characterized as a direct PARP1-binding regulator of PARP1 auto-ADP-ribosylation activity, interactome analyses revealed that DDX5 physically and functionally interacts with TPX2 [24] (Fig. 1E).

PARP1 is frequently overexpressed in many cancers [25] and promotes tumor cell survival by sensing and binding DNA single-strand breaks (SSBs) induced by oxidative stress, alkylation, or spontaneous base loss [26, 27]. Upon binding damaged DNA, PARP1 synthesizes poly(ADP-ribose) (PAR) chains using NAD⁺, leading to chromatin relaxation and recruitment of BER proteins. Subsequent auto-PARylation of PARP1 reduces its DNA affinity, allowing repair completion [28–30] (Fig. 1E).

Cancers with defective HR, such as those harboring BRCA1/2 mutations, rely heavily on PARP1-mediated BER for survival. PARP inhibitors (PARPi), including olaparib, niraparib, rucaparib, and talazoparib, exploit this vulnerability to induce synthetic lethality [31]. Given the central role of DDX5 in cancer DNA repair [1], its interaction with PARP1-associated networks is highly significant.

Additionally, PARP1 has been shown to regulate transcription of the immunosuppressive checkpoint CD24 through ADP-ribosylation of DDX5 in pancreatic cancer [1, 32]. Together, these findings position DDX5 as a critical connector linking PARP1-controlled BER with broader DDR network crosstalk (Fig. 1A-D).

### Role of DDX5 in APEX1and APEX2

Antoniali et al. demonstrated that APEX1 directly binds DDX5 [33]. APEX1 is the major apurinic/apyrimidinic (AP) endonuclease responsible for cleaving DNA at the AP site, generating a 3′-OH terminus required for repair synthesis [34]. APEX1 is frequently overexpressed in cancers, where high expression correlates with chemoresistance and poor prognosis. Beyond its BER function in cancer genome stabilization (Fig. 1F1), APEX1 also acts as a redox regulator of transcription factors such as NF-κB, HIF-1α, and p53, influencing gene expression, angiogenesis, inflammation, and metastasis [34, 35] (Fig. 1F2).

DDX5 was also recently reported to interact with APEX2 in 293T cells based on MS/MS analysis of APEX2 pull-down proteins [36]. Although APEX2 has weaker endonuclease activity than APEX1, it participates in AP-site processing and 3′-DNA end repairs under oxidative stress (Fig. 1G1). Moreover, APEX2 facilitates ATR-CHK1 signaling (Fig. 1G2) and exhibits synthetic lethality with BRCA1/2, but not APEX1, in pancreatic cancer cells [37, 38].

Collectively, these findings further support DDX5 as an especially attractive therapeutic target due to its involvement in APEX1- and APEX2-mediated BER and broader cancer-associated functions (Fig. 1F–G).

### DDX5 in NER

Nucleotide excision repair (NER) removes bulky, helix-distorting DNA lesions through coordinated lesion recognition, excision, repair synthesis, and ligation [39, 40].

### Role of DDX5 in ERCC1 and ERCC6

Although direct interaction between DDX5 and ERCC1 has not yet been demonstrated, DDX5 is a validated direct target of FL118 and undergoes degradation upon FL118 treatment [41]. Consistent with the role of DDX5 in cancer DNA repair [1], FL118 inhibits ERCC1 expression in lung cancer stem cells [42]. ERCC1 forms a heterodimer with XPF (ERCC4) to catalyze the 5′ incision during both global genome NER (GG-NER) and transcription-coupled NER (TC-NER) [40] (Fig. 1H). High ERCC1 expression correlates with resistance to platinum-based chemotherapy and serves as a predictive biomarker for therapeutic response [43, 44]. Targeting ERCC1–XPF enhances chemosensitivity [45], highlighting the need to elucidate the mechanistic role of DDX5 in ERCC1-mediated NER.

FL118 also effectively inhibits ERCC6 expression and induces γ-H2AX in pancreatic cancer cells [46]. Tandem affinity purification and co-immunoprecipitation studies further identified DDX5 as an ERCC6 (CSB)-interacting protein [47]. While ERCC6 is dispensable for GG-NER, it is essential for TC-NER [39, 40]. Upon transcription-blocking DNA damage, ERCC6 cooperates with ERCC8 (CSA) and others to remodel chromatin and displace stalled RNA polymerase II, enabling repair (Fig. 1I). ERCC6 has emerged as a prognostic marker and therapeutic target across multiple cancers [48]. These observations further support DDX5 as a master regulator of cancer DNA repair.

### DDX5 in FA DNA Repair

The Fanconi anemia (FA) pathway repairs DNA interstrand crosslinks (ICLs) and preserves genome stability by stabilizing replication forks [49, 50].

### Role of DDX5 in FANCI

Huang et al. recently reported that DDX5 binds FANCI, although the functional consequences remain unclear [51]. FANCI forms a heterodimer with FANCD2 (ID2 complex), which is monoubiquitinated by the FA core complex and recruited to ICL sites to coordinate downstream repair involving BRCA1, BRCA2, and RAD51 [49, 50]. FANCI phosphorylation by ATR further regulates repair timing and efficiency [49] (Fig. 1J). FANCI is frequently upregulated and amplified in cervical cancer and correlates with poor prognosis [52]. These findings warrant further investigation of DDX5 in FANCI-mediated FA repair.

### DDX5 in DDR pathway crosstalk

We have reviewed the potential DDX5-interacting protein factors involved in different DDR pathways (HRR, NHEJ, BER, NER, and the FA pathway) in cancer (Fig. 1A–J). However, the DDR constitutes a complex and often dysregulated network characterized by extensive crosstalk among pathways, which collectively promotes tumorigenesis, treatment resistance, and potential synthetic lethality–based vulnerabilities. In this regard, DDX5 may contribute to crosstalk among these DDR pathways and potentially others. Here, we provide additional examples to facilitate further investigation into the intriguing mechanisms of action (MOA) of DDX5.

#### Example 1

ATM plays key roles in sensing DNA double-strand breaks (DSBs) and activating CHK2/p53, whereas ATR is critical for sensing DNA replication stress and activating CHK1 [53]. Consistently, previous studies have shown that DDX5 binds to ATM and ATR [54], and that ATM binds to DDX5 [55] (Fig. 1K).

#### Example 2

DDX5/p68 has been reported to bind and activate RAD18 [19] (Fig. 1L1), while DDX5/p68 binds to RAD21 with unknown effects [56] (Fig. 1L2). RAD18, an E3 ubiquitin ligase, monoubiquitinates PCNA to recruit translesion synthesis (TLS) polymerases [57], whereas RAD21, a subunit of the cohesin complex, participates in HR-mediated DSB repair, sister chromatid cohesion, and chromatin organization [58]. Therefore, DDX5 binding to RAD18 and RAD21 (Fig. 1L) may play important roles in RAD18/21-mediated DNA repair functions and related cellular processes.

#### Example 3

p53 is a well-known tumor suppressor and transcription factor that induces cell cycle arrest, apoptosis or senescence, and transcriptionally activates DNA repair genes. These functions position p53 within a complex, context-dependent regulatory network that operates differently in normal versus cancer cells [59–61]. In this context, several publications have reported that p53 interacts with and activates DDX5 [1]. This is consistent with the fact that DDX5 plays major roles in multiple DNA repair pathways in cancer (Fig. 1A-L). As reviewed previously, DDX5 possesses both p53-dependent and independent DNA repair and resistance functions in cancer cells [1].

Additionally, ChIP-seq data deposited in the Gene Expression Omnibus database from Basnet et al.’s work showed that DDX5 interacts with H2AX/H2AFX [62] (Fig. 1M). In this regard, while Ser-139–phosphorylated H2AX (γH2AX) is well known as a marker of DNA DSBs, evidence indicates that loss of the H2AX gene (H2AFX) locus contributes to tumor development, progression, and resistance to therapy. Thus, human H2AFX has been proposed as an excellent candidate marker to indicate susceptibility to lymphomas, leukemia, and other cancers [63] (Fig. 1M).

Collectively, findings across multiple DDR pathways highlight the broad involvement of DDX5/p68 PPIs in cancer DDR. Although important mechanistic gaps remain, current evidence supports the concept that DDX5 contributes to diverse DNA repair processes in cancer and represents a promising upstream molecular target for therapeutic intervention.

### UbE2T in FA DNA repair

As previously discussed, in contrast to the involvement of DDX5 in most DNA repair pathways, ubiquitin-conjugating enzyme E2T (UbE2T; also called FANCT in the FA pathway) is primarily involved in the Fanconi anemia (FA) DNA repair pathway that repairs DNA interstrand crosslinks (ICLs). Several FA pathway proteins (UbE2T is one of them; see below) are emerging as master regulators of genomic integrity that can coordinate multiple other DNA repair processes [64].

Furthermore, carcinogenesis resulting from a dysregulated FA pathway is multifaceted, as FA proteins monitor multiple complementary genome-surveillance checkpoints throughout interphase. In this context, monoubiquitylation of the FANCI–FANCD2 heterodimer (ID2 complex) by the FA core complex (FANCL–FANCB–FAAP100, etc.) promotes the recruitment of DNA repair effectors to chromatin lesions to resolve DNA damage and support faithful mitosis [64, 65].

### UbE2T interacting proteins in FA pathway

UbE2T is a critical E2 ubiquitin-conjugating enzyme in the FA DNA repair pathway and is increasingly recognized as an oncoprotein due to its overexpression in many cancer types [6]. Accumulated studies indicate that UbE2T binds to and regulates several FA pathway proteins, including: (i) UbE2T binds to and activates the E3 ligase FANCL [66–68]; (ii) UbE2T binds to FANCB [69], and FANCB binds to UbE2T [70], with unclear functional consequences; (iii) UbE2T ubiquitinates FANCI in vitro [71] and binds to, and affects the expression and monoubiquitination of, FANCI in non-small cell lung cancer (NSCLC) cells [72]; and (iv) UbE2T ubiquitinates FANCD2 in vitro [71–73] and modulates the expression and monoubiquitination of FANCD2 in NSCLC cells [72].

The relationships between UbE2T and FANCL, FANCB, FANCI, and FANCD2 [66–73] are consistent with the current knowledge-based DNA interstrand crosslink (ICL) repair model (see below).

### UbE2T in ICL pathway/model

**Fig. 2:**
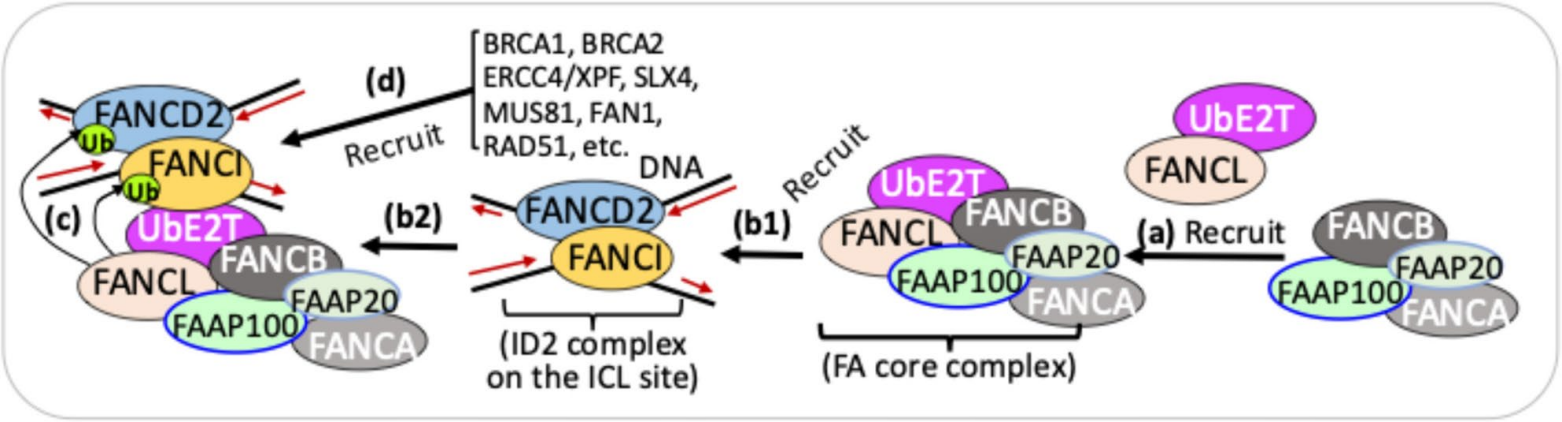
Research observation-based model for UbE2T physical and functional interactions with proteins in the FA interstrand crosslink (ICL) repair pathway: (**a**) UbE2T binds the FANCL E3 ligase to form a UbE2T–FANCL heterodimer, which then recruits FANCB, FAAP100, etc., to assemble the FA core complex (UbE2T–FANCL–FANCB–FAAP100). **b** The FA core complex is recruited to the ID2 (FANCI–FANCD2) complex at the ICL site through UbE2T–FANCI interactions. **c** FANCL in the FA core complex monoubiquitinates both FANCI and FANCD2 in coordination with UbE2T. **d** The monoubiquitinated FANCI/FANCD2 (ID2) complex at the ICL site further recruits BRCA1, BRCA2, RAD51, etc., to complete ICL repair

Interstrand crosslink (ICL) DNA damage is a particularly toxic form of DNA lesion that covalently links the two strands of the DNA helix, preventing strand separation and thereby blocking essential processes such as replication and transcription. According to the current knowledge-based model (Fig. 2), when ICL lesions form in cancer cells due to either chemotherapy (e.g., nitrogen mustards, platinum-based drugs, mitomycin C) or metabolic stress (e.g., lipid peroxidation–derived acetaldehyde or formaldehyde) [74], the UbE2T E2 enzyme interacts with the FANCL E3 ligase [75, 76] and recruits the FANCB/FAAP100 (FA-associated protein 100) complex [77], together with FANCA and FAAP20 [78] (Fig. 2a), to assemble the FA core complex [79–81] (Fig. 2b1). The FA core complex then recruits the FANCI–FANCD2 (ID2/D2-I) complex (Fig. 2b2), and FANCL monoubiquitinates both FANCD2 and FANCI within the ID2 complex (Fig. 2c), which localizes to the ICL DNA site [82–85] (Fig. 2c). Subsequently, the monoubiquitinated ID2 complex recruits additional proteins (e.g., BRCA1, BRCA2, ERCC4/XPF, SLX4, MUS81, FAN1, RAD51, etc.) (Fig. 2d) to coordinate the ICL repair process [64, 65]. A simplified Schematic for UbE2T through DNA repair-relevant proteins is provided in Supplementarty Figure S1.

### DDX5 and UbE2T in DNA repair pathway crosstalk

**Fig. 3:**
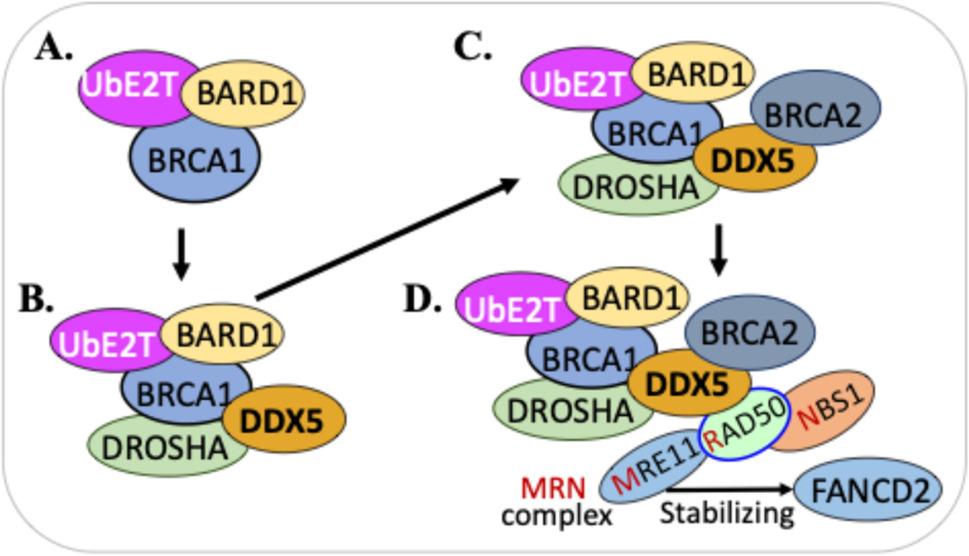
Expected interactions between UbE2T and DDX5 through DNA repair-relevant proteins: (**A**) UbE2T interacts with and colocalizes with the BRCA1 and BRCA1-associated RING domain protein 1 (BARD1) complex. **B** Indirect interaction of UbE2T with DDX5 via BRCA1. **C** Evidence-based direct and indirect interactions among UbE2T, BRCA1, DDX5, and BRCA2. **D** The MRN (MRE11–RAD50–NBS1) complex stabilizes FANCD2 and mediates DNA DSB repair

Published studies have shown that, within the FA pathway, BRCA1 functions at an early stage of the FANCI–FANCD2 (ID2) complex–involved interstrand crosslink (ICL) repair process at the DNA lesion site, whereas BRCA2 acts at a later stage of the same repair process [64]. Consistent with this, UbE2T has been shown to interact and colocalize with the BRCA1 and BRCA1-associated RING domain protein (BARD1) complex (Fig. 3A)and, through this interaction, plays a critical role in the development and progression of breast cancer [86].

Conversely, as reviewed in the section “Role of DDX5 in BRCA1 and BRCA2” for DNA double-strand break (DSB) repair, BRCA1 is directly associated with DROSHA and DDX5 [13] (Fig. 1A). Thus, BRCA1 appears to serve as a bridging factor for both DSB and ICL repair and may also establish an indirect physical link between UbE2T and DDX5, as illustrated in Fig. 3B. Additionally, BRCA2 has been shown to interact with DDX5 and to help retain DDX5 at DNA damage sites, thereby promoting HR-mediated DNA DSB repair [17] (Fig. 1B). Accordingly, potential crosstalk and interaction among these factors are anticipated, as illustrated in Fig. 3C.

Although direct evidence for DDX5 interaction with, and functional regulation of, RAD50 in cancer cells during DSB repair remains to be established, knockout of DDX5 in mouse spermatogonia led to RAD50 downregulation [19]. These studies further revealed that DDX5 is essential for proper splicing of the RAD50 gene [19], suggesting that DDX5 may functionally and/or physically interact with RAD50, as diagrammed in Fig. 1C. This observation is particularly important given that RAD50, as an integral component of the MRE11–RAD50–NBS1 (MRN) complex, senses DSBs and initiates DNA repair through HR and NHEJ mechanisms [18] (Fig. 1C). Rogues et al. demonstrated that inhibition of MRE11, RAD50, or NBS1 leads to FANCD2 destabilization [87] (Fig. 3D). Their studies further indicated that FANCD2 preferentially binds single-stranded DNA (ssDNA) over other DNA substrates in vitro, associates with ssDNA in vivo, and localizes to MRN complex–processed DNA DSBs [87]. Based on these findings, the authors concluded that the MRN complex is a crucial regulator of FANCD2 stability and function during DNA DSB repair [87] (Fig. 3D). Thus, the more detailed and precise role of DDX5 in the process outlined in Fig. 3 remains to be further investigated, which may result in important findings. A simplified crosstalk depiction for the two FL118 targets (UbE2T, DDX5) through DNA repair-relevant proteins is provided in Supplementarty Figure S2.

**Fig. 4:**
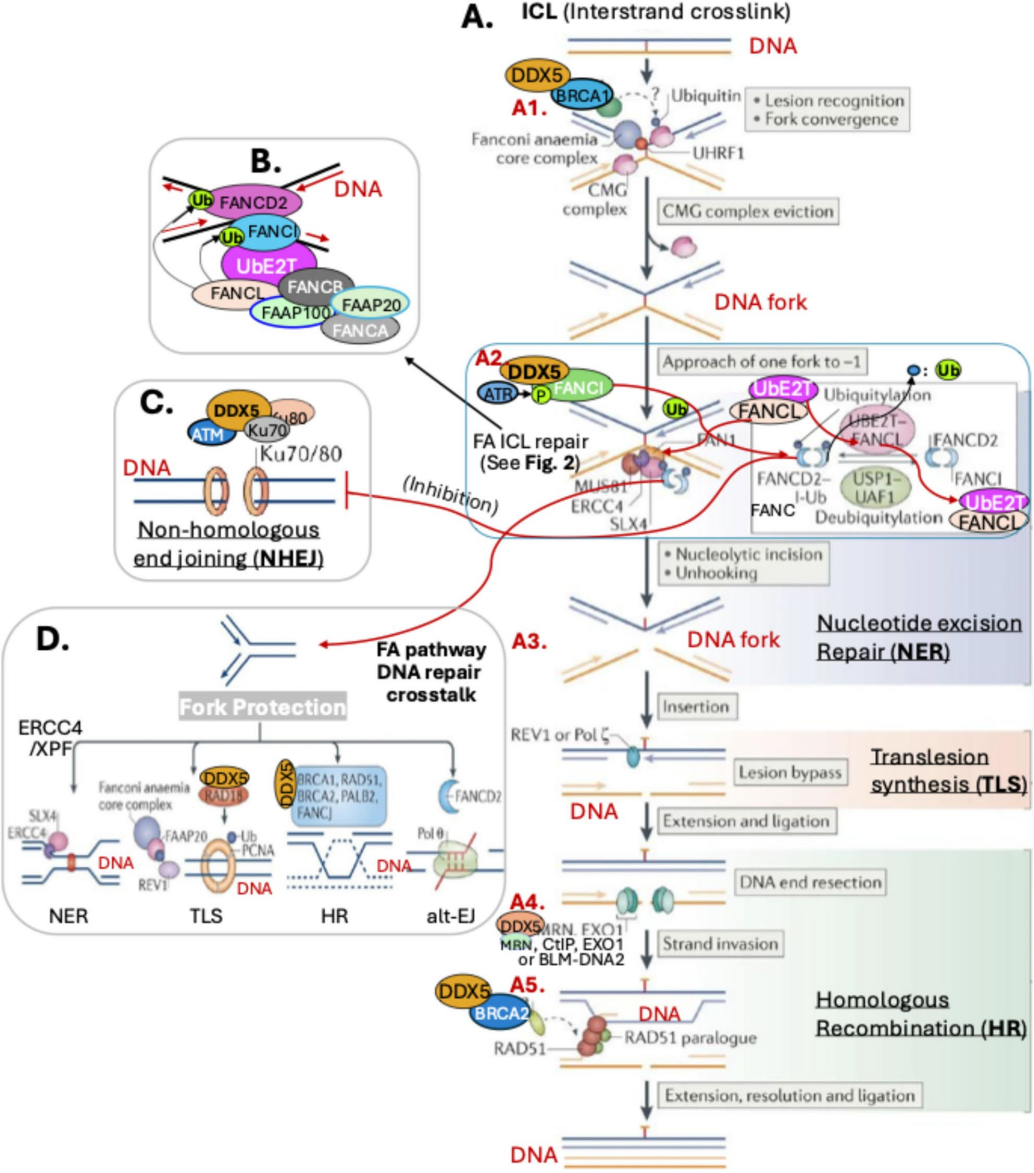
Potential linkages of DDX5 and UbE2T in network crosstalk among cancer DNA repair pathways: (**A**) Cooperation of NER, TLS, and HR proteins within a common FA ICL repair pathway. In this process, the potential involvement of DDX5 and UbE2T is highlighted (see text for the sequencing/event description). **B** Core components of the FANCD2-FANCI (D2-I/ID2) complex. **C** FA pathway crosstalk with NHEJ. Evidence shows that monoubiquitinated ID2 antagonizes NHEJ through interaction with Ku70/80 proteins. **D** Crosstalk between the FA pathway and other repair processes, with potential involvement of DDX5 highlighted. Specifically: (i) FANCD2 recruits the NER factor ERCC4/XPF via SLX4, and XPA (xeroderma pigmentosum group A–complementing protein) and XPC may promote recruitment of the FA core complex as shown in (B); (ii) ID2 is activated by the TLS factor RAD18 (to which DDX5 can bind) through monoubiquitylation of proliferating cell nuclear antigen (PCNA), and FA proteins facilitate recruitment of TLS polymerases to ICL lesions through interaction of FA-associated protein 20 (FAAP20) with DNA repair protein REV1; and (iii) the HR proteins BRCA1, BRCA2, FANCJ, PALB2 (partner and localizer of BRCA2), and RAD51 are also FA proteins, and FANCD2 functions in alternative end joining (alt-EJ) by favoring recruitment of the alt-EJ factor DNA polymerase θ (Pol θ) to DNA damage sites. This figure is adapted from a review reference [64] and incorporates additional findings reviewed/summarized in Figs. 1, 2 and 3

We note that Figs. 1 and 3 do not explicitly link the protein–protein interaction (PPI) complexes to DNA. This design was intentional, aimed at simplifying individual PPI models and emphasizing PPIs across different DNA repair pathways. Based on the findings summarized in Figs. 1 and 2, and 3D, together with existing knowledge from relevant publications [49, 64, 88–92], we have now outlined the linkage of DDX5 and UbE2T to DSB and ICL repair pathway crosstalk in cancer (Fig. 4).

UbE2T is known to physically and/or functionally interact with and regulate FANCL [66–68], FANCB [69, 70], FANCI [71, 72], and FANCD2 [71–73] (Figs. 2). In addition, as discussed previously, DDX5 has been shown to bind FANCI [51] (Fig. 1J). It is well established that the ICL repair process generates DSB intermediates that require the RAD50/MRN complex to coordinate the C-terminal binding protein (CtBP)–interacting protein (CtIP), thereby mechanistically linking ICL repair to HR-mediated DSB repair [93, 94]. Together, these observations strongly suggest extensive crosstalk among different DNA repair pathways. Indeed, accumulating evidence indicates functional crosstalk between the FA pathway and DNA HR, NER, translesion synthesis (TLS), and alternative end joining (alt-EJ), also referred to as microhomology-mediated end joining (MMEJ) [64] (Fig. 4A-D).

Specifically, activation of the FA core complex and monoubiquitylation of the ID2/D2–I heterodimer are promoted by the FANCL E3 ligase and its partner UbE2T (Fig. 4A1, B). ICLs arising during S phase impede replication fork progression, causing leading strands to pause approximately 20–40 nucleotides away from either side of the ICL (Fig. 4A1). Eviction of the replicative helicase CMG (Cdc45–MCM–GINS) complex through the action of BRCA1 allows one replication fork to approach within one nucleotide of the ICL, thereby maintaining replication fork integrity under stress conditions such as oncogene activation and/or chemotherapy (Fig. 4A1). Monoubiquitylated FANCD2 and FANCI are then directed to the ICL region, where they function as a landing pad for recruitment of multiple factors, including SLX4 and Fanconi anemia nuclease 1 (FAN1), and coordinate nucleolytic incisions mediated by ERCC4 (also known as XPF) and possibly MUS81 (an endonuclease that forms a heterodimer with EME1 or EME2 to resolve DNA structures) (Fig. 4A2, B), while concurrently suppressing NHEJ (Fig. 4C).

Unhooking of the crosslink leaves the crosslinked nucleotide tethered to the complementary strand, which is bypassed by TLS polymerases such as REV1 or DNA polymerase ζ. Subsequent ligation restores an intact DNA duplex, which then serves as a template for HR-mediated repair of the resulting DSB (Fig. 4A3). DNA incisions generate a DSB that is further processed by CtIP, the MRN complex (MRE11–RAD50–NBS1), exonuclease 1 (EXO1), and the helicase-nuclease complex BLM–DNA2 (Bloom syndrome protein–DNA replication ATP-dependent helicase/nuclease 2), generating a single-stranded DNA (ssDNA) overhang (Fig. 4A4). This ssDNA, coated with replication protein A (RPA), becomes a substrate for RAD51-mediated strand invasion promoted by BRCA2, ultimately leading to homologous recombination (Fig. 4A5). A simplified crosstalk diagram for the two FL118 targets (DDX5, UbE2T) in cancer DNA repair together with DNA repair-relevant proteins is provided in Supplementarty Figure S3.

Based on the combined diagram of DNA repair pathway crosstalk (Fig. 4), the FANCD2–FANCI (D2-I/ID2) complex appears to play a critical role across multiple DNA repair pathways (Fig. 4A2). Consistently, very recent studies by Alcon et al., using single-molecule imaging of the D2-I complex, revealed that the D2-I clamp slides along DNA at replication forks and stalls at single-stranded–double-stranded (ss-ds) DNA junctions when replication forks encounter DNA lesions [95, 96]. Their data demonstrate important roles for the D2-I complex in the recognition and protection of stalled replication forks across several DNA repair pathways [95].

Notably, it has been shown that DDX5 binds to FANCI [51], whereas UbE2T binds to and activates the E3 ligase FANCL [66–68], thereby promoting FANCL-mediated monoubiquitination and influencing the expression of FANCD2 and FANCI (Fig. 2) [71–73]. Together, these findings highlight that future studies aimed at elucidating the roles of DDX5 and UbE2T in D2-I complex function would be highly informative and impactful to biomedical research and cancer therapeutics.

Collectively, the interplay among these DNA repair pathways (Fig. 4) suggests that DDX5 and UbE2T may act as central coordinators that promote DNA repair, thereby enhancing cancer cell survival and resistance to treatment. However, this conclusion leaves many knowledge gaps that need to be filled through further investigation.

### DDX5 and topoisomerases

**Fig. 5:**
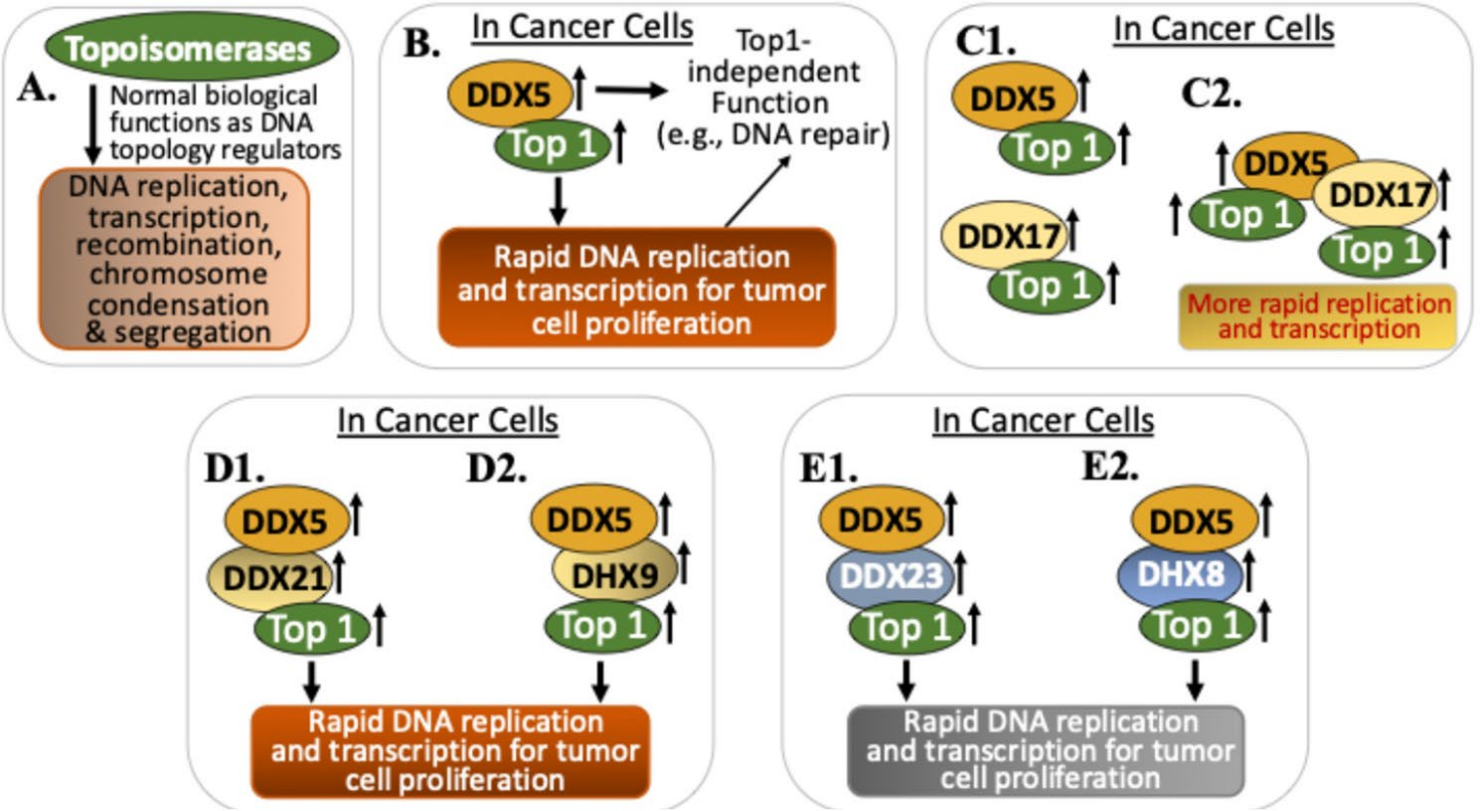
Physical binding relationship between DDX5 and topoisomerase 1 (Top1): (**A**) Topoisomerases regulate DNA topology and are involved in DNA replication, transcription, recombination, chromosome condensation, and segregation. **B** DDX5 and Top1 are elevated in cancer cells. While the functional consequences of DDX5-Top1 binding remain to be elucidated, elevated Top1 in cancer cells can increase cancer cell proliferation and treatment resistance. **C** The DDX5 paralog DDX17 was found to bind Top1 and can also form a heterodimer with DDX5. **D** Both DDX21 and DHX9/DDX9 were found to bind Top1, and DDX5 can bind both DDX21 and DHX9/DDX9. **E** Both DDX23 and DHX8/DDX8 were found to bind Top1, and DDX5 can bind both DDX23 and DHX8/DDX8

It is well established that topoisomerases are DNA topology regulators that cut and rejoin DNA strands to relieve torsional stress during DNA replication, transcription, recombination, chromosome condensation, and chromosome segregation [97] (Fig. 5A). Although topoisomerases are not formally classified as DNA repair proteins, they play crucial supporting roles in maintaining genome stability and can be directly involved in DNA repair processes, particularly in the case of topoisomerase 1 (Top1) [97]. In addition, topoisomerase malfunctions and associated phenotypic dysfunctions have been shown to induce various pathological conditions [97]. While DDX5 appears to play a critical role in multiple DNA repair pathways in cancer cells (Fig. 4), its physical and functional positioning as a potential key partner in topoisomerase function has not been comprehensively reviewed. Below, we focus on this aspect as an introductory effort to guide future research.

### DDX5 with top1

DDX5 was first shown to bind Top1 in 2005 using co-immunoprecipitation (co-IP) and affinity chromatography combined with mass spectrometry (MS) analyses [98]. This physical interaction between DDX5 and Top1 was rediscovered in 2022 during a systematic analysis of human protein localization and interactions using combined genome engineering, confocal live-cell imaging, MS, and integrated data analyses [99]. Although the precise role of DDX5 in Top1-mediated mechanisms remains to be defined, DDX5 - an oncoprotein highly expressed in cancer with multiple critical functions [1] - may act as a guiding factor to ensure accurate DNA replication and transcription in cancer cells. In addition, DDX5 may facilitate Top1 involvement in these processes and, in certain contexts, participate in DNA repair (Fig. 5B), potentially in a manner analogous to the interaction between Myc and topoisomerases [100].

Beyond DDX5, its paralog DDX17 has also been shown to physically interact with Top1, although the functional significance remains unclear [98]; this interaction was likewise rediscovered recently [99]. The physical interaction of Top1 with both DDX5 and DDX17 may play important roles in cancer malignancy. DDX5/p68 and DDX17/p72 are known to heterodimerize in cells [101], exhibit overlapping functions [102, 103], and dynamically orchestrate the regulation of numerous cellular processes [104, 105], including promotion of human viral functions [106, 107]. The roles of DDX5 and DDX17 in tumorigenesis and cancer progression have been reviewed previously [108]. In addition, physical binding between DDX5 and DDX17 has been identified in multiple global or large-scale protein interaction studies [69, 70, 99, 109–112]. Taken together, two mechanistic models are conceivable, as depicted in Figs. 5C1 and 5C2. Regardless of the specific model, the interactions of DDX5 and DDX17 with Top1 may facilitate Top1 function in supporting DDX5- and DDX17-mediated regulation of rapid DNA replication and transcription in cancer cells and, in certain contexts, contribute to DNA repair, thereby promoting treatment resistance.

In addition to DDX5 and DDX17, co-IP and affinity chromatography combined with MS analyses revealed binding of Top1 with DDX21 and DHX9/DDX9 [98]. The interaction between Top1 and DHX9 was also rediscovered recently [99]. The relevance of these findings lies in the fact that.

DDX5 binds to DDX21 [113] and DHX9 [99, 109, 113–115], and that the roles of DDX21 [116–120] and DHX9 [114, 121–124] overlap substantially with those of DDX5 [1], including promotion of DNA damage repair, resolution of R-loops, and regulation of gene transcription. Therefore, DDX5 may play a critical role in coordinating and/or controlling DDX21 and DHX9 in their biological functions related to Top1 (Fig. 5D).

Similarly, proximity-dependent biotin identification revealed that DDX23 binds to both DDX5 and Top1 [125] (Fig. 5E1). In addition, co-IP and MS analyses demonstrated binding of DDX5 to DDX23 [54] and DHX8/DDX8 [54], as well as binding of Top1 to DHX8 [99] and DDX23 [99] and DDX23 [98, 99] (Fig. 5E1, E2). Although relatively few studies are available, DDX23 appears to function as an oncogenic protein that promotes cancer development and metastasis [126–128], showing notable similarities to DDX5 [1, 108, 129, 130]. In contrast, DHX8 has been reported in fewer than ten studies, with only two linking DHX8-associated genetic alterations to human breast, ovarian, or lung cancers [131, 132].

Based on the reviewed observations which suppoprt both direct and indirect interactions between DDX5 and Top1; more detailed studies are still needed to define their functional dependencies governing their significance in cancer biology.

### DDX5 with Top2A (Top2α), Top2B (Top2β) and TopBP1

Top1 introduces transient single-strand DNA breaks, whereas Top2A and Top2B introduce transient double-strand breaks. There is currently no strong evidence that DDX5 directly binds Top2A or Top2B. However, several DDX5-interacting proteins within the DEAD-box RNA helicase family have been shown to directly bind Top2A, Top2B, and/or TopBP1 (Top2-binding protein 1), including DDX1, DHX9/DDX9, DDX18, DDX21, and DDX23.

**Fig. 6:**
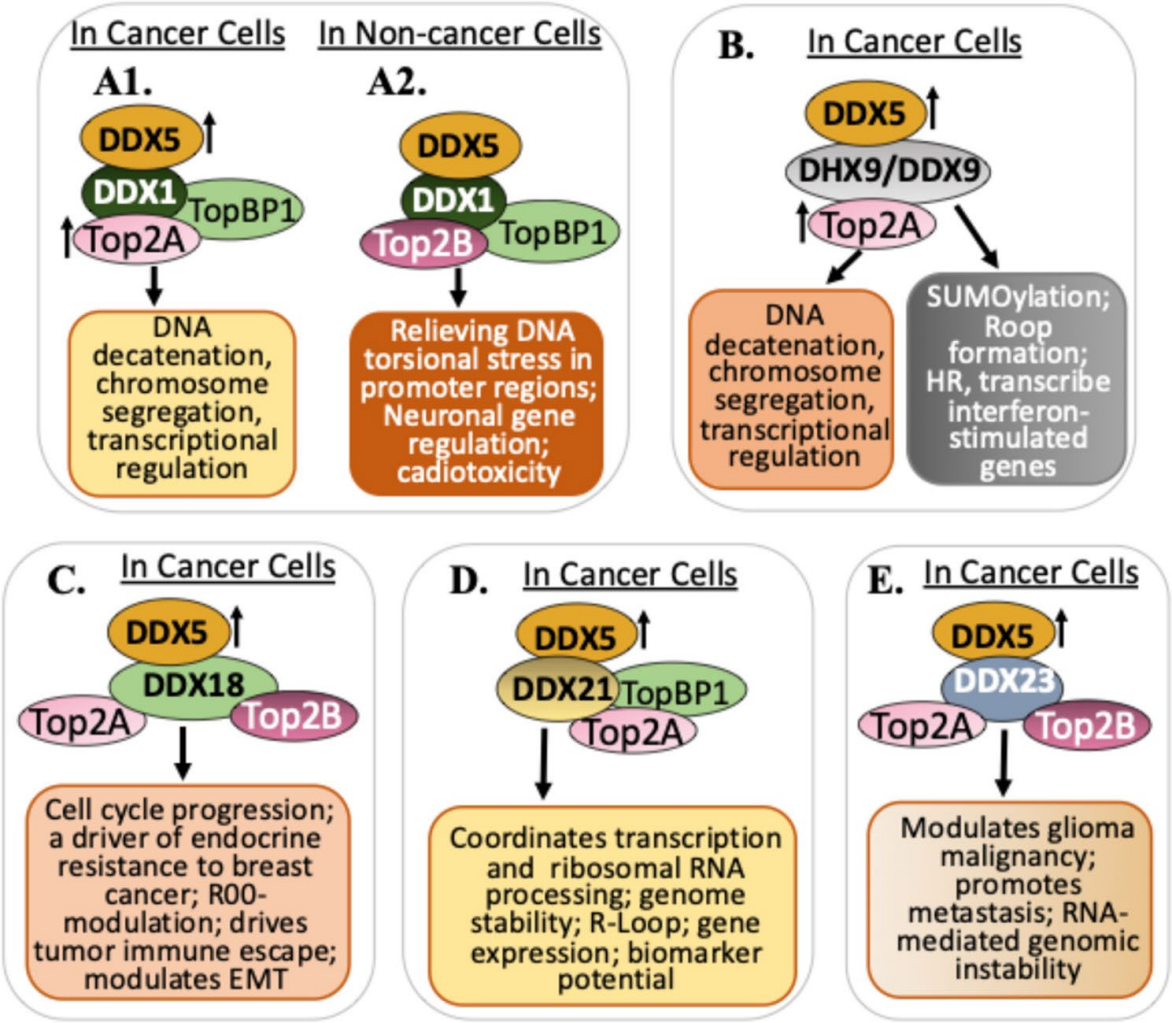
Physical binding relationship between DDX5 and topoisomerase 2 A (Top2A) and Top2B: (**A**) DDX1 was shown to bind Top2A, Top2B, and TopBP1, and DDX5 was shown to bind DDX1. Top2A is mainly involved in proliferating/cancer cells, whereas Top2B is more prominent in non-proliferating and differentiated cells. **B** DHX9/DDX9 was shown to bind Top2A, and DDX5 was shown to bind DHX9/DDX9. **C** DDX18 was shown to bind Top2A and Top2B, and DDX5 was shown to bind DDX18. **D** DDX21 was shown to bind Top2A and TopBP1, and DDX5 was shown to bind DDX21. **E** DDX23 was shown to bind Top2A and Top2B, and DDX5 was shown to bind DDX23

DDX5 and DDX1 were recently shown to physically interact [113, 133], and DDX1 also binds Top2A, Top2B, and TopBP1 [134]. Based on the known functions of Top2A, Top2B, and TopBP1, interactions mediated by DDX1 may allow DDX5 to modulate their activities in proliferating cancer cells (Fig. 6A1) as well as in non-proliferating and differentiated cells (Fig. 6A2). Although DDX1 participates in a broad range of pathophysiological processes, its functions partially overlap with those of DDX5 [1, 108, 129, 130], including serving as a prognostic marker in cancer [135, 136], contributing to cancer stem cell–associated treatment resistance [137], and participating in R-loop processing [138–141]. Notably, several studies have also reported opposing roles for DDX1 in cancer [142, 143], providing an intriguing opportunity to explore how DDX5 may modulate DDX1 function.

Zhou et al. reported that DHX9/DDX9 physically interacts with mouse Top2A and enhances Top2A activity [144]; interaction between DHX9 and human Top2A was also demonstrated recently [114]. The relevance of these observations lies in the binding of DDX5 to DHX9 [99, 109, 113–115] and the extensive overlap between DHX9 and DDX5 functions [1, 108, 129, 130] (Fig. 6B).

Shi et al. reported that DDX18 binds to DDX5, Top2A, and Top2B, although the functional consequences were not initially defined [145]. Subsequent studies demonstrated that DDX18 is essential for cell-cycle progression in zebrafish hematopoietic cells and is mutated in human acute myeloid leukemia (AML) [146]. Additional studies identified DDX18 as a driver of endocrine resistance in breast cancer [147], a promoter of viral infection [148], a suppressor of R-loop–induced DNA damage and genome instability [149], a regulator of tumor immune escape [150], and a modulator of epithelial–mesenchymal transition (EMT) [151] (Fig. 6C). These functions largely overlap with those of DDX5 [1, 108, 129, 130].

In addition to binding DDX5 [113] and Top1 [98], DDX21 also binds Top2A [152] and TopBP1 [153]. As discussed previously, DDX21 functions substantially overlap with those of DDX5 [1, 108, 129, 130] (Fig. 6D). Similarly, beyond binding DDX5 [54, 125] and Top1 [98, 99, 125], DDX23 also binds Top2A and Top2B [125]. Although studies on DDX23 remain limited, available evidence suggests that DDX23 functions as an oncogenic protein promoting cancer development and metastasis [126–128] (Fig. 6E), again showing similarities to DDX5 [1, 108, 129, 130].

In short, current data indicate the indirect interaction of DDX5 with Top2A, Top2B and/or TopBP1 through DDX1, DDX9, DDX18, DDX21 and DDX23 (Fig. 6). However, the role of DDX5 in DDX1/DDX9/DDX18/DDX21/DDX23-mediated regulation of the function ofTop2A, Top2B and/or TopBP1 remains to be elucidated.

### DDX5 with Top3B (Top3β)

Using Flag antibody–mediated immunoprecipitation of Flag-tagged Top3β expressed in *Drosophila* embryonic S2 cells followed by MS analysis, Lee et al. identified p68/DDX5 as a Top3β-associated protein [154]. Their studies suggested that the Top3β–p68 interaction is partially RNA dependent, as RNase A treatment reduced the interaction by approximately tenfold [154]. The authors proposed that p68/DDX5 contributes to resolving complex RNA topological structures during heterochromatin formation [154].

**Fig. 7:**
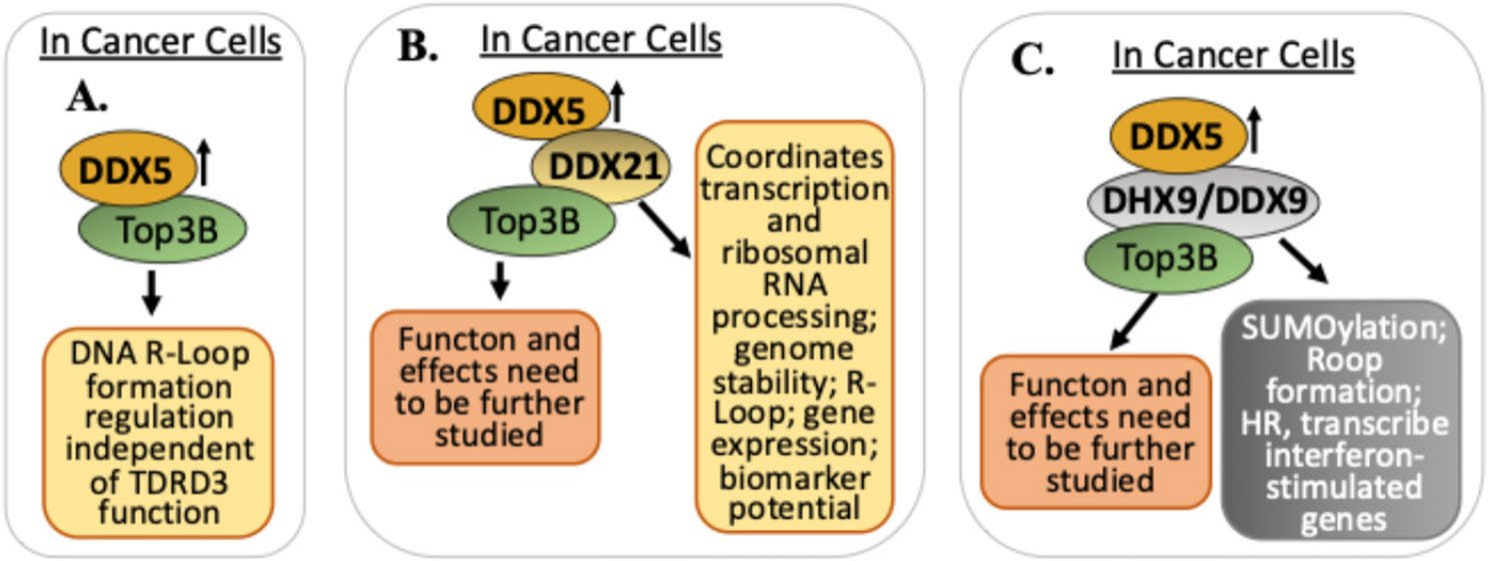
Physical binding relationship between DDX5 and topoisomerase 3B (Top3B): (**A**) DDX5 was shown to bind Top3B and support Top3B-mediated R-loop resolution. **B** DDX21 was shown to bind Top3B and TopBP1, and DDX5 was shown to bind DDX21. **C** DHX9/DDX9 was shown to bind Top3B and TopBP1, and DDX5 was shown to bind DHX9/DDX9

Consistent with these findings, Saha et al. identified DDX5/p68 as a major Top3B-interacting helicase after immunoprecipitating endogenous Top3B followed by MS analysis [155]. This interaction was validated using complementary approaches, including expression of Flag-tagged Top3B in Top3B knockout HCT116 cells and HEK293 cells under conditions with or without drug-induced R-loop formation. These studies demonstrated that the DDX5–Top3B interaction occurs in both cell models and is independent of R-loop formation [155] (Fig. 7A). Further analyses revealed that the DDX5–Top3B interaction and chromatin localization of DDX5 are independent of Tudor domain–containing protein 3 (TDRD3) [155] (Fig. 7A). In *Drosophila*, TDRD3 functions primarily as a scaffold protein dispensable for p68/DDX5–Top3β interaction [154]. In contrast, in cancer cells, TDRD3 functions as an epigenetic reader (e.g., recognizing H3R17me2a and H4R3me2a histone marks), a transcriptional coactivator [156], and a promoter of breast cancer progression [157, 158].

In summary, the physical interaction and functional relationship of DDX5 with Top3B are defined. However, additional studies will be needed to extend the current knowledge scope in this specialized area.

### Functional implications and therapeutic relevance

We have reviewed interactions between DDX5 and DDX21 [113] (Fig. 5D1), DHX9 [99, 109, 113–115] (Fig. 5D2) and Top1 [98, 99] (Fig. 5B), as well as interactions of DDX21 with Top1 [98] (Fig. 5D1), Top2A [152], and TopBP1 [153] (Fig. 6D), and interactions of DHX9 with Top1 [98, 99] and Top2A [114, 144] (Figs. 5D2 and 6B). Based on these findings, the functional roles of DDX21 [116–120] and DHX9 [114, 121–124] substantially overlap with those of DDX5 [1, 108, 129, 130]. In addition to DDX5 directly binding Top3B [155] (Fig. 7A), both DDX21 and DHX9 also bind Top3B [155] (Fig. 7BC).

Collectively, these observations suggest that DDX5 may play a critical role in coordinating topoisomerase functions and associated regulatory processes. Accordingly, degradation of DDX5 by FL118 [41] could affect Top1 activity, particularly when FL118 is administered at concentrations or doses substantially higher than those required for therapeutic efficacy in cancer treatment [159]. However, consistent with the broader functional roles of DDX5 beyond topoisomerase regulation, we demonstrated that, unlike the Top1 inhibitors topotecan and irinotecan/SN38, FL118 antitumor sensitivity in colorectal cancer (CRC) is independent of Top1 expression. Specifically, CRC tumors with low or undetectable Top1 expression can be highly sensitive to FL118, whereas tumors with high Top1 expression can be insensitive to FL118 [160].

Although topoisomerases have long served as therapeutic targets in cancer, their ubiquitous expression in both cancer and normal cells limits their potential as selective and durable targets. Topoisomerase poisons can induce severe DNA damage in normal tissues (e.g., hematopoietic cells), leading to dose-limiting toxicities and increased risk of secondary malignancies during long-term clinical use. Therefore, despite emerging paradigms for targeting topoisomerases, there remains a critical need for alternative, cancer-selective strategies to modulate topoisomerase function [161]. One such approach is targeting cancer-overexpressed oncogenic proteins such as DDX5 and UbE2T, which play central roles in promoting cancer DNA repair and other oncogenic functions, with or without direct involvement of topoisomerases.

### DDX5, EZH2, NANOG WITH NEPC, CRPC, mCRPC AND PCSC

Although prostate cancer (PCa) is not typically difficult to treat in its early stages, it can become highly refractory in advanced forms, including castration-resistant PCa (CRPC), metastatic CRPC (mCRPC), and neuroendocrine PCa (NEPC). Our recent review highlighted the potential role of DDX5 in CRPC and mCRPC and discussed the novel anticancer agent FL118 as a promising strategy to overcome intrinsic cell heterogeneity and treatment-induced cellular plasticity associated with these advanced diseases [1]. In this section, we review the potential importance of DDX5 in regulating, and working together with, EZH2 and NANOG in the development and maintenance of NEPC, CRPC, and mCRPC, as well as the role of prostate cancer stem cells (PCSCs) in advanced/lethal PCa.

### DDX5 and EZH2

**Fig. 8:**
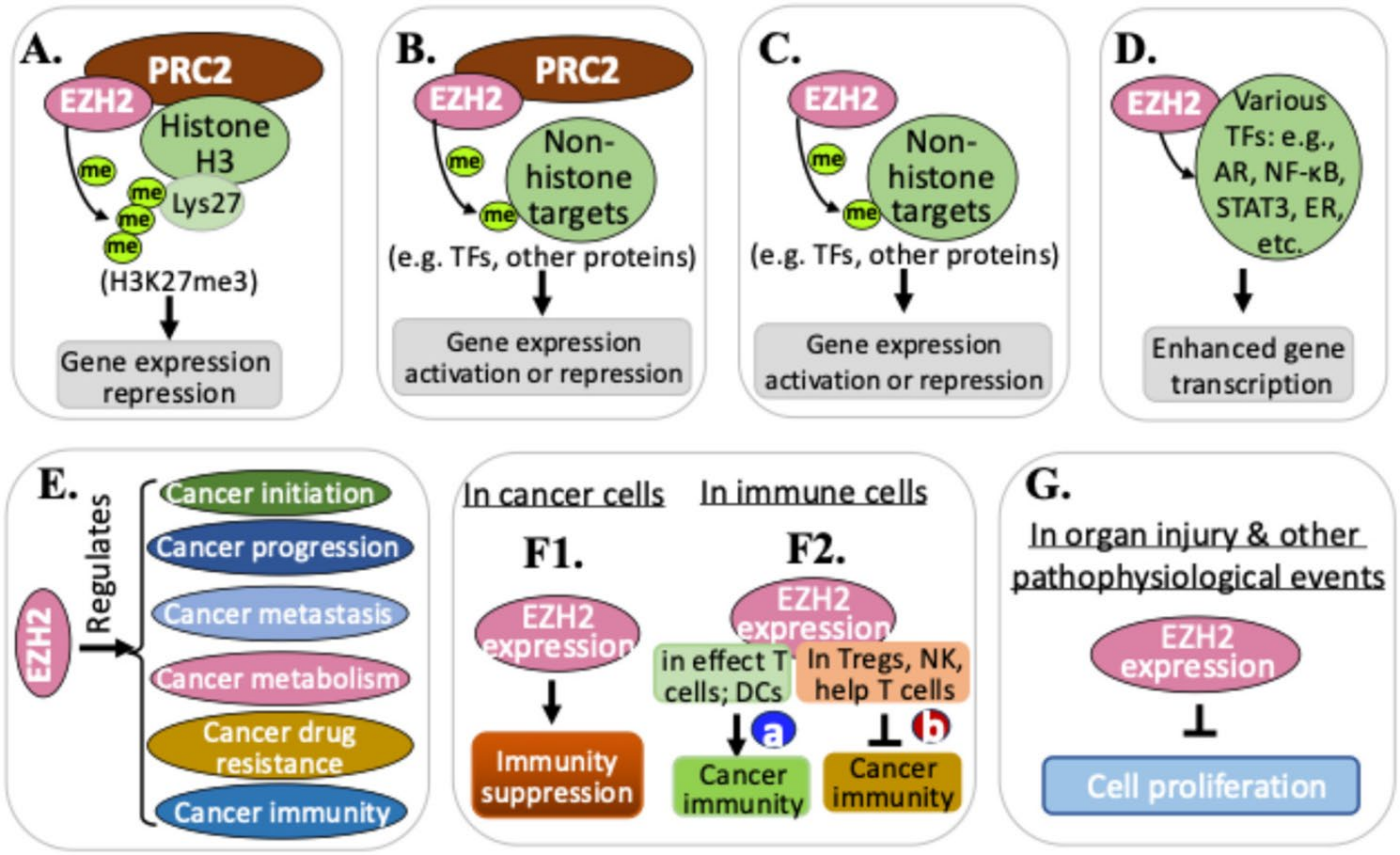
Mechanism of action (MOA) and functions of EZH2: (**A**) PRC2- and methylation-dependent EZH2 trimethylation of histone H3 at Lys27 (H3K27me3) to repress gene expression. **B** PRC2- and methylation-dependent EZH2 methylation of non-histone protein targets to repress or activate gene expression. **C** PRC2-independent and methylation-dependent EZH2 methylation of non-histone protein targets to repress or activate gene expression. **D** PRC2- and methylation-independent EZH2 interactions with transcription factors (TFs) to enhance gene transcription. **E** Summary of EZH2 functions. **F** Differential consequences of EZH2 expression in cancer cells versus immune cells. **G** EZH2 can also act as a tumor suppressor gene in specific contexts, such as organ injury and other pathophysiological events

EZH2 is overexpressed in many solid tumors, including aggressive PCa. EZH2 executes its functions through at least four mechanisms of action (MOA): (i) EZH2 functions as the enzymatic catalytic subunit of polycomb repressive complex 2 (PRC2) to repress PRC2 downstream target gene expression by trimethylation of lysine 27 on histone H3 (H3K27me3) (Fig. 8A); (ii) PRC2-dependent and EZH2-mediated methylation of non-histone protein substrates such as transcription factors (TFs) or other signaling proteins (Fig. 8B; less common); (iii) PRC2-independent but EZH2-mediated methylation of other non-histone substrates to activate downstream target genes (Fig. 8C; more common); and (iv) PRC2- and methylation-independent functions of EZH2 as a transcriptional activator (co-factor/co-activator) [162] (Fig. 8D). Through its canonical and non-canonical MOAs, EZH2 functions as a master regulator of cancer initiation, progression, metastasis, metabolism, drug resistance, and immunity [162] (Fig. 8E), as well as virus-mediated oncogenesis [163–165], features that closely parallel the phenotypic characteristics of DDX5 [1].

However, EZH2 can behave opposing functions in tumor microenvironment (TME). EZH2 expression in cancer cells can enhance immune suppression, whereas inhibition of EZH2 expression can favor antitumor immunity (Fig. 8F1). In contrast, EZH2 expression in immune cells such as effector T cells and dendritic cells (DCs) can promote anticancer immunity (Fig. 8F2a), whereas EZH2 expression in regulatory T cells (Tregs), helper T (Th) cells, and natural killer (NK) cells can suppress anticancer immunity [162] (Fig. 8F2b). Additionally, during organ injury and other pathophysiological events, EZH2 may function as a tumor suppressor to block cell proliferation [162] (Fig. 8G). These complex regulatory effects may provide EZH2 with greater potential as a therapeutic target in cancer.

These concepts largely apply to advanced/lethal PCa. EZH2 is highly upregulated in aggressive PCa and plays an important role in PCa progression toward NEPC [166]. For example, a recent study showed that EZH2 modulates bivalent genes, resulting in upregulation of NEPC-associated transcriptional drivers (e.g., ASCL1) and neuronal gene programs in NEPC, and that targeting EZH2 in NEPC leads to forward differentiation [167]. This finding is consistent with prior evidence that NEPC arises clonally from prostate adenocarcinoma (PRAD) with shared genomic features [168], including acquisition of distinct changes in DNA methylation and chromatin accessibility associated with dysregulation of lineage-determining TFs that distinguish NEPC from PRAD [168–171].

Given EZH2 potential as a therapeutic target in advanced/lethal PCa and other cancers, there is growing interest in developing EZH2 inhibitors (EZH2i), including EZH2 protein degraders [162, 166]. However, rather than pursuing EZH2i monotherapy, current clinical trials primarily evaluate EZH2i in combination with other therapies. For example, Pfizer’s mevrometostat (PF-06821497) has recently been reported in combination clinical trials with enzalutamide in patients with mCRPC (https://ascopubs.org/doi/10.1200/JCO.2025.43.5_suppl.LBA138), and several additional clinical trials are planned in mCRPC (NCT03460977; NCT06551324; NCT06629779; NCT07028853). Similarly, Ipsen’s tazemetostat is being advanced into combination trials with talazoparib in mCRPC (NCT04846478), following its evaluation in other cancers such as malignant pleural mesothelioma [172] (NCT02860286).

Despite these developments, the physical and functional relationship between EZH2 and DDX5 remains unclear and has received relatively little attention. Based on currently available literature, DDX5 may play important roles in mutual regulation of EZH2 and may even directly control aspects of EZH2 function. Our highlights on this topic are summarized below.

**Fig. 9:**
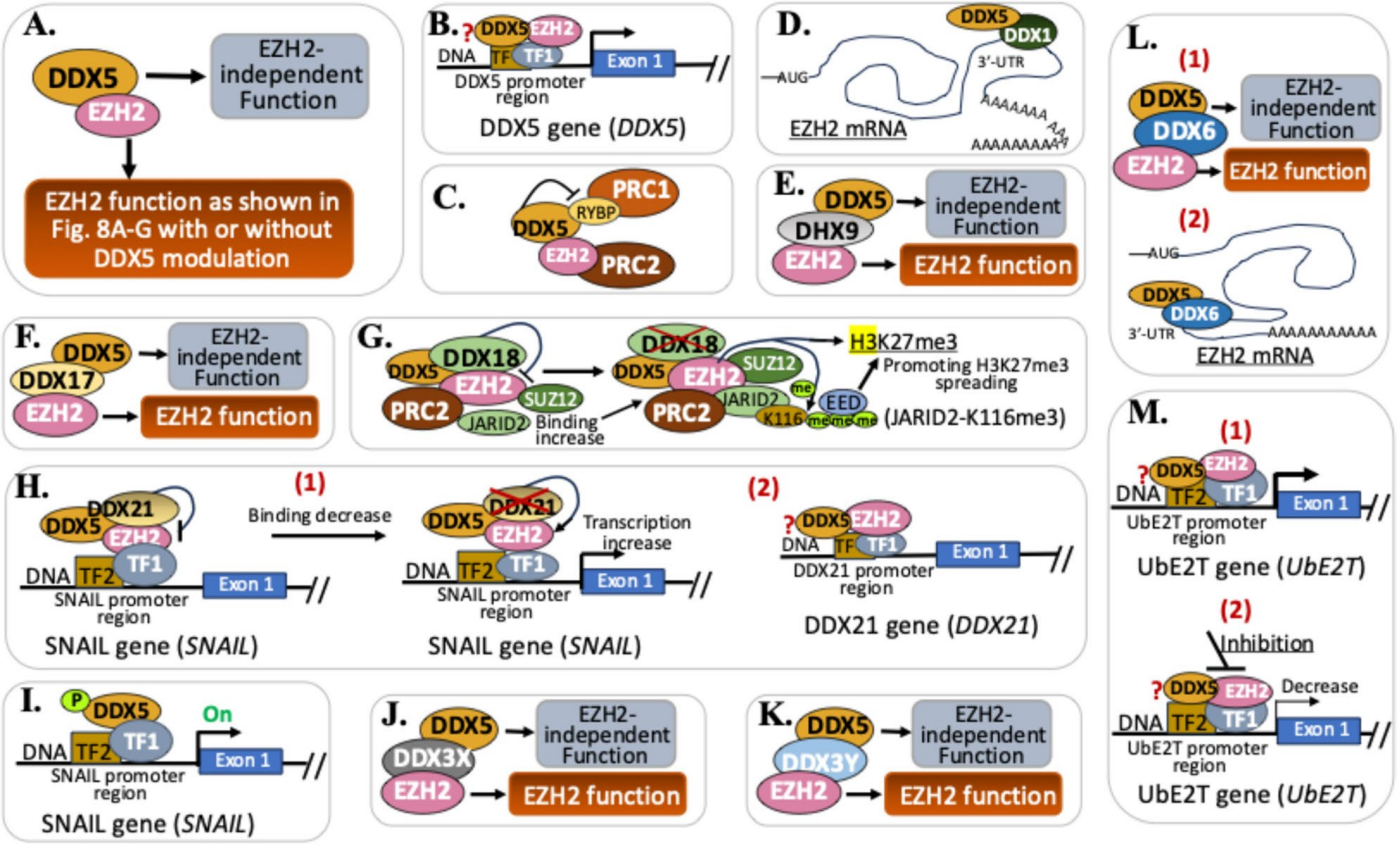
Physical binding relationship between DDX5 and EZH2: (**A**) DDX5 was shown to bind EZH2. **B** EZH2 binds to the promoter region of the DDX5 gene (DDX5); DDX5 binding to EZH2 may feedback-modulate EZH2 functions. **C** DDX5 binds EZH2 (the catalytic subunit of PRC2) and also binds to and inhibits RYBP (a PRC1 subunit) in non-cancer stem cells. **D** DDX1 binds EZH2 mRNA; DDX5 binding to DDX1 may modulate DDX1 functions, including EZH2 mRNA stability. **E** DHX9/DDX9 binds EZH2; DDX5 binding to DHX9 may modulate DHX9 functions on EZH2. **F** DDX17 binds EZH2; DDX5 binding to DDX17 may modulate DDX17 functions on EZH2. **G** DDX18 binds EZH2; DDX18 ablation increases Suz12 and Jarid2 binding to EZH2 for spreading H3K27me3, which may be modulated by DDX5 binding to both DDX18 and EZH2. **H** DDX21 binds EZH2, and EZH2 binds the promoter of SNAIL (SNAIL); DDX21 ablation increases SNAIL transcription (1). As a feedback loop, EZH2 also binds the DDX21 promoter; thus, DDX5 binding to both DDX21 and EZH2 may modulate EZH2 functions (2). **I** DDX5 binds the SNAIL promoter to increase SNAIL transcription. **J** DDX3X binds EZH2; DDX5 binding to DDX3X may modulate DDX3X functions on EZH2. **K** DDX3Y binds EZH2; DDX5 binding to DDX3Y may modulate DDX3Y functions on EZH2. **L** DDX6 binds EZH2; DDX5 binding to DDX6 may modulate DDX6 functions on EZH2 (1). Meanwhile, DDX6 also binds EZH2 mRNA, and DDX5 binding to DDX6 may modulate DDX6 functions such as EZH2 mRNA stability (2). **M** EZH2 binds the promoter region of the UbE2T gene (UbE2T) (1), and EZH2 inhibition decreases UbE2T transcription (2)

Using EZH2 or EED (a core scaffolding subunit of PRC2) antibody immunoprecipitation (IP) to pull down binding proteins from VCaP PCa cells followed by MS analysis, it was found that DDX5 physically binds EZH2 and EED [173]. Mutual binding between DDX5 and EZH2 has been rediscovered in breast cancer MCF-7 and MDA-MB-231 cells [174], glioblastoma U87 cells [175], and human umbilical vein endothelial cells [176] (Fig. 9A). Interestingly, ChIP-sequencing revealed binding of EZH2 to the DDX5 promoter region in atypical teratoid rhabdoid tumor tissue [177] (Fig. 9B), suggesting that EZH2 may transcriptionally regulate DDX5 expression. This observation adds another layer through which DDX5/EZH2-associated interaction networks may be coordinated.

PRC2 catalyzes the H3K27me3 histone mark, which serves as a binding platform for PRC1 (core: BMI1 + RING1B). PRC1, in turn, catalyzes histone H2A monoubiquitination at lysine 119 (H2AK119ub1), which helps PRC2 spread and maintain H3K27me3 for long-term transcriptional silencing. In this context, Cao et al. found that anti-RING1B antibodies pulled down BMI1 and DDX5, but not EZH2 and EED, whereas anti-BMI1 antibodies pulled down RING1B but not DDX5, EZH2, or EED from VCaP PCa cells [173]. While these observations demonstrate binding specificity, they also suggest that DDX5 interacts with both PRC1 and PRC2 complexes (Fig. 9C), potentially contributing to coordination and functional interplay between these polycomb complexes. Notably, a previous study reported that DDX5 binds to and inhibits RYBP, a PRC1 subunit, in embryonic stem cells [178] (Fig. 9C).

Furthermore, we have reviewed physical and functional interactions of DDX5 with DDX1 (Fig. 6A), DHX9/DDX9 (Figs. 5D2, 6B, 7 C), DDX17 (Fig. 5C2), DDX18 (Fig. 6C), and DDX21 (Figs. 5D1, 6D, 7B), several of which can bind one or more topoisomerases. In this regard, DDX1 has been found to bind EZH2 mRNA in osteosarcoma U2OS cells [134], suggesting that DDX5 may cooperate with DDX1 to regulate EZH2 mRNA (Fig. 9D). EZH2 binds DHX9 in VCaP PCa cells [173] and U87 glioblastoma cells [175], suggesting that DHX9, together with DDX5, may modulate EZH2 function (or vice versa) (Fig. 9E). DDX17 is a paralog of DDX5, and in many contexts, the two form a heterodimer that cooperatively executes cellular functions [101–105]. Thus, binding of DDX17 to EZH2 [175, 176] may provide DDX5/DDX17 with additional capacity to modulate EZH2 function (Fig. 9F).

DDX18 was found to bind EZH2, and ablation of DDX18 increased EZH2 binding to SUZ12 (a core PRC2 subunit) and JARID2 (a PRC2 accessory subunit) in embryonic stem cells [179] (Fig. 9G). SUZ12 binding can stabilize EZH2 and its catalytic activity to establish H3K27me3, and JARID2 can bind PRC2, directly contact EZH2, and be methylated by EZH2 at lysine 116 (JARID2-K116me3), creating a binding site for EED that enhances EZH2 catalytic activity and promotes H3K27me3 spreading [180, 181] (Fig. 9G). Based on these findings, interactions involving DDX5-EZH2 (Fig. 9A) and DDX18 may provide DDX5 with additional regulatory capacity to modulate EZH2-related functions (Fig. 9G).

Moreover, DDX21 has been reported to bind EZH2, and ablation of DDX21 reduces EZH2 binding to the SNAIL promoter region [182], leading to increased transcription (Fig. 9H1). In turn, EZH2 binds the DDX21 promoter region in MCF-7 cells [182] and Du145 cells [173], establishing a mutual regulatory loop between DDX21 and EZH2 (Fig. 9H2). Interestingly, DDX5 not only binds DDX21 (Figs. 5D1, 6D, 7B), potentially modulating DDX21 function, but also phosphorylated DDX5/p68 has been shown to activate SNAIL transcription [183] (Fig. 9I), in contrast to DDX21, which suppresses SNAIL transcription [182] (Fig. 9H1).

In addition, DDX3X interacts with DDX5 [184] and EZH2 [173, 174] (Fig. 9J); DDX3Y binds DDX5 [185] and is bound by EZH2 [175] (Fig. 9K); and DDX6 interacts with DDX5 and binds EZH2 in glioblastoma U87 cells [186] (Fig. 9L1) and binds the EZH2 transcript (mRNA) in pancreatic cancer SW1990 cells [187] (Fig. 9L2), suggesting that DDX5 together with DDX6 may modulate both EZH2 function and expression.

Finally, ChIP-sequencing revealed binding of EZH2 to the UbE2T promoter region (Fig. 9M1), and inhibition of EZH2 decreased UbE2T expression in breast cancer cells [188] (Fig. 9M2). ChIP-sequencing also revealed EZH2-mediated regulation of UbE2T transcription in multiple myeloma patient-derived cancerous plasma cells [189] (Fig. 9M).

Collectively, these findings indicate that DDX5 can modulate EZH2 through diverse mechanisms, including direct interaction with EZH2 and indirect association mediated by other DEAD/DEAH box family proteins, providing multiple avenues through which DDX5 may control EZH2 expression, regulation, and function. This, in turn, positions DDX5 as a promising therapeutic target for difficult-to-treat cancers and highlights a research area with significant potential for high-impact discoveries.

### DDX5 and NANOG

The pluripotency TF NANOG is frequently upregulated in advanced PCa and enriched in the PCa stem-cell (PCSC) fraction, and its expression is associated with castration resistance, PCa recurrence, and poor patient outcomes [190]. NANOG drives lineage plasticity and stem-like programs by reprogramming transcriptional networks (e.g., cooperating with AR/FOXA1 at chromatin), activating EMT/stemness pathways (e.g., Wnt/β-catenin, STAT3, PI3K/AKT, etc.), and upregulating stemness/migration genes (e.g., CXCR4, IGFBP5, CD133, ALDH1), thereby promoting androgen independence, therapy resistance, and metastatic behavior [190, 191]. NANOG expression has been demonstrated to be necessary and sufficient for CRPC xenograft tumor growth and regenerative capacity [191], indicating that NANOG is not only a biomarker but also a functional driver.

Currently, direct NANOG inhibitors are not clinically available. Therefore, current clinical strategies to eliminate PCSCs focus on blocking PCSC-associated signaling pathways by targeting/modulating various pathway regulators (e.g., proteins and miRNAs) and the PCSC immune microenvironment [192]. For example, recent work reported that upregulation of NANOG, SOX2, and CD44 via NF-κB signaling plays an important role in M1 macrophage-secreted factors that drive PCSC plasticity [193]. However, the physical and functional relationship between NANOG and DDX5 has not been comprehensively reviewed. Based on current literature, DDX5 may directly and/or indirectly regulate NANOG expression and function. Below we highlight key findings supporting this concept.

**Fig. 10:**
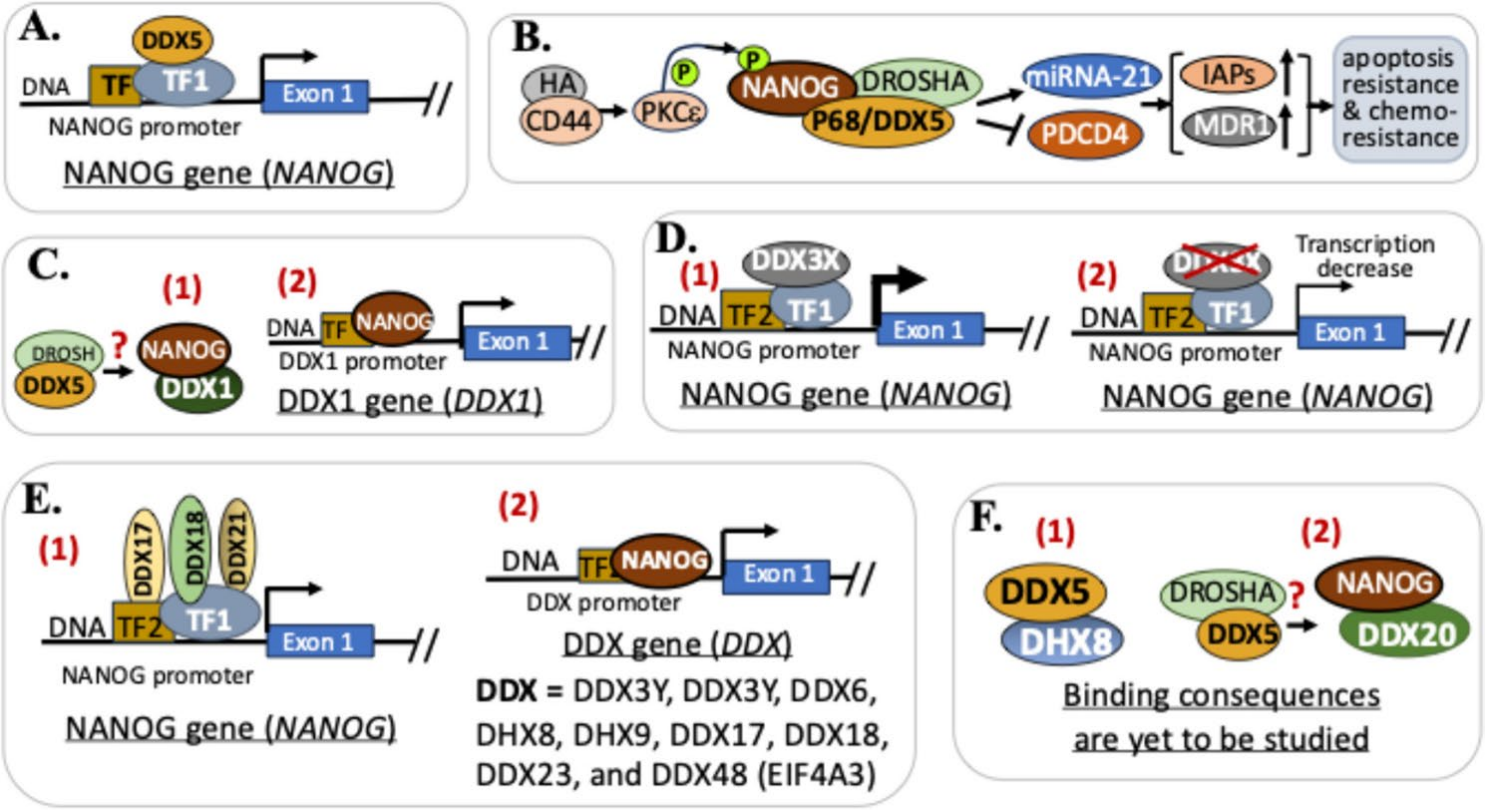
Physical and functional relationships among DDX5, other DDX5-interacting DDX proteins, and NANOG: (**A**) DDX5 binds the NANOG promoter. **B** PKCε-phosphorylated NANOG interacts with the Drosha–DDX5 complex to increase apoptosis resistance and chemotherapy resistance. **C** NANOG interacts with DDX1 (1), and NANOG binds the DDX1 promoter (2). **D** DDX3X binds to and increases NANOG transcription (1); DDX3X ablation decreases NANOG transcription (2). **E** DDX17, DDX18, and DDX21 bind the NANOG promoter (1); in contrast, NANOG binds multiple DDX gene promoters as shown (2). **F** DDX5 binds DHX8 (1), and NANOG binds DDX20 (2); the consequences of these interactions require further investigation

Using CRISPR Affinity Purification in situ of Regulatory Elements (CAPTURE) technology combined with MS analysis to isolate proteins at the NANOG promoter in mouse embryonic stem cells (ESCs), Burramsetty et al. identified DDX5 binding at the NANOG promoter [194] (Fig. 10A), suggesting that DDX5 may regulate NANOG transcription. Furthermore, Bourguignon et al. reported that hyaluronan (HA) binding to CD44 promotes PKCε activation, leading to phosphorylation of NANOG in MCF-7 breast tumor cells. Phosphorylated NANOG then translocates into the nucleus and interacts with the RNase III DROSHA-p68/DDX5 complex [195] (Fig. 10B). This interaction promotes miRNA-21 production and reduces expression of the tumor suppressor PDCD4 [195], resulting in upregulation of IAPs and MDR1 and the acquisition of anti-apoptotic and chemotherapy-resistant phenotypes [195] (Fig. 10B).

We have reviewed physical and functional interactions of DDX5 with DDX1 (Fig. 6A), DDX3X (Fig. 9J), DDX3Y (Fig. 9K), DDX6 (Fig. 9L), DHX9 (Figs. 5D2, 6B, 7 C), DDX17 (Fig. 5C2), DDX18 (Fig. 6C), and DDX21 (Figs. 5D1, 6D, 7B). Building on these findings, additional studies indicate: (i) DDX1 and NANOG are part of the same interactome in ESCs [196] (Fig. 10C1), while NANOG binds the DDX1 promoter in ESCs [197] (Fig. 10C2), suggesting a potential feedback regulatory loop; (ii) DDX3X binds the NANOG promoter (Fig. 10D1), and ablation of DDX3X reduces NANOG expression in PDAC SW1990 cells [198] (Fig. 10D2); and (iii) in ESCs, DDX17, DDX21 [194], and DDX18 [145] bind the NANOG promoter (Fig. 10E1), while NANOG itself binds the promoters of DDX3X, DDX3Y, DDX6, DHX8, DHX9, DDX17, DDX18, DDX23, and DDX48/EIF4A3 [197] (Fig. 10E2*)*. In addition, DDX5 interacts with DHX8 [54] (Fig. 10F1*)*, and NANOG binds the DDX20 promoter [199] (Fig. 10F2).

**Fig. 11:**
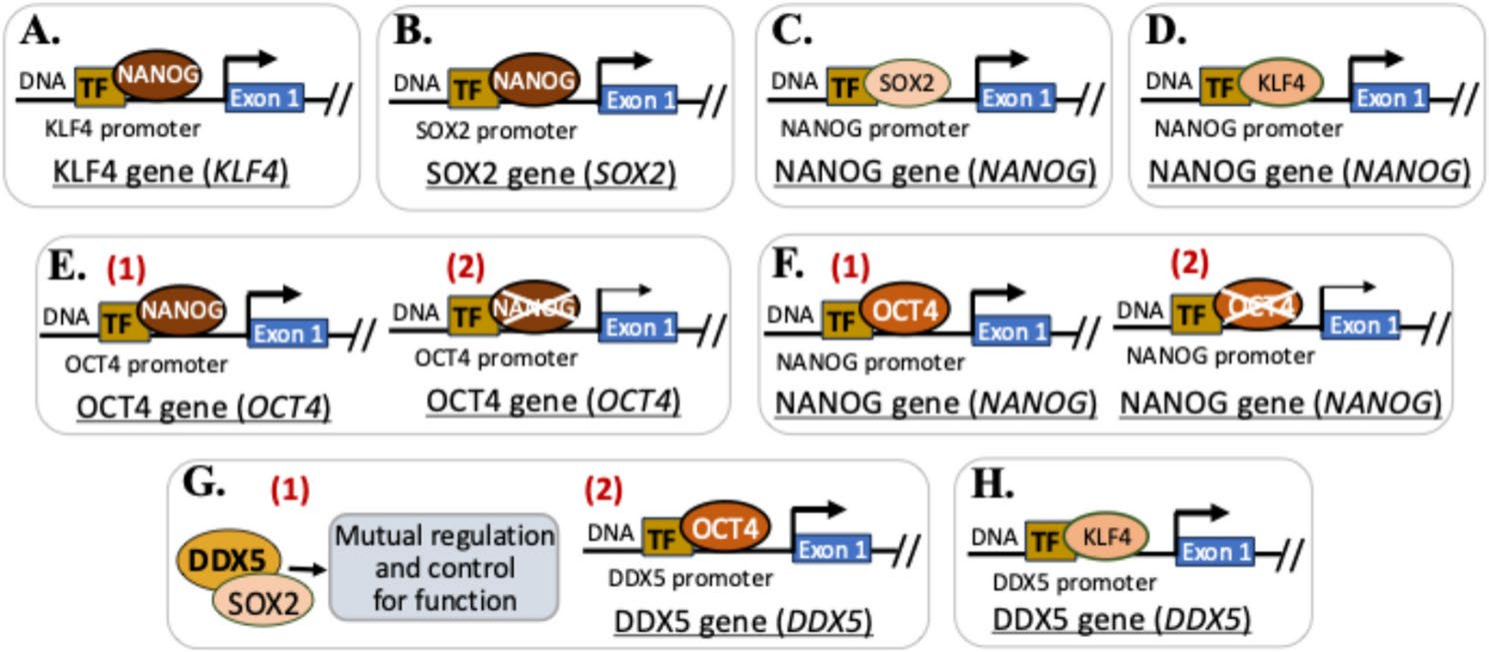
Physical and functional relationships between NANOG and other pluripotency transcription factors: (**A**) NANOG binds the KLF4 promoter. **B** NANOG binds the SOX2 promoter. **C** SOX2 binds the NANOG promoter. **D** KLF4 binds the NANOG promoter. **E** NANOG binds the OCT4 promoter (1), and NANOG ablation decreases OCT4 transcription (2). **F** OCT4 binds the NANOG promoter (1), and OCT4 ablation decreases NANOG transcription (2). **G** DDX5 binds SOX2 ( 1), and OCT4 binds the DDX5 promoter (2). **H** KLF4 binds the DDX5 promoter

Furthermore, NANOG binds the promoters of KLF4 (Fig. 11A) and SOX2 (Fig. 11B), while SOX2 binds the NANOG promoter [197] (Fig. 11C). KLF4 binds the NANOG promoter and increases NANOG expression [200] (Fig. 11D). NANOG binds the OCT4 (Oct3/4) promoter [191, 201] (Fig. 11E1), and ablation of NANOG reduces Oct4 (Pou5f1) expression in PCa 22Rv1 and PC3 cells [202] (Fig. 11E2). Conversely, OCT4 binds the NANOG promoter [203] (Fig. 11F1), and ablation of Oct4 decreases NANOG expression in ESCs [204] in ESCs (Fig. 11F2). This mutual transcriptional regulation among NANOG, KLF4, SOX2, and OCT4 is consistent with evidence that NANOG collaborates with SOX2 and OCT4 to sustain stem-like transcriptional programs, with these factors often co-expressed in PCSCs [191].

NANOG, KLF4, SOX2, and OCT4 are pluripotency TFs frequently co-expressed in PCSC populations and can cooperate to maintain stemness, self-renewal, and tumor-initiating capacity. For example, Oct4, Sox2, and KLF4 cooperate to regulate reprogramming [205]. Importantly, studies in HEK293T and H9 ESCs indicate that NANOG expression and assembly are particularly critical during the late stages of reprogramming, during which NANOG enhances and stabilizes pluripotency promoter/enhancer contacts required for the final activation of pluripotency [206]. In this context, beyond promoting NANOG transcription and interacting with phosphorylated NANOG (Fig. 10A, B), DDX5 has been reported to physically associate with SOX2 [207, 208] (Fig. 11G1) In addition, OCT4 binds the DDX5 promoter [209] and regulates DDX5 transcription in ESCs [210] (Fig. 11G2), and KLF4 regulates DDX5 transcription in LAPC4 PCa cells [211] (Fig. 11H).

Overall, the interplay between DDX5 and pluripotency TFs such as NANOG, SOX2, OCT4, and KLF4 indicates that DDX5 participates in a complex network with these TFs and may influence them through multiple mechanisms. These findings further support the notion that DDX5 could serve as a potent therapeutic target to overcome the heterogeneity and plasticity of advanced PCa, including NEPC, CRPC, and mCRPC.

## DDX5 and UbE2T in inflammation and immune regulation

### DDX5 in inflammatory and immune regulation

Our previous review [1] provided an in-depth examination of the role of DDX5 in immune suppression across four examples [32, 212–214]. Here, we focus on DDX5 physical interactions with, and transcriptional regulation of, immune-associated determinants, highlighting the potential roles of DDX5 and UbE2T in inflammatory and immune modulation.

**Fig. 12:**
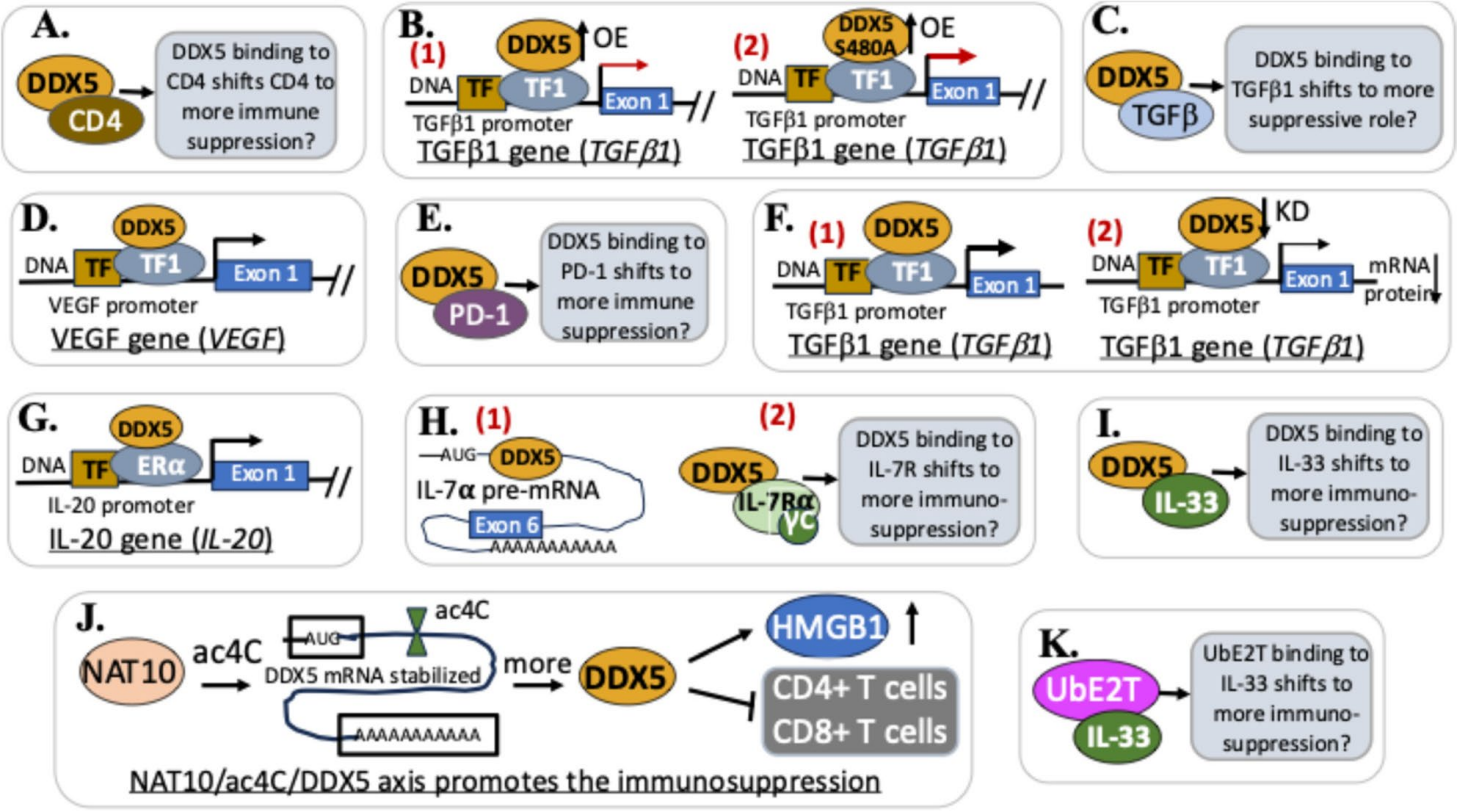
Physical and functional relationships between DDX5 and immune-associated factors: (**A**) DDX5 binds the CD4 protein, potentially regulating distinct functional outcomes. **B** DDX5 binds the TGFβ1 promoter, and DDX5 overexpression (OE) reduces TGFβ1 expression (1); in contrast, the DDX5 S480A polymorphism binds the TGFβ1 promoter, and OE of DDX5 S480A increases TGFβ1 expression (2). **C** DDX5 binds the TGFβ protein. **D** DDX5 binds the VEGF promoter. **E** DDX5 binds the PD-1 protein. **F** DDX5 binds the TGFβ1 promoter (1), and DDX5 knockdown (KD) decreases TGFβ1 transcription (2). **G** DDX5 together with ERα binds the IL-20 promoter. **H** DDX5 binds IL-7α pre-mRNA (1), and DDX5 binds the IL-7 receptor (2). **I** DDX5 binds IL-33. **J** NAT10 modifies DDX5 mRNA with ac4C to stabilize DDX5 mRNA, increasing transcription/translation of DDX5 and DDX5-associated proteins; DDX5 together with other ac4C-modified proteins increases high mobility group box 1 (HMGB1) expression and blocks CD4 + and CD8 + T-cell responses. **K** UbE2T binds IL-33

To our knowledge, there are no reports indicating that DDX5 directly regulates the expression of, or binds to, major immune-activating cytokines and growth factors (e.g., IFN-γ, IL-2, IL-12, IL-15, GM-CSF), costimulatory and immune-activating cell surface receptors (e.g., CD80/B7-1, CD86/B7-2, CD40, CD40L, 4-1BB/CD137, 4-1BBL, ICOS, ICOSL), or immune cell–recruiting chemokines (e.g., CXCL9, CXCL10, CXCL11, CCL5/RANTES). However, prior work reported that the CD4 molecule on CD4⁺ T cells interacts with p68/DDX5 [215] (Fig. 12A). This finding is intriguing because CD4 expression can exert either immune-activating or immune-suppressive roles depending on context, locally within the tumor microenvironment (TME) or at regional/distal sites such as lymphoid tissues. For example, upon engagement with antigen-presenting cells - including activated B cells, macrophages, and dendritic cells - CD4⁺ T cells provide essential “help” via cytokines and costimulatory signals to activate CD8⁺ T cells. Conversely, a subset of CD4⁺ T cells (regulatory T cells; Tregs) expressing FoxP3 and CD25 can suppress CD8⁺ T cell responses.

In contrast, DDX5 promotes the expression of and/or directly interacts with multiple key immune-suppressive factors in the TME. It has been shown that DDX5 and its polymorphic variant DDX5 S480A bind the TGFβ1 promoter. Notably, cellular context appears to be critical, as overexpression (OE) of DDX5 (Fig. 12B1) and DDX5 S480A (Fig. 12B2) exerted opposing effects on TGFβ1 expression in hepatic stellate and LX-2 cells [216] (Fig. 12B). Additional studies reported that: (a) DDX5 binds to TGFβ in MDA-MB-231 and MCF10A cells [217] (Fig. 12C); (b) DDX5 binds to the VEGF gene promoter in HCT116 and SW480 colorectal cancer (CRC) cells [218] (Fig. 12D); and (c) DDX5 binds to PD-1 [219] (Fig. 12E).

Furthermore, several interleukins (ILs) and their cognate receptors - which play distinct roles in shaping immune activation versus suppression in cancer - were found to be regulated or bound by DDX5. These include: (a) DDX5 binds the IL-1β promoter in U251, U87, and LN229 glioblastoma cells (Fig. 12F1), and knockdown of DDX5 decreases IL-1β mRNA and protein levels [212] (Fig. 12F2). This is notable because IL-1β can contribute to immune activation (e.g., enhancing dendritic cell maturation and T cell activation) and, in a context-dependent manner, immune suppression (e.g., driving tumor-promoting inflammatory consequences in certain tumors). Chronic IL1β-driven inflammation may promote recruitment of immunosuppressive myeloid cells, such as myeloid-derived suppressor cells (MDSCs) and tumor-associated macrophages (TAMs); (b) ERα and DDX5 co-occupy the IL-20 promoter to activate IL-20 gene expression [105] (Fig. 12G); (c) DDX5 binds both IL-7Rα pre-mRNA (Fig. 12H1) and IL-7R protein (Fig. 12H2) in HeLa cells [220]; and (d) DDX5 binds IL-33 in HUH7 liver cancer cells [221] (Fig. 12I). Notably, IL-33 can also mediate both immune activation (e.g., promoting CD8⁺ T cell and NK cell responses in certain tumor models) and context-dependent immune suppression (e.g., recruiting and activating Tregs and M2-like macrophages).

Finally, a recent study revealed that N-acetyltransferase 10 (NAT10)-mediated N4-acetylcytidine (ac4C) modification of DDX5 mRNA (as well as CEBPG and HLTF mRNAs) enhances their stability and translation efficiency. This NAT10/ac4C/DDX5 axis upregulates HMGB1 and suppresses CD4⁺ and CD8⁺ T cell activity [2] (Fig. 12J), resulting an overall immune suppressive state.

Collectively, these findings indicate that DDX5 contributes to immune suppression in the TME, and that DDX5 inhibition could both block downstream oncogenic signaling and reinvigorate antitumor immunity.

### UbE2T in inflammation and immune regulation

In contrast to the diverse DDX5-mediated activities within the immune system (Fig. 12A-J), UbE2T has not been reported to bind major immune-activating cytokines or growth factors, immune-activating cell surface receptors, or immune cell–recruiting chemokines. However, similar to DDX5, UbE2T can bind IL-33 in HUH7 liver cancer cells [221] (Fig. 12K). This interaction may be functionally relevant, given that IL-33 can mediate both immune activation and context-dependent immune suppression. It is therefore possible that interactions among IL-33, DDX5, and UbE2T may cooperatively facilitate or enhance IL-33–driven immunosuppressive effects in certain tumors.

Notably, three studies published in 2022 further revealed correlations between UbE2T expression and immune cell profiles within the TME. Bioinformatic analyses of available lung adenocarcinoma (LUAD) datasets showed that UbE2T-related cell cycle gene (UrCCG) subtypes were associated with distinct immune-TME characteristics [222]. Specifically, analysis of immune infiltrates, immune checkpoints, and immunogenic cell death identified four subgroups (G1-G4) with different immune landscapes. Tumors in G3 and G4 were immunologically “hot” and tended to respond to immunotherapy compared with G2 subtypes. Importantly, these UrCCG-based LUAD cohorts (G1-G4) also displayed significant differences in survival, molecular features, immune infiltrates, and immunotherapy responses [222].

In contrast, a separate study using a breast cancer database reported that UbE2T shifts the CD4⁺ Th1/Th2 balance toward Th2 dominance in basal and luminal-B breast cancer subtypes, which correlates with poorer prognosis [223]. Collectively, these findings suggest that the immunomodulatory consequences of UbE2T activity are likely cancer type dependent.

Interestingly, bioinformatic analyses of 516 LUAD samples and 59 normal samples from TCGA identified nine survival-related differentially expressed genes (DEGs) in LUAD, including ASF1B, CA9, CCNB2, CCNE1, RRM2, SAPCD2, TCN1, TPX2, and UbE2T [224]. Given the high expression of UbE2T in LUAD, the study further examined immune associations and found that UbE2T expression positively correlates with γδ T cells, T cell follicular helper (Tfh) cells, activated CD4⁺ T cells, activated memory NK cells, macrophage M0, and naïve B cells. In contrast, low UbE2T expression correlates with resting CD4⁺ T cells, resting myeloid dendritic cells, activated mast cells, M2-like macrophages, and plasma B cells [224]. Importantly, these correlations do not imply causation in the reported model systems. Moreover, the biological consequences of UbE2T expression may be highly dependent on cancer type and context, consistent with prior observations showing differing immunologic associations [223]. Nevertheless, such inconsistency needs to be further investigated.

In addition, beyond the shared interaction of DDX5 and UbE2T with IL-33, DDX5 and UbE2T have been reported to be mutually associated within the same protein complex during DNA repair processes, which could indirectly influence immune responses - particularly in the context of cancer therapies targeting DDX5 and UbE2T as dual promoters of tumor progression and treatment resistance.

## DDX5 and UbE2T are FL118 therapeutic targets

### DDX5 and UbE2T are physical and functional targets for FL118

#### DDX5 and UbE2T are physical targets of FL118

**Fig. 13:**
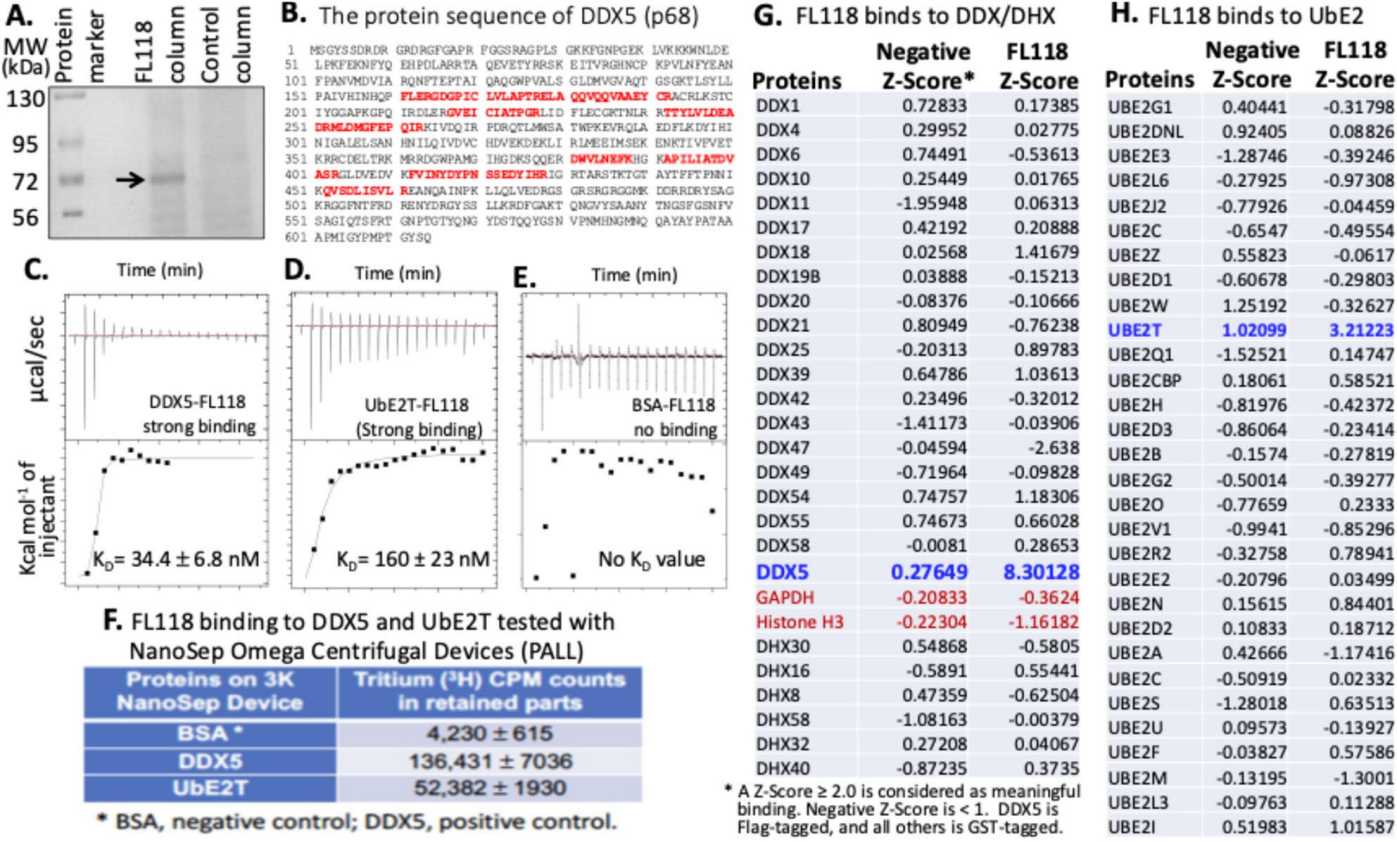
FL118 binds DDX5 and UbE2T with high affinity and selectivity: (**A**) FL118 affinity-column purification identified a ~ 70 kDa protein. FL118 was directly coupled to a beaded resin. SW620 protein lysates were purified on the FL118 affinity column through stringent washing, elution with 8 M urea, de-urea, and sample concentration to 20–30 µL, followed by separation on a 5–20% SDS–PAGE gel. **B** The ~ 70 kDa protein band in (**A**) was analyzed by mass spectrometry (MS). The band was digested in-gel, and ten peptides were isolated and used to search protein databases. The 10 peptides fully matched the DDX5 (p68) protein sequence. **C-E** Representative isothermal titration calorimetry (ITC) results. KD values shown in the figure are mean ± standard deviation (SD) from two ITC analyses. Purified Flag-tagged DDX5 (Flag-DDX5) (**C**), Flag-UbE2T (**D**), and BSA (**E**) were loaded into a 96 DeepWell PP plate, and FL118 (**C**-**E**) was automatically titrated stepwise into the protein cell by 20 injections over 60 min (one injection per 3 min) on a MicroCal–Malvern Auto-iTC200. **F **Interaction of tritium (³H)-labeled FL118 (³H-FL118) with bovine serum albumin (BSA), DDX5, and UbE2T tested using NanoSep Omega centrifugal devices (Pall Life Sciences). **G**,** H** Protein microarray studies indicated that FL118 selectively binds DDX5 (Z-score: 8.3) and UbE2T (Z-score: 3.2) but does not bind other DDX/DHX proteins (**G**) or UbE2 family proteins (**H**). Z-score is the protein-binding tritium (³H) signal minus the mean signal from all proteins tested on the microarray, divided by the standard deviation (SD) across all proteins. A Z-score > 2.0 indicates a signal > 2 × SD above the median signal from all proteins on the microarray. Negative Z-scores are < 1. Z-scores of GAPDH and histone H3 are negative controls. On this microarray, DDX5 was Flag-tagged, whereas all other proteins were GST-tagged. This figure is adapted from our previous publication [41] with additional new data

Through high-throughput screening of compound libraries using survivin as a target and biomarker, followed by a series of in vitro and in vivo hit-to-lead selection steps, FL118 was identified as the top candidate for further development [159]. We recently demonstrated that FL118 is a molecular glue degrader and can bind the oncoprotein DDX5 (p68) [41]. Specifically, affinity column purification using FL118 as a bait identified an unknown ~ 70 kDa protein that was present in the FL118 column but absent in the control column (Fig. 13A). Mass spectrometry (MS) analysis identified this protein as DDX5 (Fig. 13B). In parallel, use of tritium (^3^H)-labeled FL118 (^3^H-FL118) as a probe for a genome-wide screening of > 9,400 human proteins identified the ubiquitin-conjugating enzyme E2T (UbE2T) as a top binding candidate. Consistently, isothermal titration calorimetry (ITC) studies demonstrated that FL118 binds DDX5 (K_D_ = 34 nM) and UbE2T (K_D_ = 160 nM) with high affinity (Fig. 13C, D), whereas no detectable binding was observed with the control protein BSA (Fig. 13E). Binding of FL118 to DDX5 and UbE2T was further validated using NanoSep Omega centrifugal devices with ^3^H-FL118 as a tracer (Fig. 13F). Importantly, FL118 binding to DDX5 and UbE2T was highly selective, showing no meaningful affinity for other DDX/DHX proteins (Fig. 13G) or other UbE2 proteins (Fig. 13H).

### Physical binding of DDX5 or UbE2T by FL118 reduces their expression

**Fig. 14:**
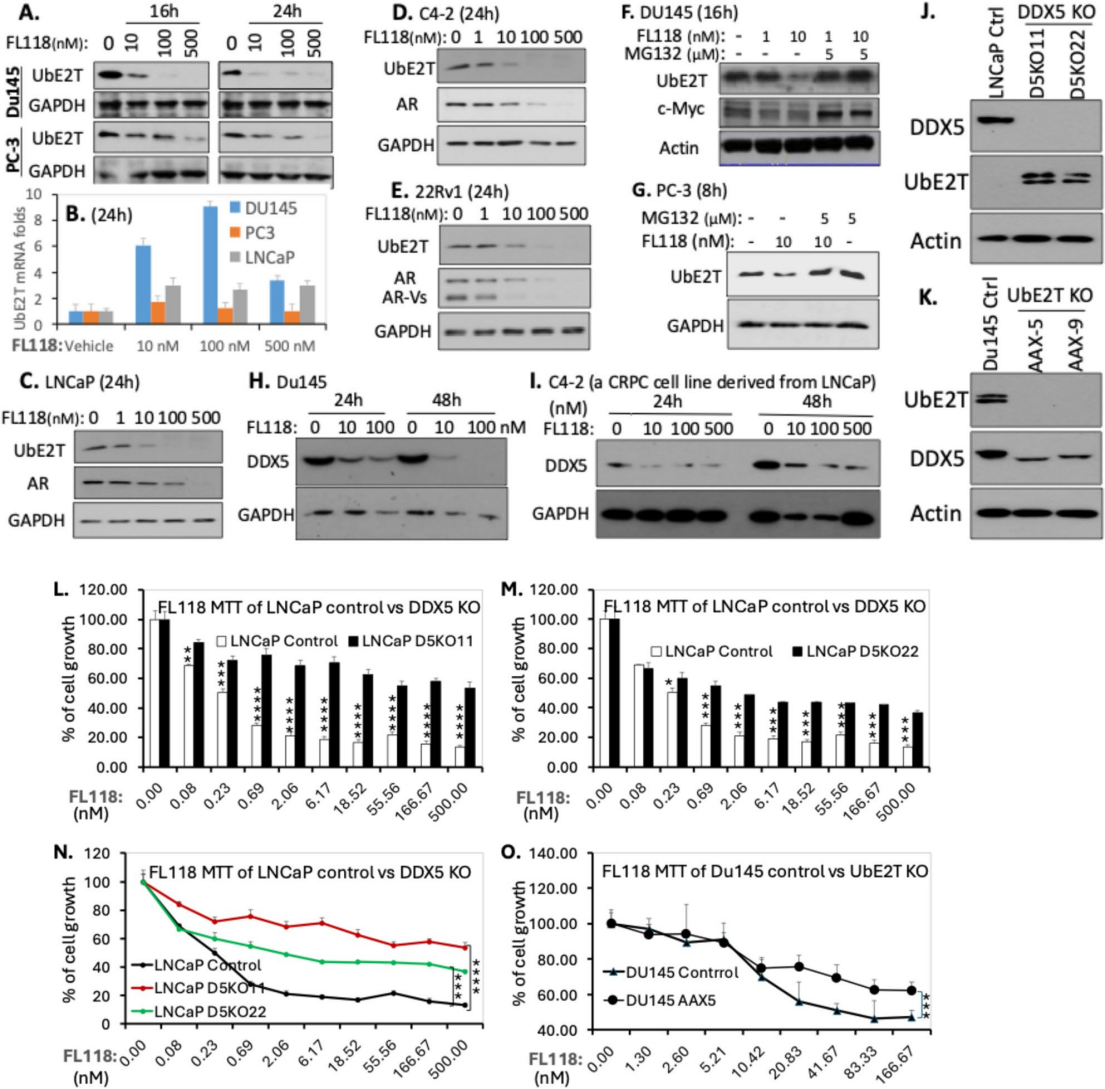
A-K. FL118 suppresses UbE2T and DDX5 protein in a p53-independent manner and reveals reciprocal regulation between UbE2T and DDX5: (**A**-**E**) FL118 destabilizes UbE2T protein (**A**, **C**–**E**) without inhibiting UbE2T mRNA (**B**). PCa cells were treated with or without FL118 as indicated, lysed, and analyzed for UbE2T protein (**A**) or UbE2T and androgen receptor (AR) (**C**–**E**) by Western blot (WB), and for UbE2T mRNA by real-time RT–PCR (**B**). **F,G** The proteasome inhibitor MG132 blocks FL118-mediated UbE2T protein degradation. **F** DU145 cells were treated with or without FL118 in the presence or absence of MG132 for 16 h, and UbE2T and c-Myc were analyzed by WB. **G** PC-3 cells were treated with or without FL118 and/or MG132 as indicated. (**H**, **I**) FL118 reduces DDX5 protein in PCa DU145 (**H**) and C4-2 (**I**) cells; UbE2T and DDX5 were determined by WB. **J** DDX5 knockout (KO) in LNCaP cells induces UbE2T expression (DDX5 KO clones D5KO11 and D5KO22). **K** UbE2T KO in DU145 cells partially reduces DDX5 expression (UbE2T KO clones AAX-5 and AAX-9). DDX5 and UbE2T expression in (**J**, **K**) was determined by WB. GAPDH in (**A**, **C**-**E**, **G**-**I**) and actin in (**F**, **J**, **K**) served as internal controls for total protein loading. Each bar in (**B**) represents mean + SD from 3 independent analyses. This figure presents newly generated data not previously published. L-O. PCa cells with knockout (KO) of either DDX5 or UbE2T exhibit resistance to FL118-mediated cell growth inhibition: (**L**, **M**) DDX5 KO in PCa LNCaP cells results in marked loss of FL118 activity in inhibiting cell growth/viability. LNCaP DDX5 KO clone #11 (LNCaP D5KO11) cells (**L**) and LNCaP DDX5 KO clone #22 (LNCaP D5KO22) cells (**M**), in parallel with LNCaP control cells, were treated with FL118 as indicated. Cell viability was determined by MTT assay 72 h after treatment. Each bar represents the mean + SD from 3 independent assays. **N** Results from (**L**) and (**M**) are combined and presented in curve format. **O** UbE2T KO in PCa Du145 cells results in loss of FL118 activity in inhibiting cell growth/viability. Du145 UbE2T KO clone AAX5 (Du145 AAX5) cells, in parallel with Du145 control cells, were treated with FL118 as indicated. Cell viability was determined by MTT assay 72 h after treatment. Each point in each curve represents the mean + SD from 3 independent assays. *p < 0.05; **p < 0.01; ***p < 0.001; ****p < 0.0001

Consistent with previous findings that FL118 binding to DDX5 rapidly induces DDX5 dephosphorylation (≤ 6 h), followed by DDX5 protein degradation in colorectal cancer (CRC) and pancreatic ductal adenocarcinoma (PDAC) [41], our studies in prostate cancer (PCa) models revealed that FL118 treatment of Du145 and PC-3 cells rapidly reduces UbE2T protein levels (Fig. 14A) without a corresponding decrease in UbE2T mRNA (Fig. 14B). Notably, the observed increase in UbE2T mRNA is likely a consequence of FL118-induced cell death signaling, which triggers a compensatory feedback response aimed at promoting cell survival.

Furthermore, FL118-mediated reduction of UbE2T protein in LNCaP (Fig. 14C) and C4-2 (Fig. 14D) cells is accompanied by inhibition of the androgen receptor (AR) (Fig. 14C, D). Notably, in 22Rv1 cells, FL118 treatment rapidly decreased not only AR but also AR variants (AR-Vs) (Fig. 14E). Development of AR and AR-V degraders has been an intense area of research for decades, and substantial efforts continue [225–227], although several challenges remain to be resolved [228]. Nevertheless, agents that exclusively target AR/AR-Vs may not effectively overcome lethal PCa resistance. The development of galeterone and its analogs as AR/AR-V-degrading agents provides a representative example [229–232]. Therefore, there remains a clear need for agents capable of broader therapeutic effects beyond suppression of AR/AR-V expression alone.

To further elucidate the mechanism underlying FL118-mediated reduction of UbE2T protein, Du145 and PC-3 cells were treated with FL118 in the presence and absence of the proteasome inhibitor MG132. MG132 treatment rescued the FL118-induced reduction of UbE2T protein (Fig. 14F, G), indicating that FL118-mediated degradation of UbE2T occurs through the proteasome pathway, similar to FL118-mediated degradation of DDX5 [41]. In addition, consistent with the finding that FL118 degrades DDX5 in CRC and PDAC cells [41], FL118 effectively inhibits DDX5 expression in PCa cells as well (Fig. 14H, I). Furthermore, knockout (KO) of DDX5 in LNCaP cells increased UbE2T expression (Fig. 14J), whereas KO of UbE2T partially decreased DDX5 expression (Fig. 14K). These observations suggest that simultaneous degradation of both DDX5 and UbE2T is required to more effectively conquer human cancers.

To provide additional evidence that FL118 directly targeting of DDX5 and UbE2T is essential for its high anticancer efficacy, we took advantage of the available reagents used in Fig. 14J, K and evaluated FL118-mediated inhibition of cell growth. We found that prostate cancer (PCa) cells with knockout of either DDX5 or UbE2T exhibited resistance to FL118-mediated growth inhibition (Fig. 14L-O), consistent with the behavior previously observed in pancreatic cancer cells following FL118 treatment [41].

Nevertheless, we would like to note that FL118 is a small molecule, and highly efficacious small-molecule anticancer agents may have multiple protein targets. For example, the blockbuster anticancer drug lenalidomide (Revlimid) has been reported to act through multiple neosubstrates/protein targets [233]. Given the exceptional efficacy of FL118 against difficult-to-treat cancers, it remains possible that future studies will identify additional direct FL118 targets. If so, this would not be unexpected.

Additionally, regarding FL118 acting as a dual molecular glue degrader, although FL118 shows strong functional and biochemical features consistent with a dual molecular glue degrader, definitive biochemical and structural demonstration of the relevant E3 ligase-mediated ternary complex formation remains an important objective of future investigation.

### Genetic modulation of DDX5 or UbE2T affects downstream target expression

**Fig. 15:**
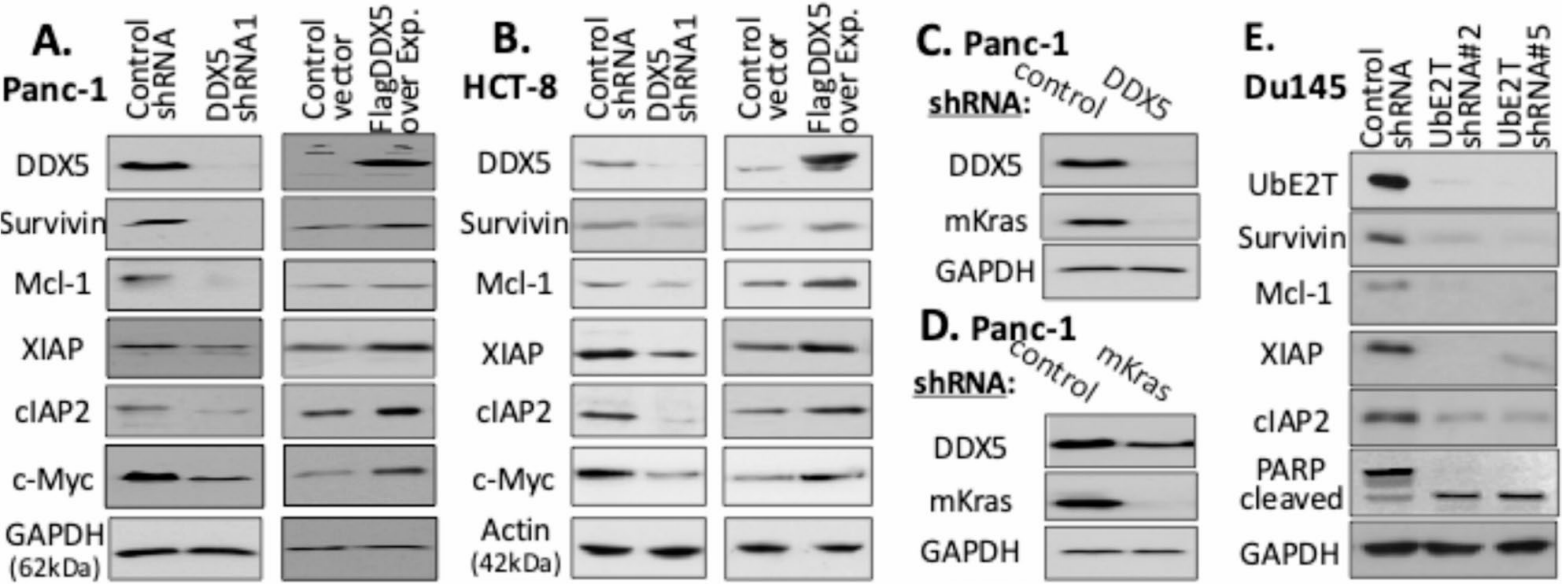
Genetic modulation of DDX5 or UbE2T in cancer cells alters FL118-inhibited proteins: (**A**,** B; **left panels) shRNA silencing of DDX5 decreases survivin, Mcl-1, XIAP, cIAP2, and c-Myc. Cells were infected with control shRNA or DDX5 shRNA lentiviral particles as indicated, lysed 48 h post-infection, and analyzed by WB for DDX5 and the indicated proteins. **A**,** B; **right panel**s** Forced expression of Flag-DDX5 increases survivin, Mcl-1, XIAP, cIAP2, and c-Myc. Cells were transfected with empty vector (control) or Flag-DDX5 expression vector (RC200371, OriGene) using Lipofectamine 2000, lysed 48 h post-transfection, and analyzed by WB. Overexpressed Flag-DDX5 was detected by Flag antibody in (A) (endogenous DDX5 was not detectable in this condition; A, right panel). Overexpressed Flag-DDX5 was detected by DDX5 antibody in (**B**); Flag-DDX5 is slightly larger than endogenous DDX5 (B, right panel). **C** DDX5 knockdown abrogates mutant KRAS (mKRAS). Panc-1 cells were infected with control shRNA or DDX5 shRNA lentivirus and analyzed 48 h post-infection for DDX5 and mKRAS by WB. **D** mKRAS knockdown partially decreases DDX5. Panc-1 cells were infected with control shRNA or mKRAS shRNA lentivirus and analyzed 48 h post-infection for DDX5 and mKRAS by WB. **E** Lentiviral shRNA silencing of UbE2T decreases survivin, Mcl-1, XIAP, and cIAP2 in parallel with apoptosis (cleaved PARP). DU145 cells were infected with control shRNA or UbE2T shRNA (#2, #5); after puromycin selection, cells were analyzed by WB for the indicated proteins and PARP cleavage. GAPDH in (**A**, **C**-**E**) and actin in (**B**) served as loading controls. This figure is adapted from our previous publication [41] with the addition of new data

Consistent with our earlier findings that FL118 treatment of CRC and PC-3 cells inhibits expression of survivin, Mcl-1, XIAP, and cIAP2 [159], our genetic modification studies [41] further demonstrated that shRNA-mediated knockdown (KD) of DDX5 in PDAC Panc-1 or CRC HCT-8 cells reduced expression of survivin, Mcl-1, XIAP, cIAP2, and c-Myc (Fig. 15A, B, left panels). In contrast, overexpression (OE) of DDX5 increased expression of these proteins (Fig. 15A, B, right panels), indicating that survivin, Mcl-1, XIAP, cIAP2, and c-Myc are downstream targets of DDX5.

Given the importance of mutant Kras (mKras) in PDAC, we performed mutual KD of DDX5 and mKras. We found that shRNA silencing of DDX5 resulted in abrogation of mKras expression (Fig. 15C), whereas shRNA silencing of mKras partially decreased DDX5 expression (Fig. 15D). These results indicate that mKras functions as a downstream target of DDX5, although mKras may exert partial regulatory influence on DDX5 expression. Furthermore, similar to the effects observed with DDX5 KD, shRNA-mediated silencing of UbE2T downregulated survivin, Mcl-1, XIAP, and cIAP2, accompanied by PARP cleavage, a hallmark of apoptosis (Fig. 15E).

### New studies indicate that FL118 has great potential to conquer advanced PCa

**Fig. 16:**
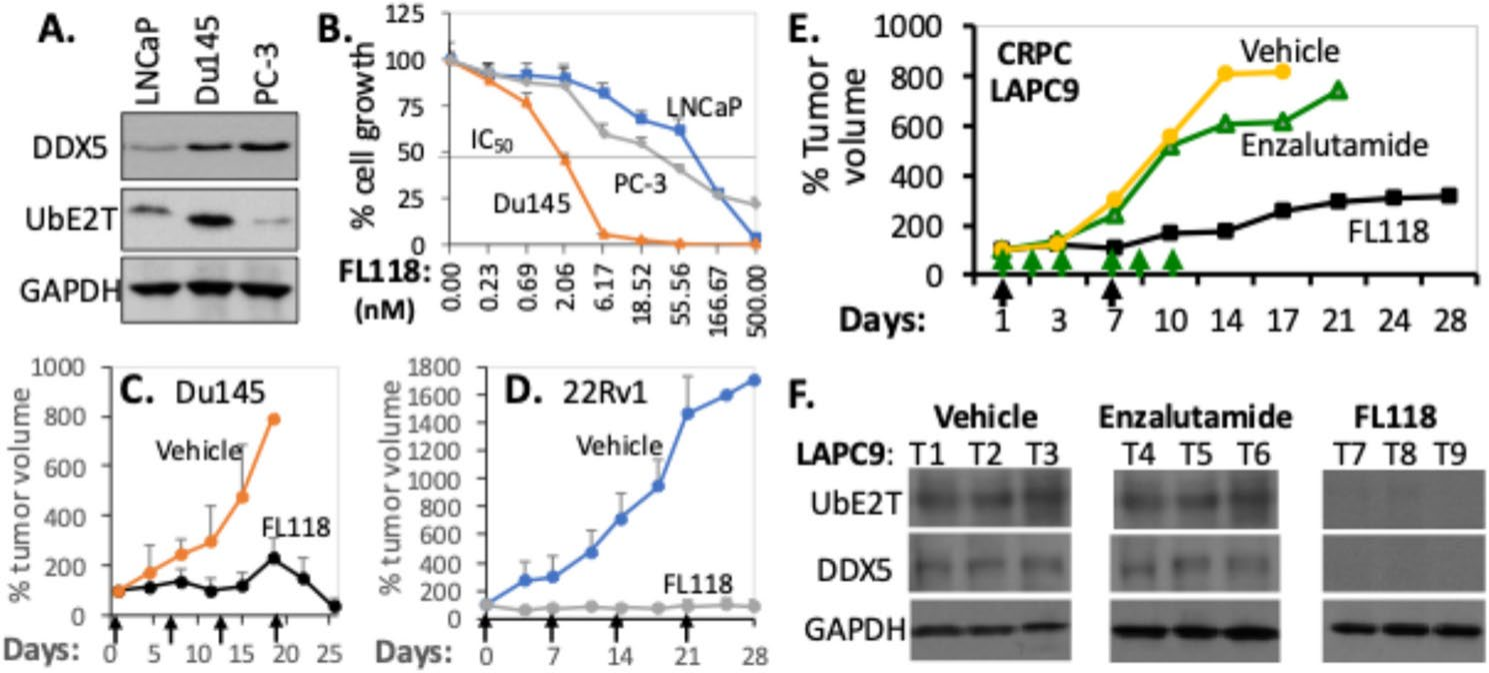
DDX5/UbE2T expression and FL118 antitumor efficacy in prostate cancer (PCa) models: (**A**) WB analyses of DDX5 and UbE2T in three commonly used PCa cell lines; GAPDH is a loading control. **B** Growth inhibition of LNCaP, DU145, and PC-3 cells in response to FL118. Cells in 96-well plates were treated with or without FL118 at indicated concentrations for 72 h, and cell growth/viability was analyzed by MTT. Curves represent mean + SD from 3 independent assays. **C** Antitumor efficacy of FL118 in DU145 xenografts. Severe combined immunodeficiency (SCID) mice were orally treated with vehicle or FL118 (5 mg/kg, weekly ×4; arrows). **D** Antitumor efficacy of FL118 in 22Rv1 CRPC tumors. SCID mice were orally treated with vehicle or FL118 (5 mg/kg; arrows). Tumor model setup in (**C**-**E**): xenograft tumors were inoculated into SCID mice at the flank from tumor-maintenance mice. Treatment began when tumors reached 150–200 mm³. **E** Antitumor efficacy of FL118 and enzalutamide in CRPC LAPC9 tumors. Mice were treated with enzalutamide (10 mg/kg, intraperitoneally, three times per week for 2 weeks; green arrows) or vehicle/FL118 (5 mg/kg, orally, once per week for 2 weeks; black arrows). Tumor size variation is within 10%. **F** In vivo FL118 target analysis of CRPC LAPC9 tumors collected at study end in (**E**). Three LAPC9 tumors (T) per group were analyzed by WB. This figure is adapted from our previous publication [236], with additional new data in (**A**, **B**, **C**, **D**, **F**)

We and others have demonstrated a critical role for DDX5 in mediating the antitumor effects of FL118 [41, 234], and FL118 has been shown to effectively eliminate human CRC and PDAC tumors in animal models [41]. Based on the findings summarized in Figs. 13, 14 and 15, we performed both in vitro and in vivo studies to evaluate FL118 efficacy in PCa. Consistent with high expression of both DDX5 and UbE2T in Du145 cells (Fig. 16A), Du145 cells exhibited the greatest sensitivity to FL118-mediated inhibition of cell growth and viability (Fig. 16B). In agreement with this observation, Du145 cells were also the only PCa cell line able to form xenograft tumors without addition of 50% Matrigel, and FL118 treatment effectively suppressed growth of these tumors (Fig. 16C).

We further evaluated FL118 efficacy using 22Rv1 cell-derived xenograft tumors which expresses both AR and the AR-7 variant; both can be effectively inhibited by FL118 (Fig. 14E). FL118 exhibited excellent efficacy against 22Rv1 tumor growth (Fig. 16D). Furthermore, we used the co-author Dr. Dean Tang’s laboratory-established CRPC LAPC9 xenograft tumor model [235], which is AR low/negative (AR^lo/−^) and androgen-insensitive and resistant to enzalutamide treatment (enzalutamide-nonresponsive), and tested FL118’s efficacy against this CRPC tumor model (Fig. 16E). While enzalutamide administered at its maximum tolerated dose (MTD; 10 mg/kg) for six intraperitoneal treatments over two weeks (Fig. 16E, green arrows) produced minimal tumor inhibition, FL118 at half the MTD (5 mg/kg) with only two treatments (Fig. 16E, black arrows) significantly inhibited LAPC9 tumor growth (Fig. 16E). To further investigate the underlying mechanism, we isolated three LAPC9 tumors from each treatment group shown in Fig. 16E and performed western blot (WB) analysis. Whereas enzalutamide did not reduce UbE2T or DDX5 expression, FL118 effectively reduced DDX5 and UbE2T protein levels (Fig. 16F).

### High DDX5 and/or UbE2T expression is associated with clinically aggressive PCa

**Fig. 17:**
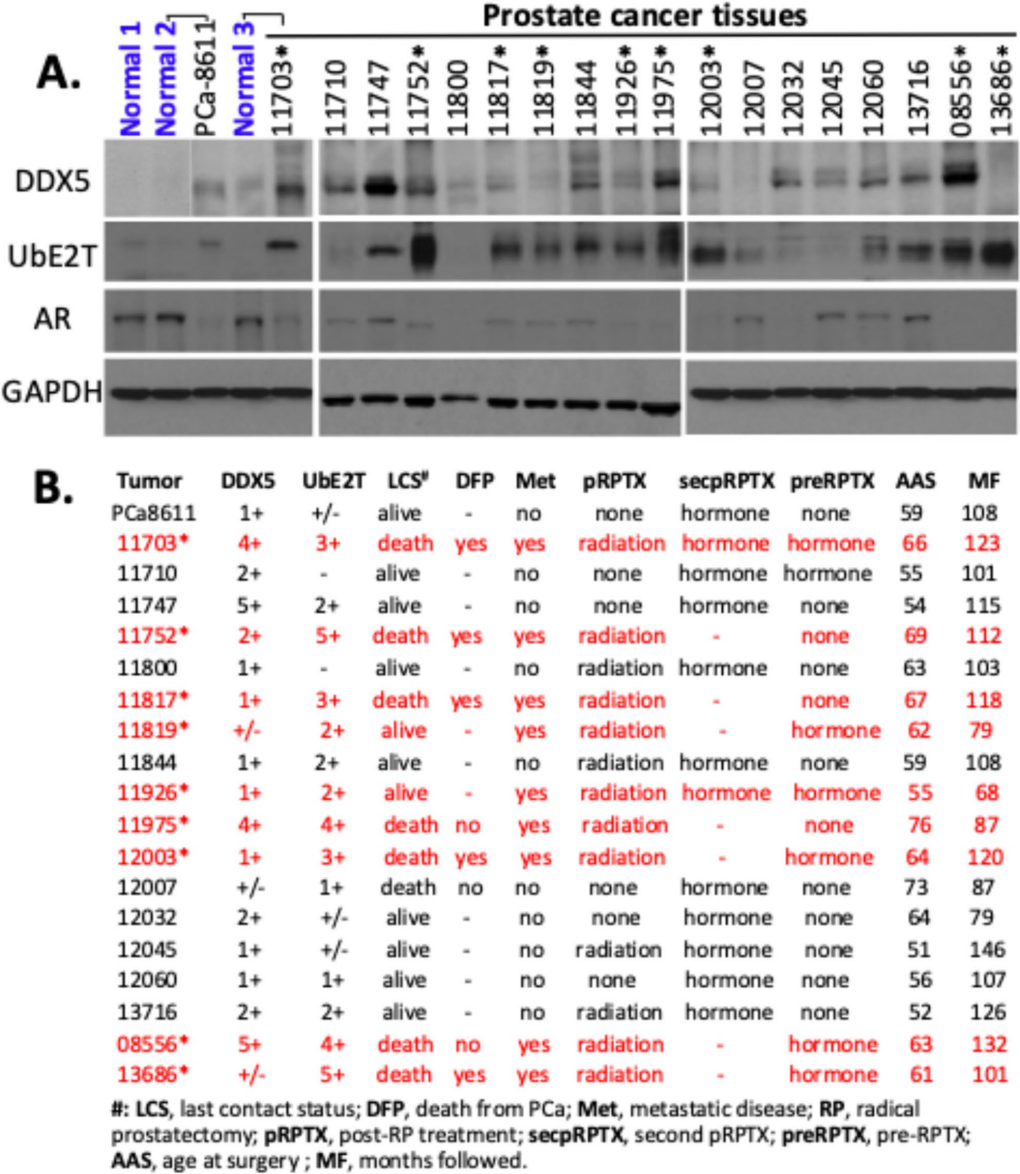
Clinical relevance of DDX5 and/or UbE2T as biomarkers and therapeutic targets in advanced PCa: **A** DDX5, UbE2T, and AR expression in normal and PCa tissues. Thirty µg of protein lysates from normal prostate tissues (Normal 1, no matched tumor; Normal 2 matched to PCa-8611; Normal 3 matched to 11703) and PCa specimens were analyzed by WB for USP2a, UbE2T, and AR; GAPDH served as a loading control. PCa tissues marked with “*” were obtained from metastatic sites or from primary sites with metastatic disease. **B** Summary of clinical information for the patients corresponding to the 19 PCa tissues. Full names of abbreviations used in (**B**) are provided at the bottom of (**B**). This figure presents new and unpublished data

The in vitro and in vivo findings shown in Figs. 13, 14, 15 and 16 are highly relevant to PCa status in the clinic. DDX5/p68 is overexpressed in PCa tissues compared with benign tissues [237], and UbE2T expression is elevated in PCa, particularly in cases with distant metastasis [238]. Consistent with these findings, analysis of clinically normal prostate tissues and PCa tissues revealed that most PCa samples highly express DDX5 and/or UbE2T proteins (Fig. 17A), whereas normal prostate tissues exhibit little to no expression of these proteins (Fig. 17A). Notably, DDX5 and UbE2T expression in PCa tissues is associated with low androgen receptor (AR) levels (Fig. 17A).

Significantly, nine PCa tissues collected from metastatic sites or from primary tumors with metastatic disease exhibited high expression of DDX5 and/or UbE2T, accompanied by absent or low AR expression (Fig. 17A, marked with “*”). Key clinical information for the 19 PCa tumor tissues, together with the intensity of DDX5 and UbE2T expression (−, +/−, 1+, 2+, 3+, 4+, 5+), is presented in Fig. 17B. Statistical analysis using the Wilcoxon-Mann-Whitney test revealed that DDX5 and UbE2T expression in metastatic PCa tissues is significantly higher than that in non-metastatic tissues. These findings further support the potential of DDX5 and UbE2T as biomarkers and therapeutic targets in lethal forms of PCa, including CRPC, mCRPC, and NEPC.

Considering these new PCa data together with prior evidence of FL118 potent antitumor activity in CRC and PDAC tumors [41], the most promising indications for FL118 may include advanced PDAC, CRC, and PCa, particularly given the recent finding that clinically and orally compatible formulation-manufactured FL118 products exhibit high efficacy with low toxicity [236].

### DDX5 and UbE2T as targets for advanced cancer therapeutics

We have reviewed the roles of the FL118 targets DDX5 and UbE2T in regulating EZH2, NANOG, and prostate cancer stem cells (PCSCs) in advanced prostate cancer (PCa), neuroendocrine prostate cancer (NEPC), CRPC, and mCRPC, which represent some of the most difficult-to-treat malignancies. In this section, we extend our analysis to six additional challenging cancers, including advanced colorectal cancer (CRC), pancreatic ductal adenocarcinoma (PDAC), osteosarcoma, Ewing sarcoma, ovarian cancer, and glioma/glioblastoma.

### DDX5 and UbE2T in advanced CRC

#### DDX5 in CRC

Advanced CRC remains a difficult-to-treat and largely incurable disease due to limited therapeutic options and high genetic heterogeneity, including frequent mutations in *KRAS*, *NRAS*, *BRAF*, and *TP53*, among others. Advanced CRC is often resistant to chemotherapy, targeted therapies, and immunotherapies. Previous studies have demonstrated that: (i) high DDX5 expression is associated with aggressive CRC and poor prognosis [239]; (ii) the long noncoding RNA nuclear-enriched abundant transcript 1 (NEAT1) directly binds to and stabilizes DDX5, thereby activating Wnt/β-catenin signaling to promote CRC cell proliferation, migration, and invasion, and clinically, co-expression of NEAT1 and DDX5 correlates with reduced overall and disease-free survival in CRC patients [240]; (iii) the p68/DDX5/β-catenin axis activates RelA and NF-κB signaling, driving colon carcinogenesis [241]; and (iv) O-linked N-acetylglucosamine (GlcNAc) transferase (OGT) directly interacts with and stabilizes DDX5 via O-GlcNAcylation, activating AKT/mTOR signaling and accelerating CRC progression [242].

#### FL118 exhibits high efficacy to eliminate CRC tumors

**Fig. 18:**
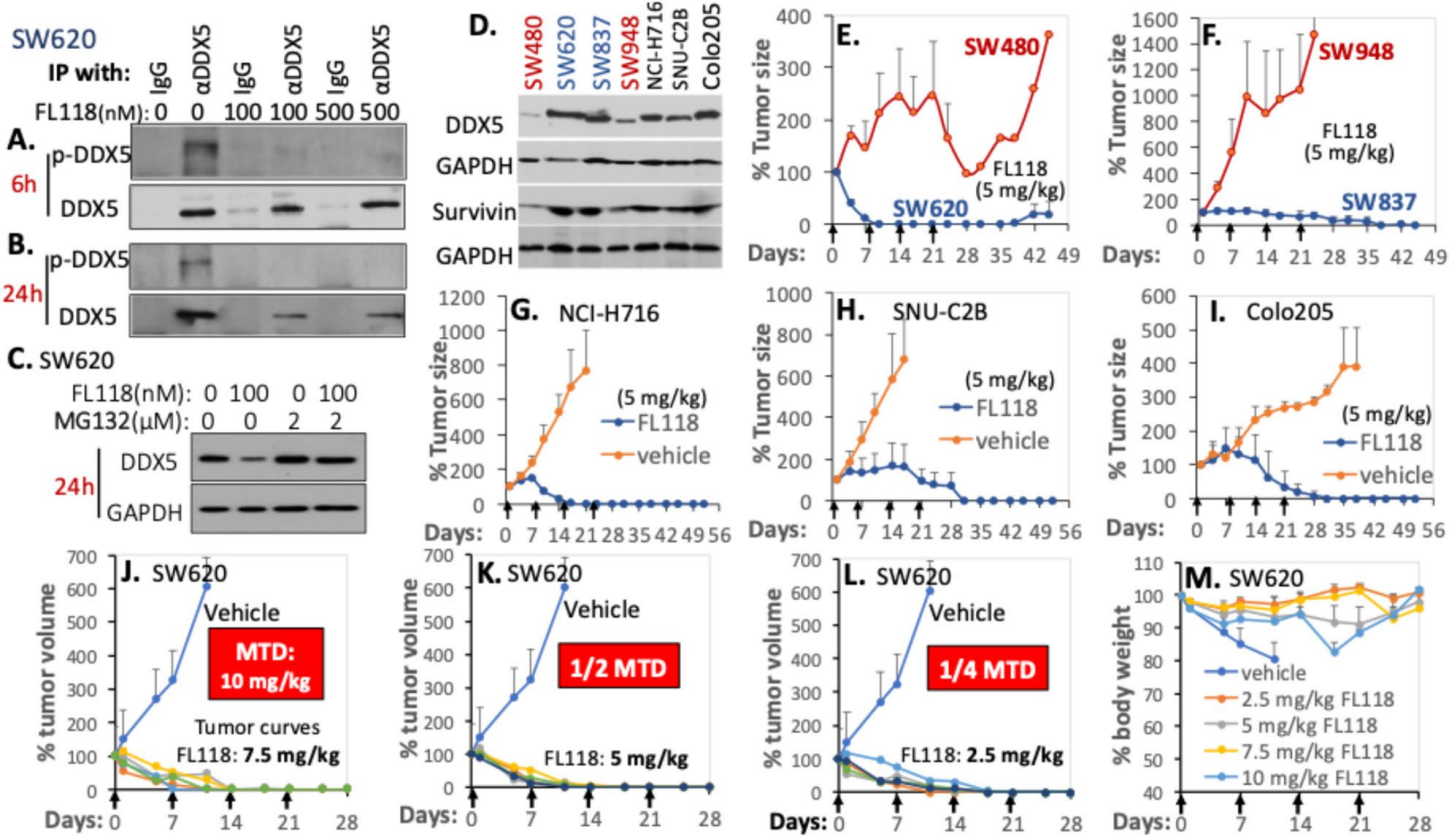
FL118 induces DDX5 dephosphorylation and degradation and links DDX5 levels to in vivo FL118 sensitivity in CRC models: (**A**, **B**) FL118 induces dephosphorylation and inhibition of DDX5. FL118 treatment for 6 h eliminates DDX5 phosphorylation in CRC SW620 cells (**A**); 24 h FL118 treatment maintains dephosphorylation and induces DDX5 degradation (**B**). SW620 cells treated with or without FL118 for 6 h (**A**) or 24 h (**B**) were analyzed by IP using anti-DDX5 antibody (αDDX5) or control IgG, followed by WB using anti–phosphotyrosine (αp-Y) and αDDX5. Input control (bottom panel in B) represents 10% of lysate prior to IP. **C** SW620 cells were treated with or without FL118 in the presence or absence of MG132 for 24 h, and DDX5 was analyzed by WB; GAPDH is a loading control. **D**-**I** High DDX5 in CRC tumors associates with high FL118 sensitivity in vivo. **D** DDX5 and survivin in 7 CRC cell lines by WB; GAPDH is a loading control. **E**, **F** Comparison of FL118 sensitivity in DDX5-low (SW480, SW948) versus DDX5-high (SW620, SW837) tumors. Vehicle tumor curves were removed for clarity (vehicle tumors reached maximum allowable size by ~ 3 weeks and were euthanized per IACUC). **G**-**I** FL118 sensitivity in three additional CRC xenografts (NCI-H716, SNU-C2B, Colo205) in SCID mice. Each line (2 × 10⁶ cells/site) was injected subcutaneously (2–3 mice/line) to establish tumors; experimental groups were treated with vehicle or FL118 (5 mg/kg; 1/2 MTD) orally weekly ×4 (arrows). Curves show mean tumor size + SD from 5 mice. **J**-**M** Antitumor activity of FL118 in a clinically and orally compatible formulation. SCID mice were implanted with 25–50 mg non-necrotic SW620 tumor tissue. At 100–200 mm³ (day 0), FL118 (10, 7.5, 5, or 2.5 mg/kg) was administered orally weekly ×4 (arrows) (3 females + 2 males/group/dose). **J**–**L** Vehicle curves show mean + SD from 5 mice; FL118-treated individual tumor curves from five mice/dose are shown. (**M**) Body weight changes (mean + SD; *n* = 5/group). This figure is adapted from our previous publications [41, 236] with new organization

We have demonstrated that: (i) FL118 selectively binds to DDX5 with high affinity (Fig. 13) [41]; (ii) in CRC SW620 cells, FL118 binding DDX5 induces rapid DDX5 dephosphorylation (≤ 6 h; Fig. 18A), followed by sustained dephosphorylation and DDX5 protein degradation (Fig. 18B), which can be rescued by MG132 (Fig. 18C) indicating via the proteasome degradation pathway; and (iii) FL118 sensitivity in human CRC tumors is strongly associated with DDX5 expression levels (Fig. 18D-I). Consistent with the high DDX5 expression observed in SW620 cells (Fig. 18D), a clinically and orally compatible formulation of FL118 drug products further enhance antitumor efficacy against CRC tumors (Fig. 18J-M) and can eliminate SW620 tumors even at one-quarter of its maximum tolerated dose (MTD) (Fig. 18L). Importantly, the highest tested dose (10 mg/kg, administered weekly for four weeks) caused only transient body-weight loss after the third dose, remaining within the acceptable ≤ 20% threshold (Fig. 18M).

#### UbE2T in CRC

Four publications between 2020 and 2025 demonstrated that: (i) UbE2T is highly expressed in CRC tumor samples compared with normal tissues and plays a key role in CRC progression [243]; (ii) knockdown (KD) of UbE2T inhibits CRC cell proliferation [244]; (iii) UbE2T facilitates ubiquitination and degradation of p53, thereby promoting CRC development [245]; and (iv) UbE2T contributes to chemoresistance in CRC [246]. Collectively, these findings highlight UbE2T as a potential diagnostic/prognostic biomarker and a therapeutic target in CRC.

### DDX5 and UbE2T in PDAC

#### DDX5 in PDAC

Currently, there is no effective treatment for pancreatic ductal adenocarcinoma (PDAC), which is characterized by poor drug delivery and resistance to chemotherapy, targeted therapy, and immunotherapy. PDAC is among the most lethal and difficult-to-treat malignancies, exhibiting high genomic instability, genetic heterogeneity, and metastatic potential [247, 248]. Genomic instability, combined with complex crosstalk among tumor cells, extracellular matrix (ECM), and stromal cells - as well as the desmoplastic barrier that restricts drug and cell-based therapy delivery within the tumor microenvironment (TME) - contributes to PDAC aggressiveness, treatment resistance, and high patient mortality [249]. Among these challenges, the lack of actionable molecular targets and effective strategies to overcome the immunosuppressive TME remain the two most urgent unmet needs. Current paradigms rely largely on modest single-agent activity combined with rationally designed, biomarker-directed drug combinations [250]. Nevertheless, transformative or breakthrough approaches are urgently needed for a possible cure; otherwise, aggressive PDAC may remain uncontrollable.

DDX5 is potentially involved in multiple DNA repair pathways and pathway crosstalk in cancer (Figs. 1 and 4). As such, DDX5 may play an important role in modulating genomic instability in PDAC cells and potentially reducing malignancy. Consistent with this notion, clinical studies using treatment-naïve PDAC specimens revealed that low DDX5 expression is associated with disease progression: median overall survival was 24 months in patients with low DDX5 expression versus 38 months in those with high DDX5 expression [251]. These findings suggest that high DDX5 expression in PDAC tumors may reduce genomic instability through enhanced DNA repair, thereby slowing tumor progression and contributing to improved prognosis. Nevertheless, DDX5 also appears to contribute to therapeutic resistance. Targeting DDX5 may therefore induce extensive DNA damage and apoptosis in cancer cells. For example, following treatment with the non-DDX5-targeting drug camptothecin (CPT), surviving cancer cells exhibit high DDX5 expression, whereas apoptotic cells display low DDX5 levels [252]. Moreover, high DDX5 expression is associated with increased expression of the immunosuppressive checkpoint factor CD24, and DDX5 participates in CD24-mediated therapy resistance in pancreatic cancer [32].

#### FL118 exhibits high efficacy in eliminating PDAC tumors alone or in combination with gemcitabine

**Fig. 19:**
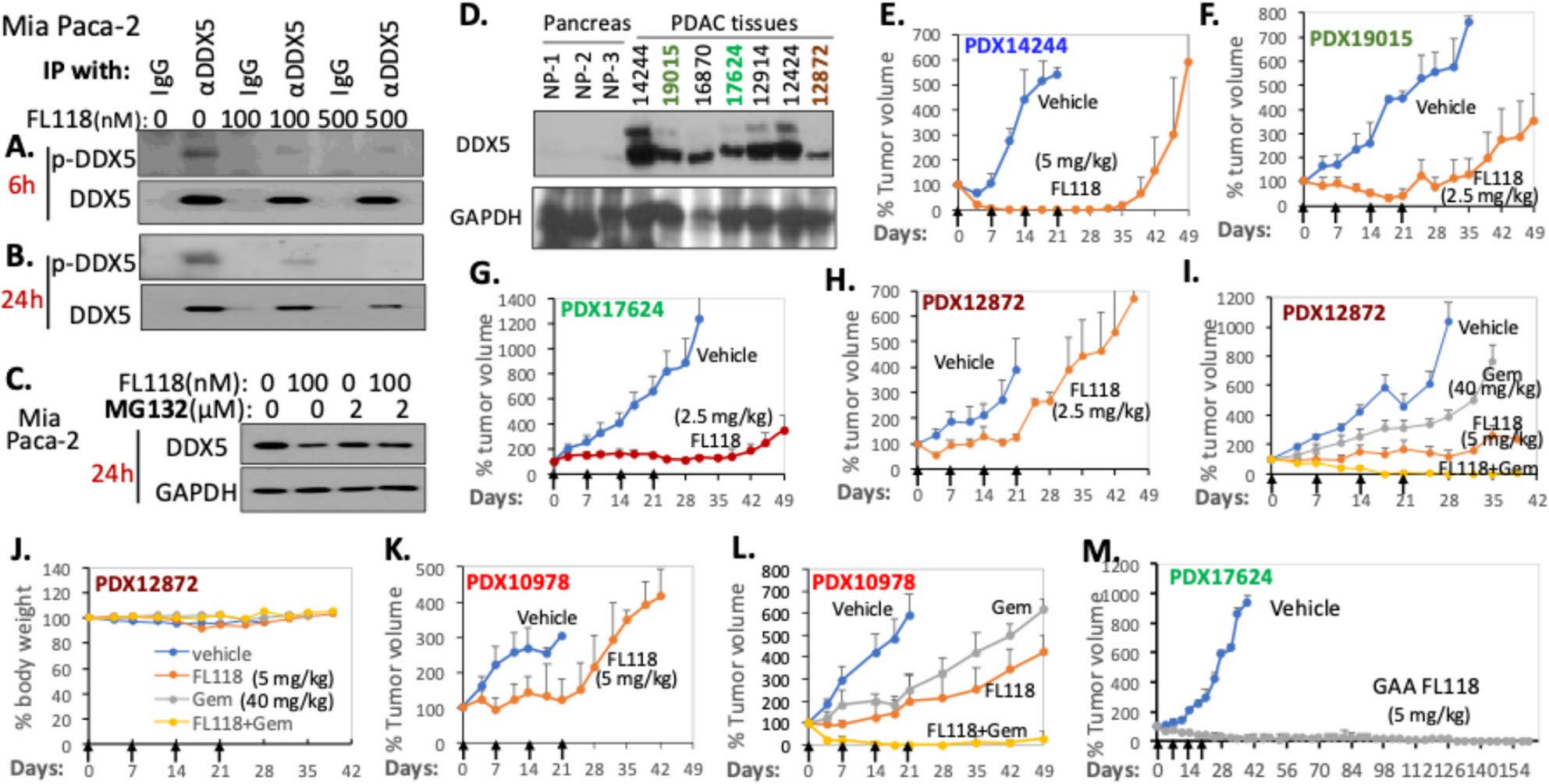
FL118 induces DDX5 dephosphorylation and degradation and links high DDX5 to FL118 sensitivity in PDAC PDX models: (**A**, **B**) FL118 induces DDX5 dephosphorylation and inhibition. Treatment of Mia PaCa-2 cells with FL118 for 6 h eliminates DDX5 phosphorylation (**A**); 24 h treatment maintains dephosphorylation and induces DDX5 degradation (**B**). Cells treated with or without FL118 for 6 h (**A**) or 24 h (**B**) were analyzed by IP with αDDX5 or control IgG, followed by WB with αp-Y and αDDX5. Input control (bottom panel in **B**) represents 10% of lysate prior to IP. **C** SW620 cells were treated with or without FL118 in the presence or absence of MG132 for 24 h, and DDX5 was analyzed by WB; GAPDH is a loading control. **D** DDX5 is elevated in PDAC PDX tumors. DDX5 was analyzed in three normal pancreas (NP) and seven PDAC tumor specimens by WB; GAPDH is a loading control. **E**-**M** PDAC PDX tumors with high DDX5 show high sensitivity to FL118. PDAC PDX tumors were maintained in SCID mice; experimental mice were implanted subcutaneously with individual PDX tumors. When tumors reached 100–300 mm³ (day 0), FL118 was administered intraperitoneally (**E**, **K**,** L**) or orally (**F**–**H**) at 2.5–5 mg/kg, with (**I**, **L**) or without (**E**, **F**, **G**, **H**, **K** gemcitabine as indicated. Curves represent mean + SD (*n* = 5 mice/group). **J** Body weight changes for experiment in (**I**). Notably, only (**M**) used clinically and orally compatible formulation-manufactured FL118 processed with glacial acetic acid (GAA); studies in (**E**-**L**) used preclinical formulation-manufactured FL118 suitable for intraperitoneal or oral administration. Data are adapted from our previous publications [41, 46, 236] with reorganized presentation

Consistent with prior studies, we demonstrated that FL118 strongly and selectively binds to DDX5 (Fig. 13) and subsequently induces DDX5 dephosphorylation (≤ 6 h) and degradation (≥ 24 h) in PDAC cells (Fig. 19A, B) [41], which can be rescued by MG132 (Fig. 19C), indicating via the proteasome degradation pathway. Using three normal human pancreas (NP) tissues and seven PDAC patient-derived xenograft (PDX) specimens, we found that NP tissues lack detectable DDX5 expression, whereas PDAC PDX tumors exhibit variable DDX5 expression levels (Fig. 19D) [41]. In line with DDX5 being a primary target of FL118, PDAC PDX tumor sensitivity to FL118 roughly correlates with DDX5 expression levels (compare Fig. 19E-H with Fig. 19D). Importantly, PDX tumors that were less sensitive to FL118 monotherapy (e.g., PDX12872 and PDX10978; Fig. 19H, K) could be effectively eliminated by combining FL118 with gemcitabine (Fig. 19I, L) [41, 46]. Finally, we demonstrated that a clinically and orally compatible formulation of FL118 significantly enhances antitumor activity [236]. For example, PDAC PDX17624 could not be eradicated by the preclinically compatible formulation of FL118 drug products (Fig. 19G) but was eliminated by the clinically and orally compatible formulation of FL118 drug products, with no tumor relapse observed for more than 154 days (Fig. 19M). Collectively, these results suggest that FL118 may achieve substantial antitumor efficacy in a significant subset of PDAC patients during clinical trials.

#### UbE2T in PDAC

Several studies have investigated the role of UbE2T in PDAC. Analyses of pancreatic cancer tissues and cell lines revealed that: (i) UbE2T expression is significantly elevated in pancreatic cancer cell lines compared with hTERT-HPNE cells and in tumor tissues compared with adjacent normal tissues [253]; (ii) UbE2T overexpression enhances pancreatic cancer cell proliferation, migration, and invasion; (iii) shRNA-mediated silencing of UbE2T inhibits these malignant phenotypes; and (iv) UbE2T overexpression downregulates α-catenin and E-cadherin while upregulating N-cadherin and vimentin, whereas UbE2T knockdown produces the opposite effects [253]. These findings indicate that UbE2T promotes epithelial–mesenchymal transition (EMT) and contributes to the aggressive behavior of pancreatic cancer cells.

**Fig. 20:**
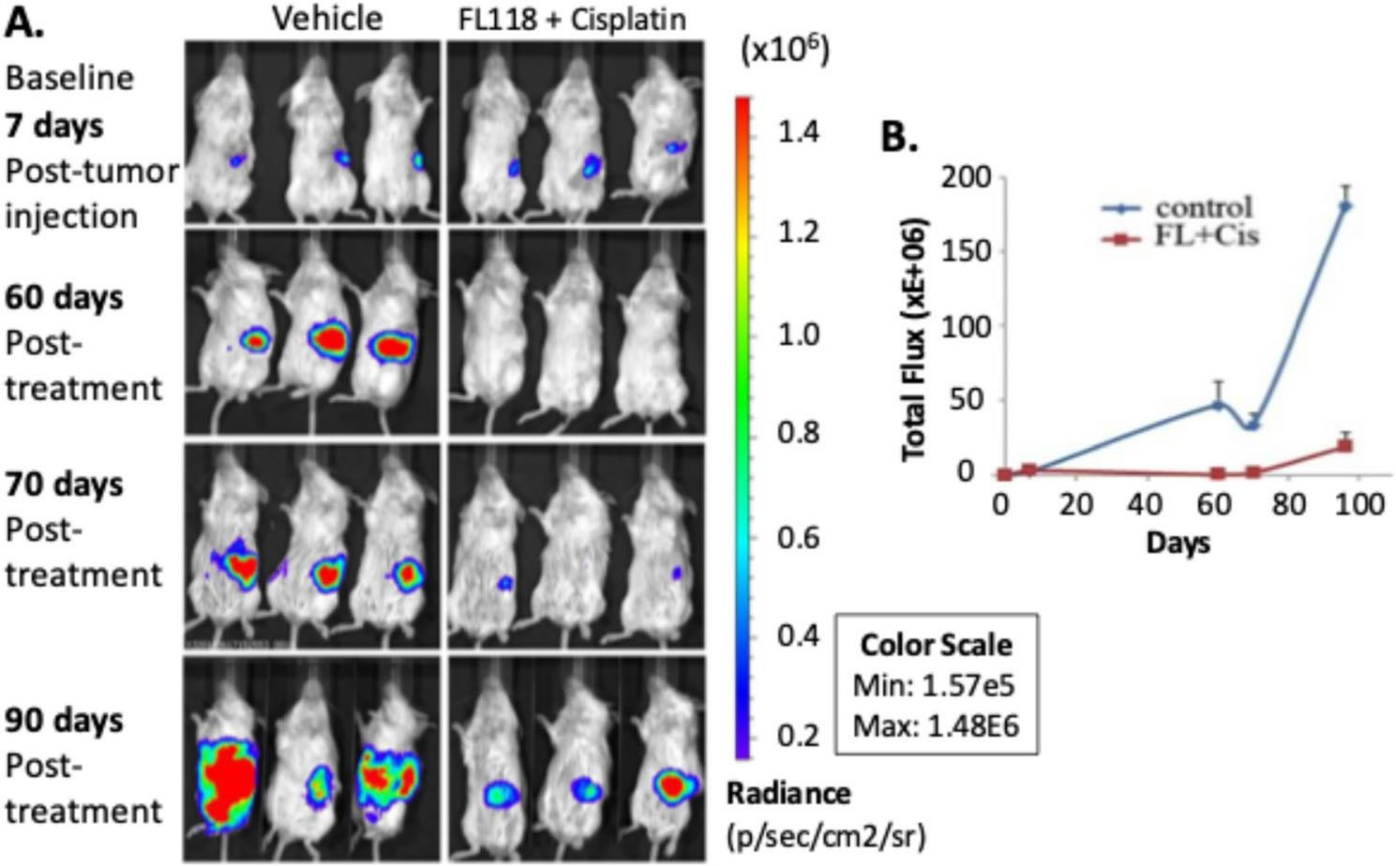
In vivo imaging of pancreatic tumor growth and metastasis using an orthotopic LucPAN-1 model: Seven days after orthotopic implantation of luciferase-labeled LucPAN-1 cells, mice were treated with or without FL118 (0.75 mg/kg) and cisplatin (5 mg/kg) on a weekly ×4 schedule. **A** Representative bioluminescent images captured on days 7, 60, 70, and 90 show tumor progression and response to treatment. **B** Tumor growth curve quantified from bioluminescence imaging (BLI) as total flux over time. This figure is adapted from our previous publication [46] with font adjustments

Jiang et al. further demonstrated that UbE2T represents a promising therapeutic target for overcoming gemcitabine resistance in pancreatic cancer [254], consistent with our FL118-gemcitabine combination studies showing curative potential (Fig. 19H, K). Using UbE2T-conditional knockout KPC mice, patient-derived organoids, PDX models, and clinical samples, they showed that: (i) KPC mice with UbE2T deletion exhibit significantly improved survival following gemcitabine treatment; (ii) UbE2T expression positively correlates with gemcitabine resistance in clinical patients; (iii) mechanistically, UbE2T facilitates RING1-mediated ubiquitination of p53, thereby relieving p53-dependent transcriptional repression of ribonucleotide reductase subunits RRM1 and RRM2 - key drivers of chemoresistance, metastasis, and metabolic adaptation - resulting in unrestrained pyrimidine biosynthesis and reduced replication stress; and (iv) combination therapy with the UbE2T inhibitor pentagalloylglucose (PGG) and gemcitabine suppresses PDX tumor growth and extends long-term survival in spontaneous pancreatic cancer mouse models [254]. These findings align well with our observation that FL118, when combined with cisplatin, effectively inhibits both tumor growth and metastasis in an orthotopic human Panc-1 pancreatic cancer model [46] (Fig. 20).

Collectively, these findings underscore the critical roles of both DDX5 and UbE2T in pancreatic cancer development, progression, treatment resistance, and metastasis. Accordingly, simultaneous targeting of DDX5 and UbE2T using a dual-target degrader such as FL118 represents an intriguing and potentially more effective therapeutic strategy for pancreatic cancer.

### DDX5 and UbE2T in osteosarcoma and ewing sarcoma

#### DDX5 in osteosarcoma and Ewing sarcoma

Osteosarcoma (OS) is characterized by complex disease development and metastasis originating from normal bone, with high genomic instability and rearrangements, extensive stroma–immune cell interactions, and poor drug delivery [255]. These features pose significant challenges for current treatment strategies and limit the likelihood of achieving durable cures.

**Fig. 21:**
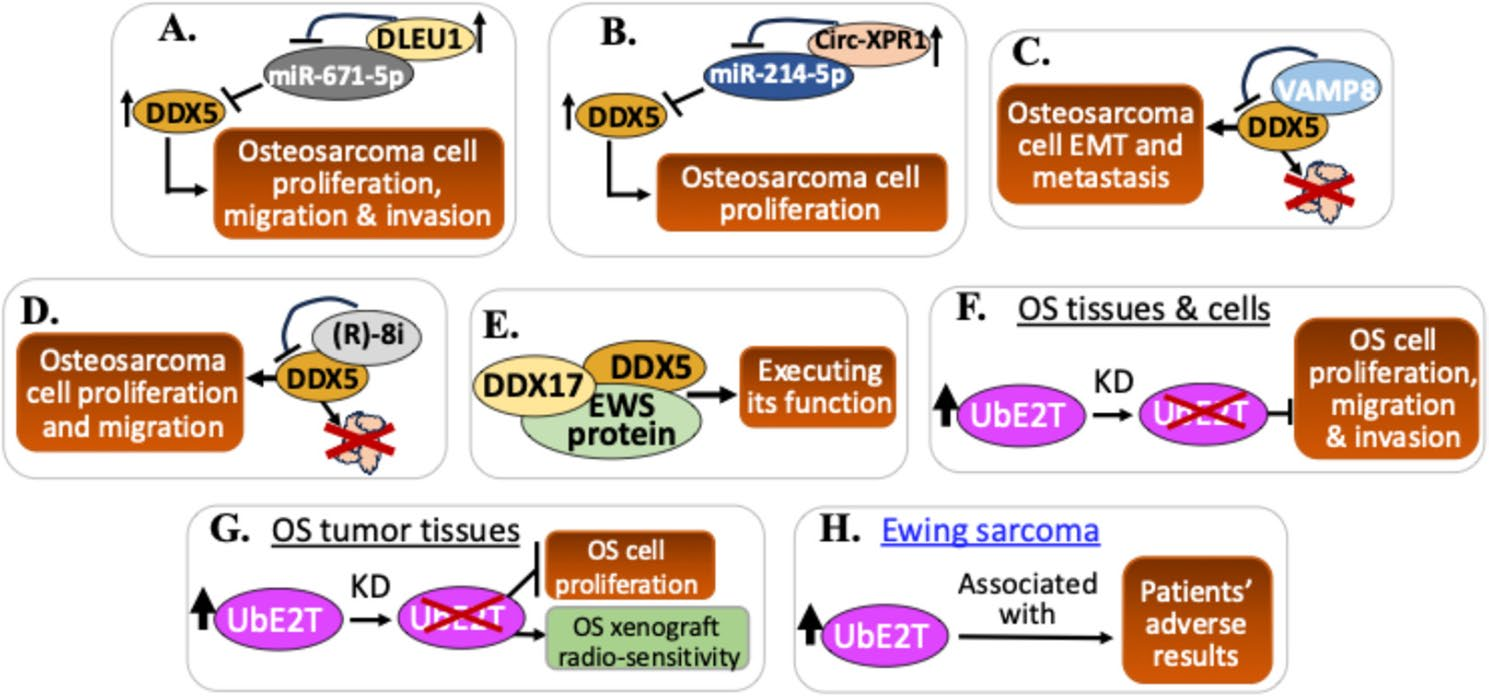
Functional roles of DDX5/p68 and UbE2T in osteosarcoma and Ewing sarcoma: (**A**) lncRNA DLEU1 uses DDX5 as an effector target. **B** Circular RNA circ-XPR1 uses DDX5 as an effector target. **C** Vesicle-associated membrane protein 8 (VAMP8) binds to, inhibits, and degrades DDX5 to inhibit osteosarcoma (OS) EMT and metastasis. **D** A thiazolone derivative, (R)-8i, binds to, inhibits, and degrades DDX5 to inhibit OS cell proliferation and migration. **E** Ewing sarcoma (EWS) protein interacts with DDX5 together with DDX17 to mediate its functions. **F** UbE2T is overexpressed in OS tissues and cell lines; UbE2T KD abrogates OS cell proliferation, migration, and invasion. **G** UbE2T is overexpressed in OS tissues; UbE2T KD blocks OS cell proliferation and enhances OS xenograft sensitivity to radiation. **H** High UbE2T expression is associated with adverse outcomes in Ewing sarcoma patients

Emerging evidence suggests that DDX5 is a critical therapeutic target in OS. Several studies have demonstrated its involvement in OS tumor progression: (i) the long noncoding RNA *DLEU1* exerts oncogenic effects by engaging DDX5 as a functional effector in osteosarcoma cells [256] (Fig. 21A); (ii) circular RNA circ-XPR1 promotes osteosarcoma cell proliferation by upregulating DDX5 expression through its signaling axis [257] (Fig. 21B); (iii) vesicle-associated membrane protein 8 (VAMP8), a tumor suppressor, binds to DDX5 and facilitates its proteasomal degradation, thereby inhibiting epithelial–mesenchymal transition (EMT) and metastasis [258] (Fig. 21C); and (iv) a thiazolone derivative, (R)-8i, identified through phenotypic screening, directly binds to DDX5 and induces its degradation, resulting in suppression of OS cell proliferation and migration [259] (Fig. 21D).

In addition, another study demonstrated that the Ewing sarcoma (EWS) protein interacts with p68/DDX5 and p72/DDX17 within the same protein complex to execute its oncogenic functions [260] (Fig. 21E), further implicating DDX5 in Ewing sarcoma biology.

### UbE2T in osteosarcoma and Ewing sarcoma

Recent studies have highlighted UbE2T as a promising therapeutic target in both osteosarcoma (OS) and Ewing sarcoma. Specifically: (i) UbE2T is overexpressed in OS tissues and cell lines, and its knockdown (KD) significantly inhibits cell proliferation, migration, and invasion (Fig. 21F), supporting its oncogenic role in OS [261]; and (ii) UbE2T overexpression in OS correlates with resistance to radiation therapy, whereas UbE2T KD not only suppresses OS tumor cell proliferation but also enhances radiation sensitivity in OS xenograft nude mouse models [262] (Fig. 21G).

Furthermore, bioinformatics analyses of publicly available datasets, together with immunohistochemistry (IHC) validation, revealed that UbE2T is highly expressed in Ewing sarcoma and possesses strong diagnostic potential [263]. Kaplan–Meier survival analyses and Cox regression models further demonstrated that elevated UbE2T expression is significantly associated with poor clinical outcomes in patients with Ewing sarcoma [263] (Fig. 21H).

### FL118 exhibits high efficacy in eliminating osteosarcoma (OS) tumors

**Fig. 22:**
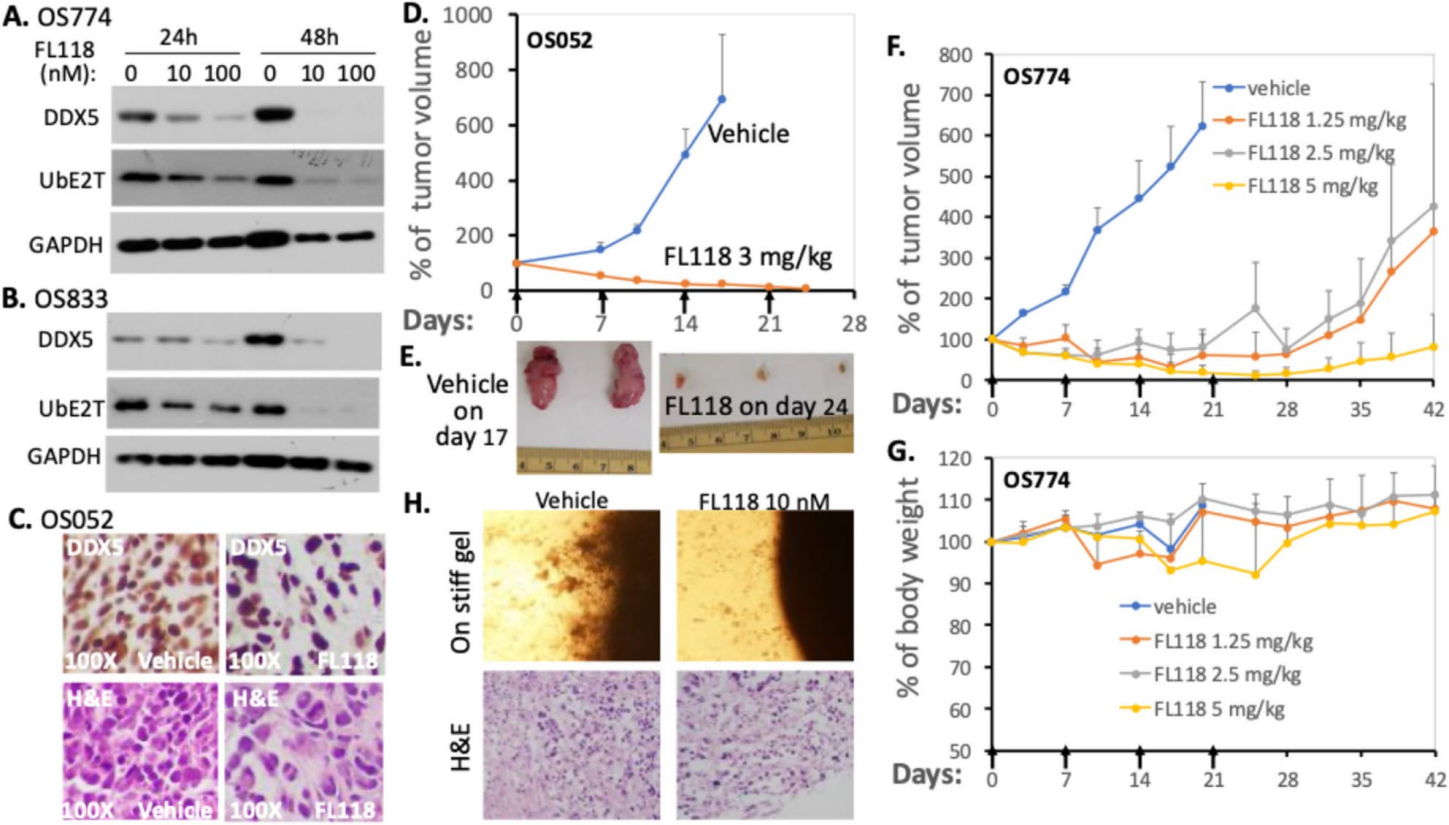
FL118 inhibits DDX5 and UbE2T in osteosarcoma models and modulates ECM/stroma features in cultured PDX tumors: (**A**,** B**) FL118 inhibits DDX5 and UbE2T in OS cells. OS PDX tumor cells were treated with or without FL118 for 24 h and 48 h as indicated, lysed, and analyzed for DDX5 and UbE2T by WB. **C** H&E staining and IHC analysis of DDX5 expression in vehicle- and FL118-treated OS052 tumors collected from SCID mice in (**D**). **D** FL118 exhibits high efficacy against OS052 tumors. OS052 PDX cell tumors were implanted into SCID mice; treatment with vehicle or FL118 (~ 1/3 MTD; 3 mg/kg) started when tumors reached 150–200 mm³. FL118 was administered orally weekly ×4 (arrows). Curves show mean tumor size + SD from 2 tumors (vehicle) and 3 tumors (FL118). **E** OS052 tumors collected at the end of (**D**). **F** FL118 regresses OS774 PDX tumors in SCID mice. OS774 tumors were implanted as in (**D**); treatment began at 150–200 mm³ with FL118 at 1/2 MTD (5 mg/kg), 1/4 MTD, or 1/8 MTD, orally weekly ×4. Curves show mean tumor size + SD from 5 mice. **G** Body weight changes during (**F**) (mean + SD; *n* = 5), within 10% loss (protocol allowed ≤ 20%). **H** Human PDAC PDX14244 tumors were isolated from SCID mice, cultured on a stiff gel, and treated with vehicle or FL118 for 7 days as shown. Upper panel: FL118 blocks ECM spreading. Lower panel: FL118 loosens ECM dense stromal stiffness. Tumors were fixed, paraffin-embedded, sectioned, and stained by H&E. Data shown are new and not previously published

Consistent with these literature findings, using OS PDX cell and tumor models established by Dr. Sweet-Cordero’s laboratory [264], our studies demonstrated that FL118 effectively suppresses OS tumor growth across multiple models. Specifically, FL118 reduced DDX5 and UbE2T expression in pediatric OS PDX tumor cells OS774 and OS833 (Fig. 22A, B), as well as in established OS052 xenograft tumors (Fig. 22C). Moreover, FL118 treatment significantly regressed OS052 tumors (Fig. 22D, E) and OS774 tumors (Fig. 22F), with minimal toxicity, as indicated by body-weight changes of less than 10% (Fig. 22G), well within the acceptable ≤ 20% limit defined in the animal protocol.

#### Dense stroma is not a barrier to FL118 delivery into the TME

Notably, the high efficacy of FL118 in eliminating PDAC tumors - either as a single agent or in combination with gemcitabine (Fig. 19) - indicates that FL118 can effectively penetrate the dense, stiff desmoplastic stroma characteristic of the PDAC tumor microenvironment (TME). Similarly, the strong antitumor activity of FL118 against OS tumors (Fig. 22D, F) suggests that FL118 delivery is not impeded, despite the well-documented extracellular matrix (ECM) deposition, fibroblastic stromal cell accumulation, and stromal activation in OS that can limit drug penetration.

We observed that both PDAC and OS tumors exhibit a loosening of stromal architecture following FL118 treatment. Specifically, immunohistochemical analysis of OS052 tumors isolated from the experiment shown in Fig. 22D revealed that FL118-treated tumors contained fewer cells and a looser stromal ECM (Fig. 22C, right panels) compared with vehicle-treated controls (Fig. 22C, left panels). Similarly, PDAC PDX14244 tumors isolated from SCID mice and subjected to ex vivo slice culture on stiff gel matrices showed that FL118 treatment for seven days blocked stromal outgrowth from the edges of tumor tissues (Fig. 22H, upper right compared with upper left images). Consistently, H&E-stained tissue sections demonstrated reduced ECM density following FL118 treatment (Fig. 22H, lower right compared with loq left images). These observations at least partially explain why FL118 does not encounter major delivery barriers in dense tumor stroma, although the underlying mechanisms remain to be elucidated.

**Fig. 23:**
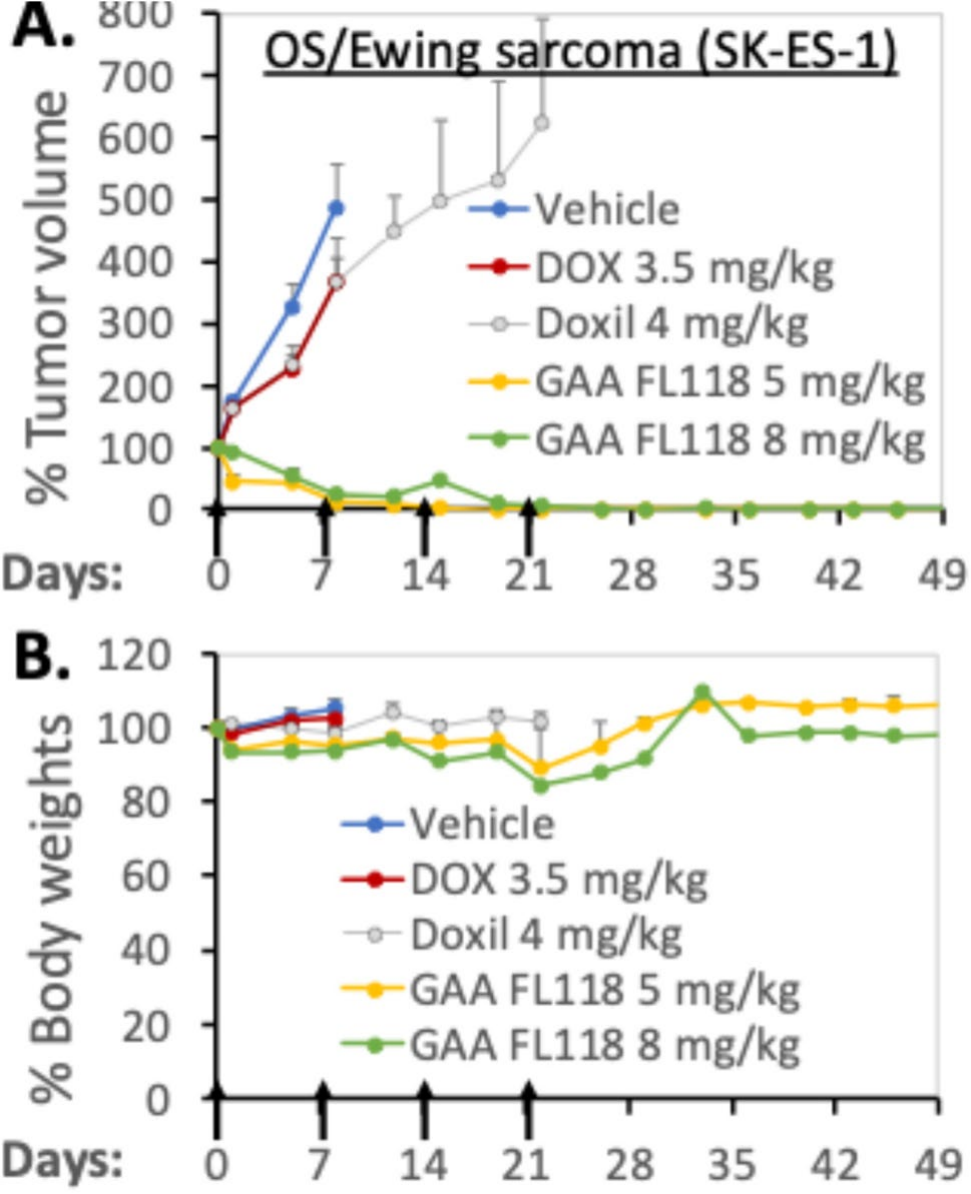
FL118 regresses human osteosarcoma/Ewing sarcoma tumors in SCID mice: (**A**) FL118, but not DOX or Doxil (liposome-formulated DOX), effectively eliminated OS/Ewing sarcoma tumors. SK-ES-1 cells (2 × 10⁶/site) mixed with 50% Matrigel were injected subcutaneously into flanks of 2–3 SCID mice to establish xenografts; tumors were then implanted into experimental SCID mice for efficacy studies. Treatment with vehicle, DOX, Doxil, or GAA-formulated clinically compatible FL118 at indicated doses was initiated at 150–200 mm³. FL118 and vehicle were given orally weekly ×4 (arrows), while DOX and Doxil were given intravenously once weekly for 3 weeks. Curves show mean tumor size + SD from 2–4 mice. **B** Body weight change curves (mean + SD; *n* = 2–4). Due to DOX toxicity, DOX-treated mice were euthanized on day 8 following the second IV dose (day 7), together with vehicle-treated mice. This figure is adapted from our recent publication [236]

#### FL118 exhibits high efficacy in eliminating Ewing sarcoma tumors

Finally, our previous studies using SK-ES-1 cell line-derived osteosarcoma/Ewing sarcoma xenograft tumors demonstrated that FL118 exhibits high efficacy in eliminating SK-ES-1 tumors (Fig. 23A) with acceptable toxicity (Fig. 23B). In contrast, doxorubicin (DOX) and its liposomal formulation (Doxil) showed only limited antitumor activity against SK-ES-1 tumors, even when administered at their MTD level (Fig. 23A).

### DDX5 and UbE2T in Ovarian Cancer (OC)

#### DDX5 in ovarian cancer

**Fig. 24:**
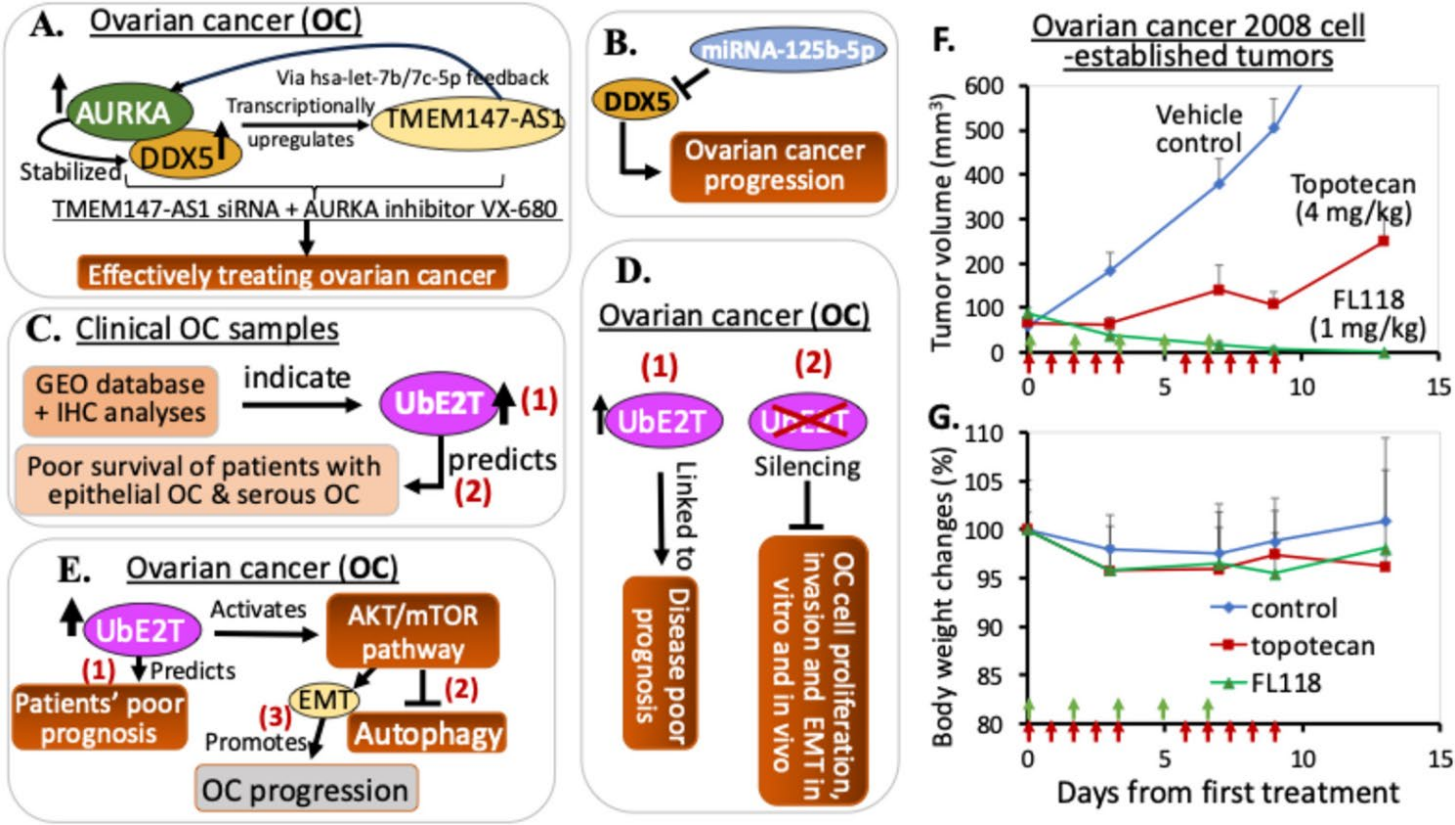
Functional roles of DDX5/p68 and UbE2T in ovarian cancer (OC): (**A**) AURKA binds DDX5 to form a transcriptional complex that upregulates TMEM147-AS1; AURKA expression is further increased via a hsa-let-7b/7c-5p–involved feedback loop. These findings support combining TMEM147-AS1 siRNA with the AURKA inhibitor VX-680 for enhanced OC therapy. **B** Bone mesenchymal stem cell–derived exosome-encapsulated miRNA-125b-5p downregulates DDX5, inhibiting OC progression. **C** Elevated UbE2T expression correlates with poor survival in epithelial and serous OC. **D** UbE2T is highly expressed in OC and associates with poor prognosis (1); UbE2T silencing inhibits OC cell proliferation, invasion, and EMT in vitro and in vivo (2). **E** Upregulated UbE2T predicts poor prognosis (1), activates AKT/mTOR signaling to suppress autophagy (2), and promotes EMT to drive OC progression (3). **F **FL118 eliminates human 2008 °C xenografts, whereas topotecan delays tumor growth. A suspension of 1 × 10⁷ viable 2008 °C cells/mL in 50% Matrigel was injected subcutaneously (0.1 mL/site) into female SCID mice to establish tumors (10–14 days). Mice were treated with FL118 (1 mg/kg, qd2 × 5; green arrows), topotecan (4 mg/kg, daily ×5 for 2 cycles; red arrows), or vehicle (daily ×5). Curves show mean tumor size + SD (*n* = 5). **G** Body weight changes (mean + SD; *n* = 5). Panels (**F**, **G**) present new, unpublished data

The role of DDX5/p68 in ovarian cancer is only beginning to emerge. Two recent publications revealed that: (i) Aurora kinase A (AURKA) directly binds to DDX5 to form a transcriptional coactivator complex that induces transcriptional upregulation of the oncogenic long noncoding RNA TMEM147-AS1 [265]. This occurs through a hsa-let-7b/7c-5p–mediated feedback loop that further increases AURKA expression, providing a rationale for combining TMEM147-AS1 siRNA with the AURKA inhibitor VX-680 for more effective ovarian cancer treatment (Fig. 24A) [265]; and (ii) bone mesenchymal stem cell–derived exosome-encapsulated miRNA-125b-5p inhibits ovarian cancer progression by downregulating DDX5 (Fig. 24B) [266].

#### UbE2T in ovarian cancer

Multiple studies have established a critical role for UbE2T in ovarian cancer progression and prognosis. Specifically: (i) IHC and Gene Expression Omnibus (GEO) database analyses revealed that UbE2T is significantly upregulated in epithelial ovarian cancer (EOC) compared with borderline tumors, benign tumors, and normal ovarian tissues (Fig. 24C1) [267]. High UbE2T expression is associated with poor prognosis and serves as an independent risk factor for both EOC and serous ovarian cancer (SOC), supporting its potential as a prognostic marker and therapeutic target (Fig. 24C2) [267]; (ii) UbE2T is highly expressed in ovarian cancer and closely associated with poor clinical outcomes (Fig. 24D1), and silencing UbE2T significantly inhibits ovarian cancer cell proliferation, invasion, and epithelial–mesenchymal transition (EMT) both in vitro and in vivo (Fig. 24D2) [268]; and (iii) UbE2T upregulation predicts poor prognosis in ovarian cancer (Fig. 24E1) [269]. Mechanistically, high UbE2T expression activates the AKT/mTOR signaling axis to promote malignant progression by suppressing autophagy (Fig. 24E2), thereby enhancing EMT (Fig. 24E3) [269].

#### FL118 exhibits high efficacy in eliminating ovarian cancer xenograft tumors

Consistent with the important roles of DDX5 and UbE2T in ovarian cancer, we evaluated the antitumor efficacy of FL118 using xenograft tumors established from the p53-mutant human ovarian cancer cell line 2008. Our results demonstrated that FL118, administered at 1 mg/kg - a dose below its maximum tolerated dose (MTD) - effectively eliminated 2008-derived xenograft tumors in SCID mice (Fig. 24F). In contrast, topotecan, administered at its MTD (~ 4 mg/kg), only delayed tumor growth (Fig. 24F). These findings highlight the strong therapeutic potential of FL118 for the treatment of human ovarian cancer.

### DDX5 and UbE2T in Glioma and Glioblastoma Multiforme (GBM)

#### DDX5 in glioma and glioblastoma

**Fig. 25:**
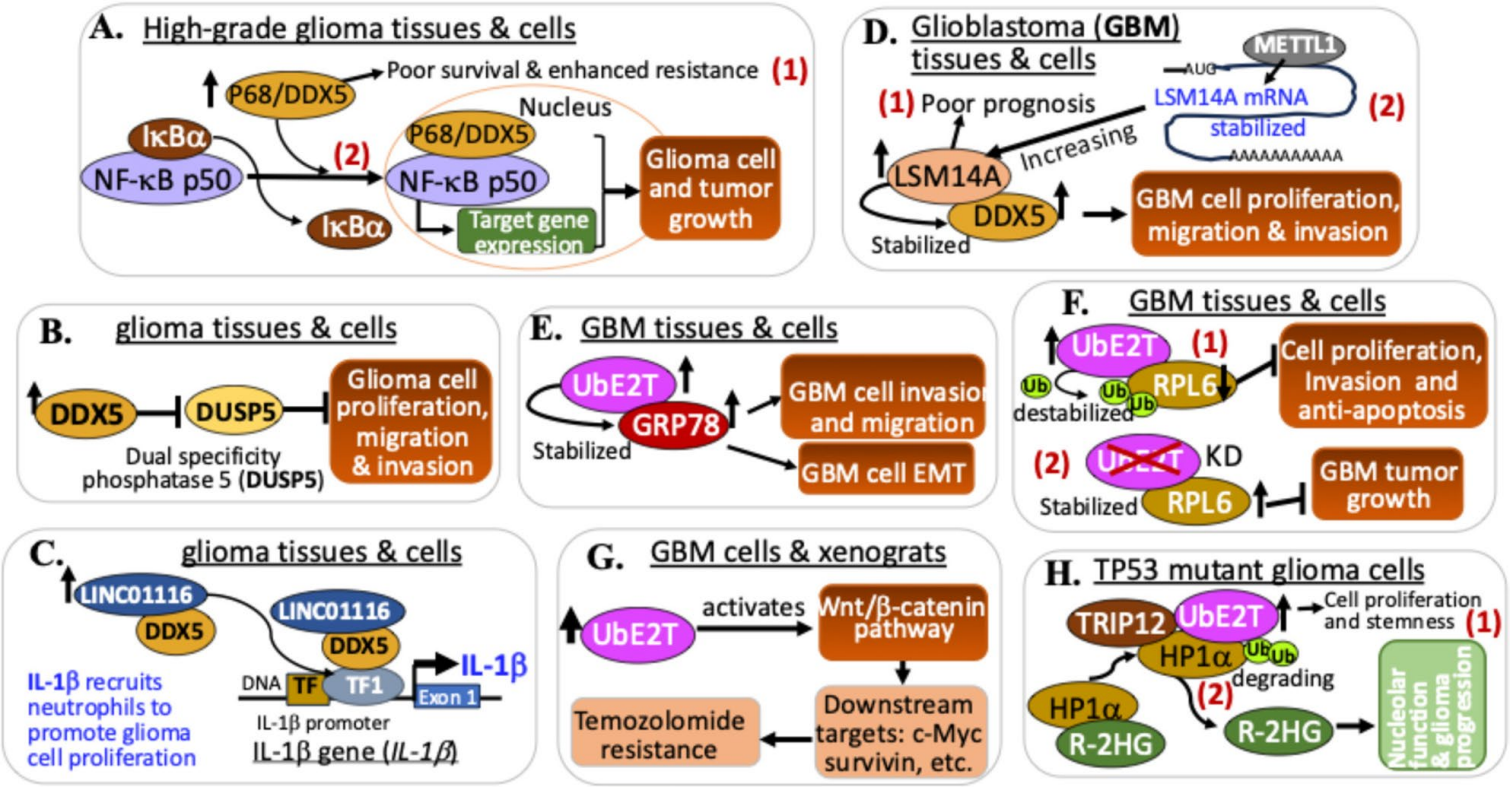
Functional roles of DDX5/p68 and UbE2T in glioma and glioblastoma (GBM): (**A**) Elevated p68/DDX5 associates with high-grade gliomas, poorer overall survival, and resistance to radiotherapy plus temozolomide (see text and panel A image). **B** DDX5/p68 downregulates DUSP5, relieving inhibitory effects on glioma proliferation, migration, and invasion. **C** lncRNA LINC01116 recruits DDX5 to the IL-1 promoter, activating IL-1β expression and promoting glioma proliferation and neutrophil recruitment. **D** In GBM, RNA-binding protein LSM14A stabilizes DDX5 via the METTL1–LSM14A–DDX5 pathway to promote oncogenic functions. **E** UbE2T promotes GBM invasion and migration by stabilizing GRP78 and promoting EMT. **F** UbE2T binds, ubiquitinates, and reduces ribosomal protein L6 (RPL6) (1); UbE2T KD suppresses GBM tumor growth (2). **G** UbE2T promotes GBM resistance to temozolomide by activating Wnt/β-catenin signaling, upregulating downstream targets including survivin and c-Myc. **H** UbE2T degrades HP1α, releasing suppressive effects of R-2HG and promoting glioma proliferation and stemness

Previous studies have identified multiple mechanisms by which DDX5/p68 contributes to glioma and glioblastoma progression. Specifically: (i) p68/DDX5 expression is significantly elevated in high-grade gliomas compared with low-grade gliomas and adjacent normal brain tissues [270]. High DDX5 expression correlates with poorer overall survival and increased resistance to radiotherapy plus temozolomide (Fig. 25A1) [270]. Mechanistically, DDX5 promotes glioma cell proliferation in vitro and tumor growth in vivo by binding to NF-κB p50 and regulating downstream target genes (Fig. 25A2) [270]; (ii) DDX5/p68 enhances glioma cell proliferation, migration, and invasion by downregulating dual-specificity phosphatase 5 (DUSP5) (Fig. 25B) [271]; (iii) the long noncoding RNA LINC01116 recruits DDX5 to the *IL1* promoter, activating IL-1β expression in glioma cells and thereby promoting tumor proliferation and neutrophil recruitment (Fig. 25C) [212]; and (iv) in glioblastoma, overexpression of the RNA-binding protein LSM14A is associated with poor prognosis (Fig. 25D1) [272]. LSM14A promotes GBM cell proliferation, migration, and invasion in vitro and in xenograft models by binding to and stabilizing DDX5. This effect is mediated by METTL1-dependent stabilization of LSM14A mRNA, which enhances LSM14A expression and reinforces DDX5 activity (Fig. 29D2) [272].

#### UbE2T in glioma and glioblastoma

Multiple reports have defined critical roles for UbE2T in glioma and glioblastoma (GBM) biology. Specifically: (i) UbE2T promotes GBM cell invasion and migration by stabilizing GRP78, a chaperone protein associated with high-grade GBM and resistance to chemotherapy and radiotherapy, and by facilitating EMT (Fig. 25E) [273]; (ii) UbE2T is overexpressed in GBM and contributes to malignancy by binding to, ubiquitinating, and degrading ribosomal protein L6 (RPL6) (Fig. 25F1). Knockdown (KD) of UbE2T markedly suppresses GBM tumor growth (Fig. 25F2) [274]; (iii) UbE2T enhances GBM resistance to temozolomide by activating the Wnt/β-catenin signaling pathway and upregulating downstream effectors such as Survivin and c-Myc (Fig. 25G) [275]; and (iv) UbE2T is overexpressed in gliomas harboring *TP53* mutations and is associated with increased glioma cell proliferation and stemness (Fig. 25H1) [276]. Mechanistically, UbE2T, in coordination with the E3 ligase TRIP12, promotes ubiquitin-proteasome-mediated degradation of heterochromatin protein 1α (HP1α), thereby relieving the suppressive effects of R-2-hydroxyglutarate (R-2HG) on nucleolar function and glioma proliferation (Fig. 25H2) [276].

### ABCG2-expressing cancer cells show resistance to common anticancer agents but not to FL118

ABCG2 (ATP-binding cassette sub-family G member 2), also known as BCRP (breast cancer resistance protein), plays a critical role in cancer cell resistance to therapy [277]. ABCG2 is localized on the plasma membrane of cancer cells and is widely implicated in cancer stem cell (CSC) biology. This intrinsic ABCG2-mediated drug resistance in CSCs is a fundamental driver of cancer relapse and recurrence following treatment. Together with ABCB1 (also known as MDR1 or Pgp), ABCG2 induces broad chemotherapy resistance in many advanced, difficult-to-treat cancers, including osteosarcoma and Ewing sarcoma [278]. Recent studies have further linked ABCG2 expression to resistance to chemoradiotherapy, disease progression, higher tumor grade, and reduced patient survival in human glioma [279]. Functionally, ABCG2 limits the oral bioavailability and brain penetration of many anticancer agents and mediates treatment resistance by actively pumping drugs out of cancer cells, thereby reducing their intracellular concentrations and therapeutic efficacy (Table 1).

**Table 1:** ABCG2/BCRP-mediated resistance to various classes of anticancer agents (compiled from multiple online websites)

Topoisomerase Inhibitors			
Drug Class	Examples	ABCG2 Effect	Key Notes
Topoisomerase I inhibitors	Topotecan, Irinotecan (SN-38), Camptothecin (CPT) derivatives	Strong efflux	ABCG2 efficiently transports topotecan and SN-38; overexpression confers strong resistance.
Topoisomerase II inhibitors	Mitoxantrone, Doxorubicin, Epirubicin	Strong efflux	Mitoxantrone is a prototypical ABCG2 substrate; resistance often used as assay marker.
Antimetabolites			
Purine analogs	6-Mercaptopurine, Cladribine	Moderate	ABCG2 exports some purine analogs and their nucleoside metabolites.
Methotrexate and antifolates	Methotrexate, Pemetrexed	Moderate to strong	ABCG2 can efflux methotrexate and certain antifolate conjugates; resistance varies by cell type.
Tyrosine Kinase Inhibitors (TKIs)			
Imatinib, Dasatinib		Substrate and inhibitor	Dual behavior—can both be transported and inhibit ABCG2.
Gefitinib, Erlotinib		Substrate/inhibitor	At high concentrations, can inhibit ABCG2; at low concentrations, are transported.
Sorafenib, Sunitinib, Lapatinib, Crizotinib		Variable	Resistance linked to ABCG2 overexpression; some TKIs downregulate ABCG2 expression over time.
Antitumor Antibiotics and Natural Products			
Anthracyclines	Doxorubicin, Epirubicin	Moderate	ABCG2 confers partial resistance; ABCB1 (Pgp) usually stronger contributor.
Flavopiridol and other flavonoids	Flavopiridol	Strong	ABCG2 exports flavonoids and related compounds.
Porphyrins and photosensitizers	Photofrin, Verteporfin	Strong	ABCG2 limits photodynamic therapy efficiency.
Microtubule-Targeting Agents			
Taxanes	Paclitaxel, Docetaxel	Weak to moderate	Pgp is the dominant efflux transporter; ABCG2 plays a minor role.
Epothilones	Ixabepilone	Substrate	ABCG2-mediated resistance documented in some lines.
Others *			
Photosensitizers / Porphyrins	Talaporfin, Verteporfin	Strong	Affects photodynamic therapy outcomes.
PARP inhibitors	Olaparib, Rucaparib	Moderate	ABCG2 reduces intracellular accumulation and limits BBB penetration.
mTOR inhibitors	Temsirolimus, Everolimus	Moderate	ABCG2 decreases efficacy and brain availability.

* For platinum agents (e.g., cisplatin, carboplatin, oxaliplatin), ABCG2 exhibits minimal to no resistance. Resistance to these agents typically occurs through alternative mechanisms (e.g., DNA repair)

### FL118 is not affected by ABCG2-, ABCB1-, or ABCC1-mediated drug resistance

**Fig. 26:**
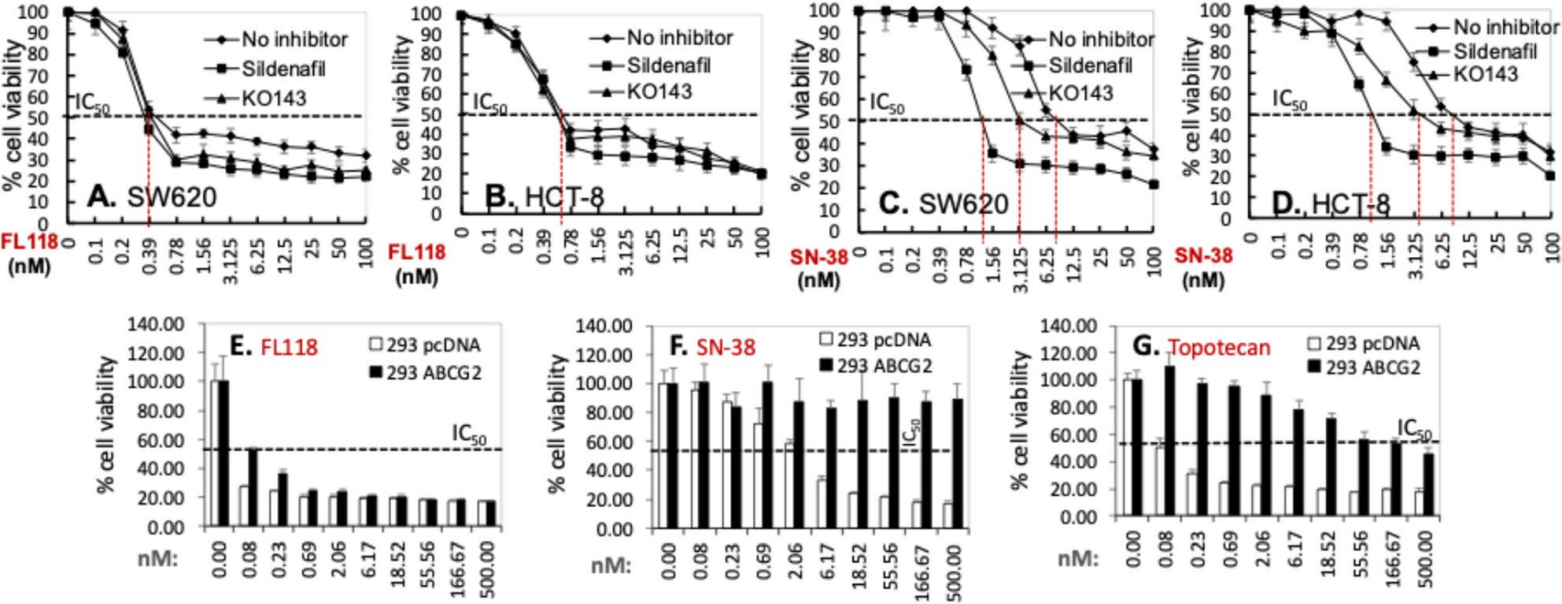
FL118 appears to bypass resistance mediated not only by ABCG2 (BCRP) but also by other ABC transporters: Sub-confluent SW620 and HCT-8 CRC cells were treated with FL118 (**A**,** B**) or SN-38 (**C**,** D**) at indicated concentrations in the presence or absence of sildenafil (50 µM; ABCG2/Pgp/MRP1 triple inhibitor) or KO143 (2 µM; ABCG2 inhibitor) for 3 days. Viability was assessed by MTT. Each time point represents mean ± SD from 3–5 independent experiments. Notably, pre-testing confirmed that sildenafil (50 µM) and KO143 (2 µM) were non-toxic. **E** ABCG2 overexpression (OE) in HEK293 cells did not affect FL118 activity. In contrast, ABCG2 OE blocked SN-38 (**F**) and topotecan (**G**) activity. HEK293 cells stably transfected with pcDNA3 (control) or pcDNA3-ABCG2 were treated with FL118 (**E**), SN-38 (**F**), or topotecan (**G**) (0-500 nM) for 72 h, and viability was measured by MTT. Bars represent mean + SD from 5 independent experiments. This figure is adapted from our previous publications [281, 282]

In contrast to many anticancer agents listed in Table 1 and consistent with our previous finding that [1] pharmacological inhibition of ABCG2 modulates the potency of SN-38, but not FL118; and [2] genetic silencing or overexpression of ABCG2 showing the potency of FL118 unaffected by ABCG2 [280], FL118 is not affected by resistance mechanisms mediated by ABCG2 (BCRP), ABCB1 (Pgp/MDR1), or ABCC1 (MRP1). Specifically, our studies revealed that FL118 maintains consistent IC₅₀ values in both SW620 (Fig. 26A) and HCT-8 (Fig. 26B) cells, regardless of the presence or absence of the ABCG2-specific inhibitor Ko143 or the broad-spectrum inhibitor sildenafil, which inhibits ABCG2, Pgp, and MRP1.

In contrast, SN-38–treated SW620 (Fig. 26C) and HCT-8 (Fig. 26D) cells exhibited significantly reduced IC₅₀ values when Ko143 was used to inhibit ABCG2. Moreover, the addition of sildenafil led to an even greater reduction in IC₅₀ compared with Ko143 alone, indicating that SN-38 is a substrate not only of ABCG2 but also of Pgp and MRP1.

To further validate these findings, we overexpressed ABCG2 in HEK293 cells and found that ABCG2 overexpression had no negative impact on the inhibitory activity of FL118 (Fig. 26E). In contrast, ABCG2 overexpression significantly reduced the efficacy of SN-38 (Fig. 26F) and topotecan (Fig. 26G), demonstrating strong resistance to both agents.

Collectively, these findings highlight a key additional advantage of FL118 and clearly distinguish it from other anticancer therapeutics, including camptothecin (CPT) analogs such as irinotecan/SN-38 and topotecan (Fig. 26).

## FL118 has favorable pk and toxicology profiles

### FL118 showed favorable Pharmacokinetics (PK) Profiles

**Fig. 27:**
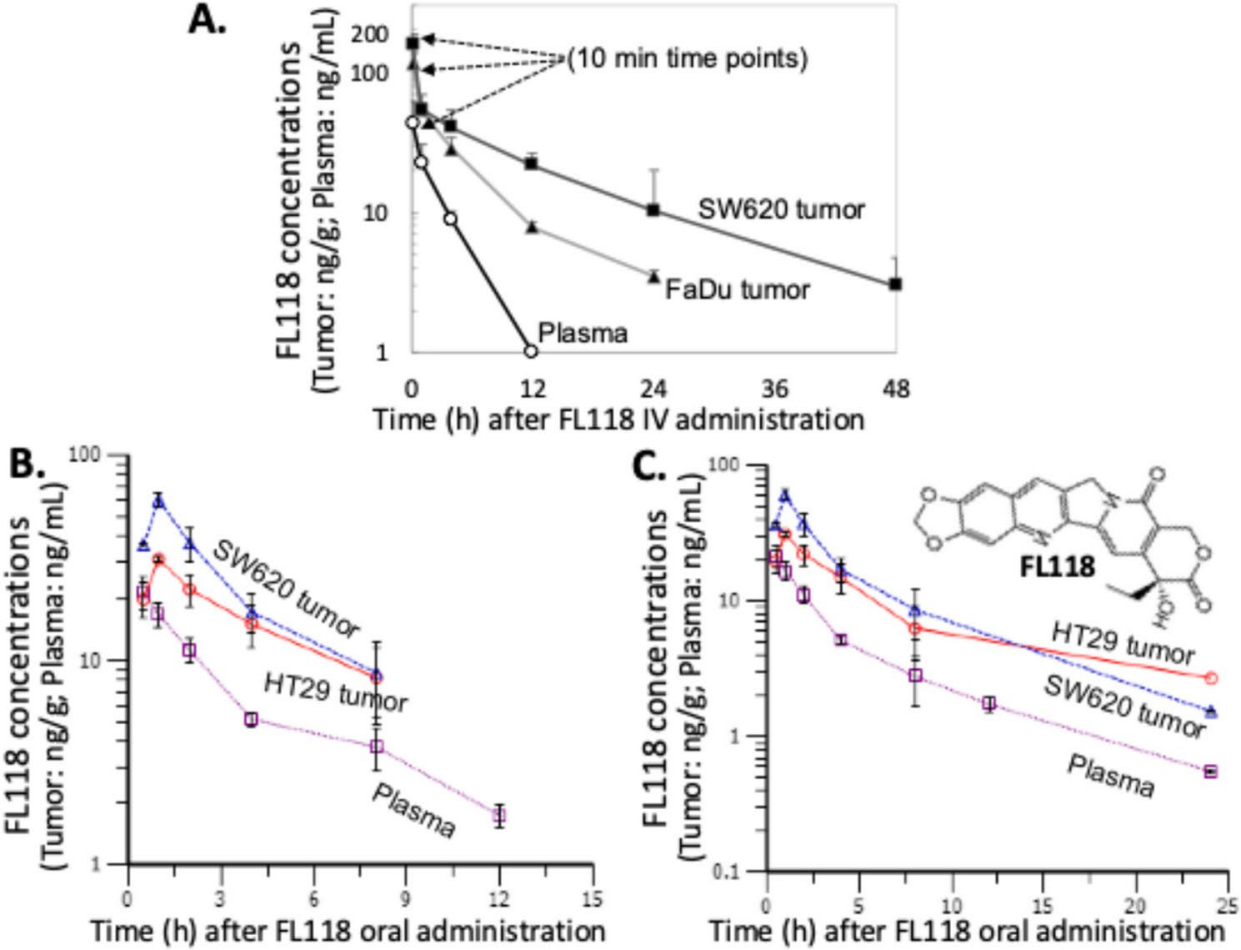
FL118 exhibits favorable pharmacokinetic (PK) profiles after intravenous (IV) or oral (po) administration: (**A**) IV PK: SCID mice were subcutaneously implanted with human FaDu and SW620 tumors. When tumors reached 800–1000 mm³, FL118 was IV-injected once at 1.5 mg/kg. Blood and tumor tissues were collected at 10 min, 1 h, 4 h, 12 h, 24 h, and 48 h. **B**,** C** Oral PK: three SCID mice per time point were subcutaneously implanted with human SW620 and HT29 tumors. When tumors reached 800–1000 mm³, FL118 was orally administered once at 1.5 mg/kg. Blood and tumor tissues were collected at 30 min, 1 h, 2 h, 4 h, 12 h, and 24 h. Each curve in (**A**-**C**) represents mean ± SD from 3 tumors or plasma samples. Data in (**A**) are adapted from our previous publication [281], whereas (**B**, **C**) present newly generated, unpublished data

We found that FL118 accumulates in human tumors following either intravenous (IV) (Fig. 27A) [283] or oral (PO) (Fig. 27B, C) administration, while being rapidly cleared from the bloodstream (plasma) (Fig. 27). This tumor-selective accumulation may, at least in part, underlie the low toxicity observed in animals.

Consistent with these findings, FL118 administered via either IV (Table 2) [283] or PO (Table 3) routes exhibited favorable PK profiles. For example, the area under the curve (AUC) was higher in human tumors than in mouse plasma (Tables 2 and 3). As shown in Table 2 (IV, single dose at 1.5 mg/kg), the ratios of AUC ∞ for FaDu tumor versus AUC ∞ for plasma and AUC ∞ for AW620 tumor versus AUC ∞ for plasma were 4.31 and 8.63, respectively, indicating a multifold increase in FL118 accumulation in tumors compared with plasma. A similar trend was observed for the PO route, as shown in Table 3. Furthermore, oral bioavailability of FL118 was calculated to exceed 84% (Table 3), which is notable. Given this excellent oral bioavailability, the PO route has been selected for future clinical trials.

**Table 2:** Pharmacokinetics parameters of FL118 in human tumor and mouse plasma following single-dose intravenous (**i.v.**/**iv**/**IV**) administration of 1.5 mg/kg FL118 *

Matrix	T1/2 (hr)	Tmax (hr)	Cmax (ng/g, ml)	AUC (hr*ng/g)	AUC∞ (hr*ng/g)	AUC% Extrap (%)	Vz (g/kg) (ml/kg)	Cl (g/hr/kg) (ml/hr/kg)
FaDu	6.852	0.167	115	413	448	7.74	33,052	3343
SW620	12.75	0.167	158	842	897	6.17	30,742	1671
Plasma	1.788	0.167	43	82	104	21.7	36,849	14,287
Ratio of AUC_∞_for FaDu/ AUC_∞_for Plasma					448/104 = 4.31			
Ratio of AUC _∞_ for SW620/ AUC_∞_for Plasma					897/104 = 8.63			

* adapted from our previous publication [281] with updated calculations

**Table 3:** Pharmacokinetics parameters of FL118 in human tumor and mouse plasma following single-dose oral (**p.o.**/**po**/**PO**) administration of 1.5 mg/kg FL118 ^#^

Matrix	T_max_	t_1/2_	C_max_	SE C_max_	AUC_all_	AUC_∞_	AUC 0–8	V_D_	Cl
	(hr)	(hr)	(ng/ml)	(ng/ml)	(hr*ng/ml)	(hr*ng/ml)	(hr*ng/ml)	(ml/kg)	(ml/hr/kg)
Plasma	0.50	5.12	21.4	3.88	74.1	88.0	63.0	126,000	17,000
Matrix	T_max_	t_1/2_	C_max_	SE C_max_	AUC_all_	AUC_∞_	AUC 0–8	V_D_	Cl
	(hr)	(hr)	(ng/g)	(ng/g)	(hr*ng/g)	(hr*ng/g)	(hr*ng/g)	(g/kg)	(g/hr/kg)
HT29	1.00	4.24	31.2	0.822	128	178	128	51,700	8,440
SW620	1.00	2.99	60.5	4.74	188	224	188	28,900	6,710
Ratio of AUC_∞_ for HT29 / AUC_∞_for Plasma						178/88 = 2.02			
Ratio of AUC_∞_ for SW620 / AUC_∞_for Plasma						224/88 = 2.55			
Oral Bioavailability for FL118 (PO/IV)						*88/104 = 84.6%*			

^#^ Table 3 presents newly generated data associated with the data set presented in Fig. 18BC

### FL118 toxicology profile in rats and dogs

Consistent with the favorable PK profile of FL118 observed in human tumor mouse models, FL118 products formulated in a preclinically compatible oral formulation exhibited a favorable toxicology profile in beagle dogs, with a maximum tolerated dose (MTD) of 2.2 mg/kg [46]. Furthermore, FL118 products manufactured using a clinically and orally compatible formulation demonstrated even more favorable toxicology profiles in Sprague Dawley (SD) rats and beagle dogs.

Specifically, (i) the rat MTD derived from preclinically and orally compatible FL118 products at ≤ 1.65 mg/kg shifted to ≥ 2.44 mg/kg in male rats and ≤ 4.77 mg/kg in female rats when clinically and orally compatible FL118 products were used [236]; and (ii) the beagle dog MTD derived from preclinically orally compatible FL118 products at 2.2 mg/kg shifted to 5 mg/kg when clinically and orally compatible FL118 products were used [236].

However, we should point out that, although FL118 has shown favorable PK and toxicology profiles in mice, rats, and dogs, data on the normal tissue distribution and excretion of FL118 in animals would provide a better basis for explaining why FL118 possesses favorable PK and toxicology profiles, and would better inform FL118 therapeutic potential and safety profile in the clinic. Nevertheless, growing studies indicate potential advantages of using DDX5 as a cancer therapeutic target in consideration of the functions of DDX5 in normal tissues, cells, and organs versus cancer. We summarize these relevant studies in a new section below.

## DDX5’s function in normal tissue/cell/organ versus cancer

### DDX5’s inhibitory role in smooth muscle cell proliferation and tissue remodeling

An improved understanding of the physiologic functions of DDX5 in normal tissues, cells, and organs, relative to its context-dependent oncogenic functions in cancer, is important for evaluating the therapeutic window and translational potential of DDX5-directed strategies.

**Fig. 28:**
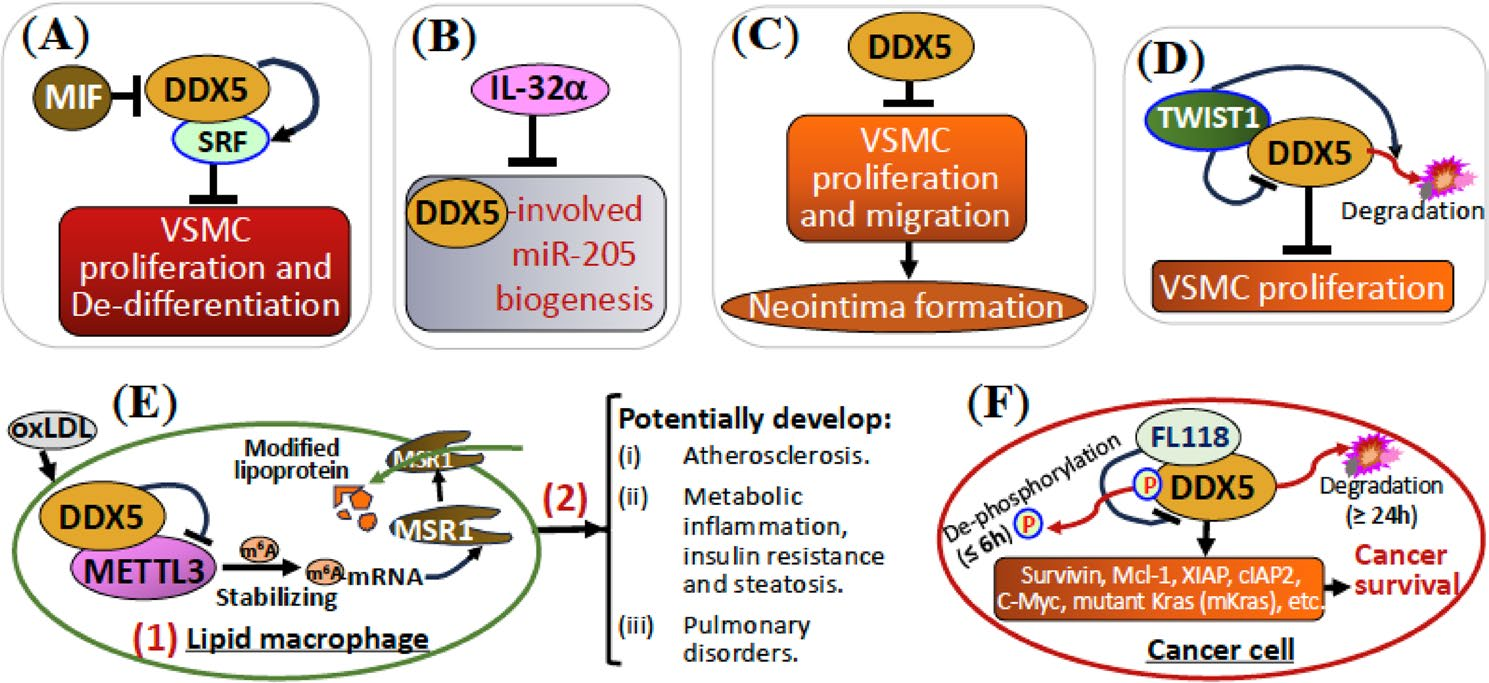
Distinct pathophysiologic functions of DDX5 in normal tissues/cells versus cancer: (**A**) DDX5 blocks vascular smooth muscle cell (VSMC) proliferation and dedifferentiation. **B** IL-32α inhibits VSMC proliferation through suppression of DDX5-involved miR-205 biogenesis. **C** DDX5 inhibits VSMC proliferation, migration, and neointimal hyperplasia. **D** TWIST1 binds to, inhibits, and induces degradation of DDX5, thereby promoting VSMC proliferation. **E** Mechanism of DDX5 involvement in promoting lipid/modified lipoprotein uptake in macrophages. **F** Mechanism of FL118 targeting DDX5 for cancer therapeutics

In this regard, the functional distinction of DDX5 in normal tissues, cells, and organs versus cancer is intriguing and may provide important insights for cancer drug development and therapeutics targeting DDX5. Several recent publications are summarized here. Fan et al. reported that DDX5 is negatively involved in macrophage migration inhibitory factor (MIF)-promoted dedifferentiation of vascular smooth muscle cells (VSMCs) [284]. Mechanistically, DDX5 interacts with and promotes serum response factor (SRF) activity to block VSMC dedifferentiation, whereas MIF suppresses DDX5 and thereby triggers VSMC proliferation and dedifferentiation. Consistently, anti-MIF treatment reversed this process, and siRNA-mediated silencing of DDX5 facilitated the dedifferentiated state of VSMCs, whereas DDX5 overexpression (OE) blocked the MIF-elicited transition [284] (Fig. 28A), suggesting that DDX5 plays an inhibitory role in normal VSMC proliferation and dedifferentiation.

Son et al. found that IL-32α exerts anti-VSMC proliferative effects through inhibition of DDX5-involved miR-205 biogenesis [285] (Fig. 28B). Fan et al. further demonstrated that DDX5 plays an inhibitory role in VSMC proliferation, migration, and neointimal hyperplasia [54] (Fig. 28C). DDX5 knockdown (KD) or knockout (KO) increased VSMC proliferation and migration, whereas DDX5 OE suppressed these processes [54]. Consistently, Zhang et al. reported that TWIST1, a basic helix-loop-helix protein, is upregulated in blood vessels in a neointimal injury model and in dedifferentiated VSMCs [286]. TWIST1 OE inhibited DDX5/p68 and promoted a proliferative VSMC phenotype, whereas siRNA-mediated silencing of TWIST1 blocked these effects [286]. Mechanistically, TWIST1 binds to DDX5 and induces its degradation (Fig. 28D), and importantly, DDX5 KD abolished the effects of TWIST1 depletion [286]. Together, these observations indicate that DDX5 plays an inhibitory role in VSMC proliferation, remodeling, and dedifferentiation, and that TWIST1 promotes VSMC remodeling at least in part by directly binding to and inducing degradation of DDX5/p68 (Fig. 28D).

In summary, unlike its well-established roles in promoting cancer cell proliferation, tumor progression, and metastasis [1, 108, 129, 130], DDX5 appears to play inhibitory roles in VSMC proliferation, tissue remodeling, and dedifferentiation (Fig. 28A-D).

### DDX5’s role in macrophage-mediated lipid metabolism

Additionally, Zhao et al. reported that DDX5 promotes oxidized low-density lipoprotein (oxLDL) uptake in oxLDL-treated human macrophages [287]. Mechanistically, oxLDL induces DDX5 expression and promotes the interaction of DDX5 with methyltransferase-like 3 (METTL3), the catalytic subunit of the N6-methyladenosine (m6A) methyltransferase complex. DDX5, in turn, inhibits METTL3-mediated m6A modification of macrophage scavenger receptor 1 (MSR1) mRNA, thereby stabilizing MSR1 mRNA and increasing MSR1 expression [287] (Fig. 28E1). MSR1 is a key receptor responsible for the uptake of modified lipoproteins, including oxLDL. This pathologic process may increase the risk of multiple human diseases: (i) in atherosclerosis, excessive macrophage uptake of oxLDL promotes foam cell formation, a central pathogenic event [288]; (ii) in obesity and fatty liver disease, chronic overnutrition or lipotoxic stress can drive the formation of lipid-rich macrophages, which are associated with metabolic inflammation, worsening insulin resistance, and progression of steatosis/steatohepatitis [289]; and (iii) in the lung, dysregulated lipid uptake and abnormal lipid accumulation can generate lipid-laden macrophages observed in several pulmonary disorders and may reflect impaired surfactant processing and inflammatory injury [290] (Fig. 28E2).

### DDX5’s role in human cancer

Consistent with the role of DDX5 in cancer pathology, Mazurek et al. showed that inhibition of DDX5 induces apoptosis in acute myeloid leukemia (AML) cells without detectable toxicity to bone marrow cells [291]. Even approximately 90% DDX5 knockdown (KD) in bone marrow did not induce detectable apoptosis or impair bone marrow function [291], suggesting that DDX5 may be largely dispensable for normal hematopoiesis and tissue homeostasis under the conditions examined. Consistently, bispecific antisense oligonucleotide (ASO)-mediated knockdown of human and mouse DDX5 by daily intraperitoneal injection for 5 weeks significantly delayed tumor growth in human castration-resistant prostate cancer (CRPC) xenograft models without detectable toxicity in mice [21]. Furthermore, toxicology studies of the DDX5 degrader FL118 demonstrated favorable toxicology profiles in rats and dogs [236].

Additionally, studies by Le et al. revealed that high DDX5 expression is strongly associated with CRPC development and shorter recurrence-free survival, and that ASO-mediated inhibition of DDX5 significantly decreased CRPC cell viability and delayed CRPC tumor growth [21]. Ling et al. further showed that the small molecule FL118 binds to, dephosphorylates, and subsequently degrades DDX5 with high specificity (without binding other tested DDX family proteins) [41] (Fig. 13G), and that DDX5 functions as a master regulator promoting the expression of multiple oncogenic proteins, including survivin, Mcl-1, XIAP, cIAP2, c-Myc, and mutant KRAS [41] (Figs. 15 and 28F). Consistent with these findings, FL118 exhibits high efficacy against DDX5-expressing pancreatic tumors [41] and multiple other difficult-to-treat cancers (Figs. 16, 18, 19, 22, 23 and 24). Supporting this concept, a recent study by Takeda et al. identified DDX5 as a critical therapeutic target in chronic myeloid leukemia (CML) and showed that FL118 is effective against BCR-ABL inhibitor-resistant CML through targeting DDX5 [234].

Taken together, the currently available evidence suggests that although DDX5 also participates in normal physiologic processes, its cancer-associated functions and dependencies support continued evaluation of DDX5 as a therapeutically actionable target.

## FL118 is a novel drug platform

### FL118-derived analogs exhibit high efficacy against rare and difficult-to-treat cancers

**Fig. 29:**
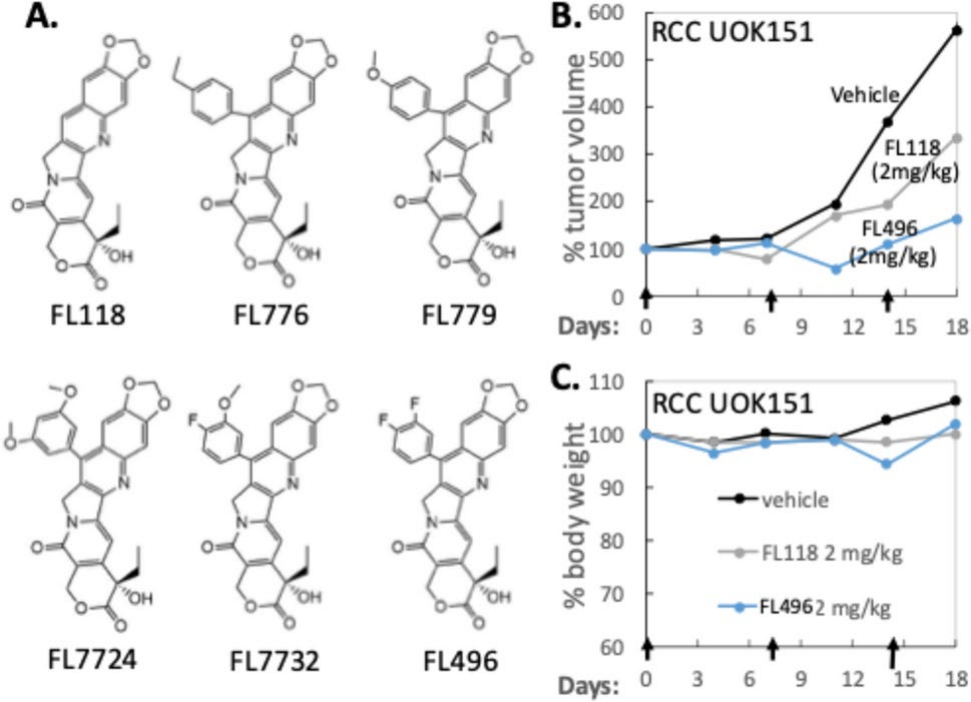
FL496 exhibits superior antitumor activity against renal cell carcinoma (RCC) compared with FL118: (**A**) Chemical structures of FL118 and identified lead FL118 analogs. **B** Comparison of FL496 and FL118 efficacy in RCC. Sub-confluent UOK151 RCC cells were harvested and resuspended in PBS containing 50% Matrigel to 1 × 10⁷ cells/mL. A total of 100 µL was injected subcutaneously per site into SCID mice. When tumors reached 800–1000 mm³, experimental models were established by implanting 35–50 mg of freshly isolated tumor tissue into the flanks of SCID mice. Once tumors reached 100–200 mm³ (day 0), mice were orally treated with vehicle, FL496, or FL118 (2 mg/kg) on days 0, 7, and 14 (arrows). For reference, FL118 MTD is ~ 10 mg/kg on a weekly ×4 schedule. Tumor growth curves are shown (variation within 10%). **C** Average percentage change in mouse body weight during treatment (variation within 8%). This figure is adapted from our previous publication [282] with additional new data

FL118 is a small molecule (MW: 392) without side-chain chemical groups (Fig. 29A). The potent antitumor activity of FL118 against human CRC and PDAC tumors in animal models [41, 46] prompted us to use the FL118 chemical structure as a platform for generating novel FL118 analogs. In our first key study, we synthesized 48 new FL118 analogs targeting either position 7 or position 9 of the molecule [292]. Following subsequent in vitro and in vivo characterization - including antitumor efficacy and toxicity assessments in animal models bearing CRC patient-derived xenograft (PDX) tumors - we identified three lead analogs (FL776, FL779, and FL7724; Fig. 29A) that share a mechanism of action (MOA) with FL118 but display enhanced antitumor activity [292].

Because all three lead compounds originated from FL118 position 7, we further synthesized a series of fluoroaryl-substituted derivatives at this position. Through a series of in vitro and in vivo studies, we identified another lead compound (7 h/FL7732; Fig. 29A) [293]. Collectively, these novel analogs expand the FL118 drug pipeline and provide promising backup or second-generation candidates for further preclinical and clinical development.

The most promising second-generation candidate is FL496 (Fig. 29A), which we first reported in 2021 [282]. Nuclear factor erythroid 2-related factor 2 (NRF2) is a key regulator of the antioxidant response element (ARE) pathway. In type 2 papillary renal cell carcinoma (pRCC) with fumarate hydratase (FH) mutation (FHpRCC), tricarboxylic acid (TCA; Krebs) cycle deficiency leads to excessive production of reactive oxygen species (ROS). This ROS accumulation activates NRF2, enhancing antioxidant defenses and protecting RCC cells from ROS-induced cell death [282].

**Fig. 30:**
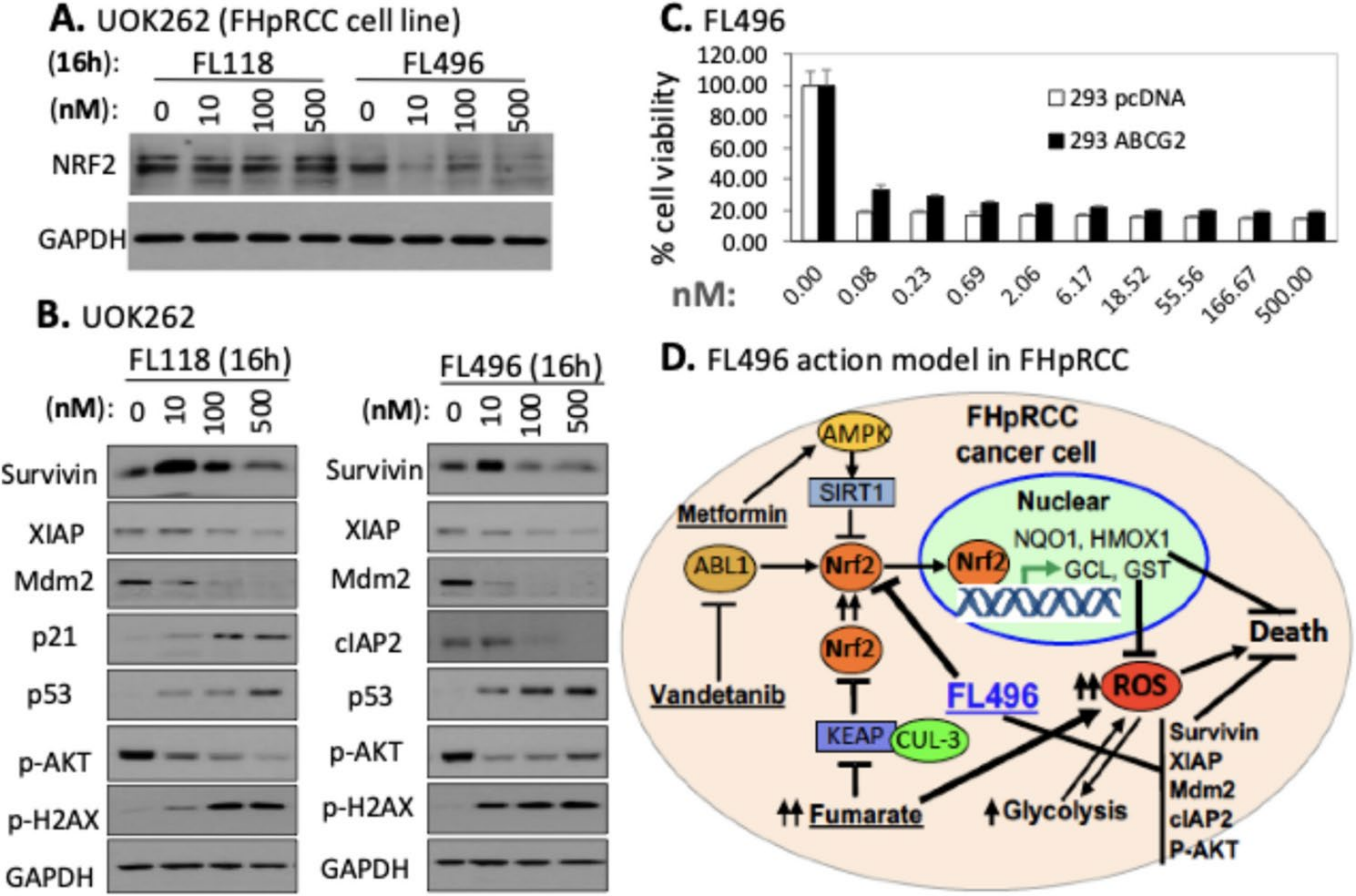
FL496, a FL118 analog, exhibits extended target specificity: (**A**) Effects of FL118 and FL496 on NRF2 expression in type-2-FH–deficient pRCC (FHpRCC) UOK262 cells. Sub-confluent cells in 6-well plates were treated for 16 h with FL118 or FL496 at 0, 10, 100, and 500 nM, as indicated, and analyzed by WB for NRF2; GAPDH served as a loading control. **B** Effects of FL118 and FL496 on potential molecular targets in UOK262 cells. Treatment was as in (A), and lysates were analyzed by WB with antibodies against the indicated proteins; GAPDH was the loading control. **C** FL496 is not an ABCG2 efflux pump substrate. HEK293 cells stably transfected with pcDNA3 (control) or pcDNA3-ABCG2 were treated with FL496 at indicated concentrations for 72 h. Viability was assessed by MTT. Each bar represents mean + SD from 5 independent experiments. **D** Proposed MOA model for FL496 based on data in (**A**, **B**), adapted from “Targeting ABL1-Mediated Oxidative Stress Adaptation” [294]. The figure format is adapted from our previous publication [282]

We found that the FL118 analog FL496 exhibits significantly stronger anti-RCC tumor activity than FL118 (Fig. 29B). Consistent with this differential efficacy, FL496 inhibited NRF2 expression in highly aggressive type 2 pRCC UOK262 cells, whereas FL118 did not (Fig. 30A). Despite this distinction, both FL118 and FL496 share largely overlapping molecular targets in UOK262 cells (Fig. 30B). Furthermore, our data indicate that FL496 is not an ABCG2 substrate (Fig. 30C), consistent with previous findings for FL118 [282].

Previous studies demonstrated that the proto-oncogene ABL1 can activate NRF2 and that the ABL1 inhibitor vandetanib exhibits efficacy in UOK262 xenograft models [294]. Based on the molecular targeting profile of FL496 and the pathways driving FHpRCC tumors, we proposed a working MOA model for FL496 (Fig. 30D). Given FL496’s inhibitory effects in FHpRCC cell and tumor models, further investigation of FL496 as a potential therapy for this rare RCC subtype is warranted.

The remarkable anti-FHpRCC activity of FL496 led us to hypothesize that FL496 may also be highly effective against other rare human cancers, including mesothelioma. Over the past three years, we have used mesothelioma as a research model to investigate FL496. Studies from our October 2025–published paper demonstrated that FL496 exhibits significantly greater anti-mesothelioma activity than FL118 [295].

**Fig. 31:**
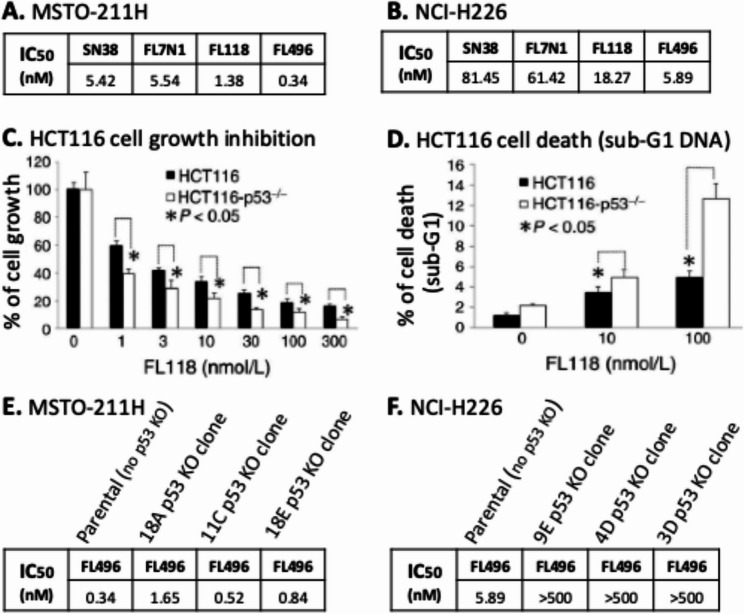
FL496 is more effective than FL118 in inhibiting mesothelioma cell growth and shows opposing p53-dependence in sensitivity relative to FL118: (**A**, **B**) IC₅₀ comparisons in two mesothelioma cell lines (MSTO-211 H, NCI-H226). Cells were seeded at 4 × 10³ cells/well in 96-well plates. After overnight incubation, cells were treated with a concentration range (0–250 nM) of SN38, FL7N1, FL118, and FL496 for 72 h. Viability was measured using Resazurin viability (MTT) assay, and IC₅₀ values were calculated. **C** Cell growth assay after 72 h FL118 treatment in HCT116 versus HCT116 p53^−/−^ cells. **D** Flow-cytometric analysis of sub-G1 DNA (dead cells) after 72 h treatment with 10 nM or 100 nM FL118 in indicated cells. **E**, **F** IC₅₀ comparisons between parental mesothelioma cells and p53 KO clones. Parental MSTO-211 H and NCI-H226 cells versus p53 KO MSTO-211 H clones (18 A, 11 C, 18E) and p53 KO NCI-H226 clones (9E, 4D, 3D) were seeded at 4 × 10³ cells/well and treated with FL496 (0, 0.08, 0.23, 0.69, 2.1, 6.17, 18.52, 55.56, 166.67, 500 nM) for 72 h. Viability was measured by MTT, and IC₅₀ values were calculated. This figure is adapted from our previous publication [296] and from the Oct 2025-pubnlished JECCR research paper [295]

FL496 exhibits markedly superior anti-mesothelioma activity compared with FL118 (Fig. 31A, B). A key mechanistic difference between FL496 and FL118 is that FL118 displays a significantly greater ability to inhibit growth/viability of CRC p53-null HCT116 cells (Fig. 31C) and to increase sub-G1 DNA content indicative of cell death (Fig. 31D). In contrast, our October 2025–published study revealed that knockout (KO) of wild-type p53 in mesothelioma cells significantly increases resistance to FL496 treatment [295]. Specifically, MSTO-211 H cells exhibited an IC₅₀ shift from 0.34 nM to 1.01 nM (Fig. 31E), while NCI-H226 cells showed an IC₅₀ shift from 5.89 nM to > 500 nM following wild-type p53 KO (Fig. 31F).

Together, these findings suggest that FL496 and FL118 may have complementary efficacy profiles against treatment-resistant cancers: tumors resistant to FL118 may remain sensitive to FL496, and vice versa, representing a highly intriguing therapeutic possibility that warrants further investigation.

Nevertheless, FL118 and its analogs are typical small molecules. However, unlike CPT, irinotecan, SN-38, and topotecan, which primarily target Top1, FL118 has a distinct mechanism of action and directly targets DDX5 and UbE2T. Because FL118 is water-insoluble, specialized formulation approaches are required to achieve optimized drug product performance. Over the past decade, multiple formulations have been developed and evaluated, culminating in a clinically compatible oral powder formulation based on an FL118–HPβCD (2-hydroxypropyl-β-cyclodextrin) complex [236].

### The methylenedioxy moiety of FL118 and its analogs is required for high antitumor activity

Emerging structure-activity relationship studies further indicate that the methylenedioxy moiety of FL118 (Fig. 29A) is critical for maintaining high anticancer activity. Substitution of this structural feature markedly reduces potency, supporting the conclusion that the methylenedioxy group is an important determinant of the therapeutic activity of FL118-derived compounds.

**Fig. 32:**
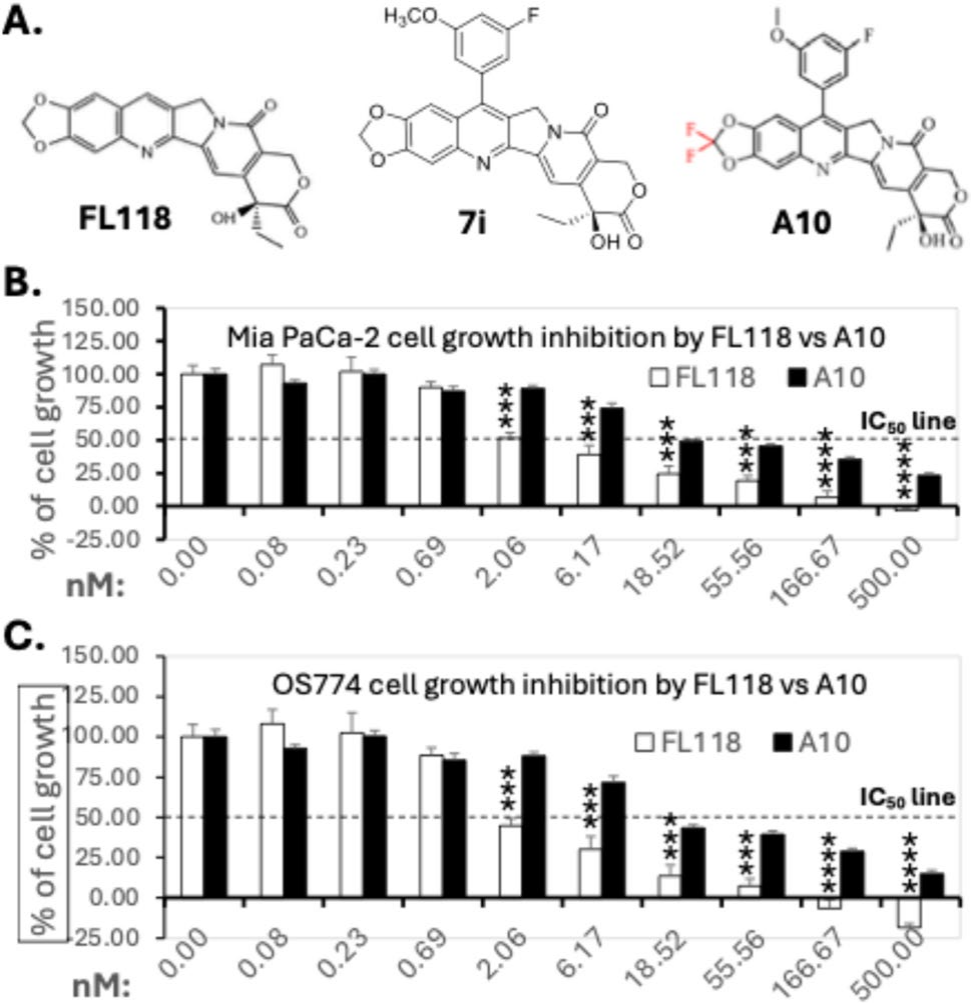
The methylenedioxy moiety of FL118 is important for maintaining high cancer cell growth-inhibitory activity: (**A**) Chemical structures of FL118, 7i, and A10. **B** Inhibition of PDAC Mia PaCa-2 cell growth by FL118 and A10. Subconfluent Mia PaCa-2 cells were treated with a series of concentrations of either FL118 or A10 as indicated. Cell growth inhibition was determined by MTT assay 72 h after treatment. Each bar represents the mean + SD from 3 independent assays. **C** Inhibition of osteosarcoma PDX-derived OS774 cell growth by FL118 and A10. Subconfluent OS774 cells were treated with a series of concentrations of either FL118 or A10 as indicated. Cell growth inhibition was determined by MTT assay 72 h after treatment. Each bar represents the mean + SD from 3 independent assays. ***p < 0.001; ****p < 0.0001

A recent study reported a new FL118-based compound, A10 (Fig. 32A), which was generated by first replacing the two hydrogen atoms of the FL118 methylenedioxy moiety with two fluorine atoms. This medicinal chemistry-modified compound, termed 2 F-FL118/2F-MPQA, was then used as a platform for three series of additional substitutions at position 7, including aryl [19], alkyl [10], and haloalkyl/halobenzyl [29] residues [297]. The structure of A10 is identical to that of 7i except for substitution of the two hydrogen atoms in the FL118 methylenedioxy moiety with two fluorine atoms (Fig. 32A). Interestingly, the IC50 of 7i in A549 lung cancer cells was previously reported as 15.5 ± 2.2 nM [293], whereas the IC50 of A10 in A549 cells was 110.8 ± 4.4 nM [297], representing an approximately 7-fold increase in IC50 following this substitution. This observation prompted us to further compare the cancer cell growth-inhibitory activities of FL118 and A10.

Using PDAC Mia PaCa-2 cells and osteosarcoma PDX-derived OS774 cells, we performed a head-to-head comparison of FL118 and A10 for inhibition of cancer cell growth and IC50 determination. Our studies showed that FL118-inhibited cancer cell growth is approximately 8- to 9-fold more potent than A10 (Fig. 32B, C). Specifically, the IC_50_ of FL118 in Mia PaCa-2 cells was 2.19 ± 0.23 nM, whereas the IC_50_ of A10 in Mia PaCa-2 cells was 17.83 ± 1.12 nM (Fig. 32B). In OS774 cells, the IC_50_ of FL118 was 1.46 ± 0.12 nM, whereas the IC_50_ of A10 was 13.36 ± 1.31 nM (Fig. 32C). Of note, the IC_50_ of A10 in Mia PaCa-2 cells reported in the recent study was 28.6 ± 1.4 nM [297], which is close to the IC_50_ of A10 determined here in Mia PaCa-2 cells (17.83 ± 1.12 nM, Fig. 32B).

Collectively, these findings further support the conclusion that FL118 is a novel drug platform and that the methylenedioxy moiety is a key structural feature required for maintaining the high anticancer activity of FL118 and its derived analogs.

## Summary

Based on our review and the collective integration of new and previously published data, it is evident that: (i) DDX5 and UbE2T physically and functionally participate in multiple cancer-related DNA repair pathways and their network crosstalk (Figs. 1, 2, 3 and 4); (ii) DDX5 physically interacts with and functionally affects multiple topoisomerases, including Top1 (Fig. 5), Top2A/Top2α, Top2B/Top2β, TopBP1 (Fig. 6), and Top3B/Top3β (Fig. 7); (iii) through direct or indirect mechanisms, DDX5 regulates and is reciprocally regulated by the EZH2 and NANOG networks, broadly influencing PCSC-driven aggressiveness in NEPC, CRPC, and mCRPC (Figs. 8, 9, 10 and 11); (iv) by interacting with and regulating multiple immune-regulatory molecules, DDX5 may play critical roles in inflammatory and immune suppression within the TME (Figs. 12A-J). Although UbE2T has limited direct involvement in cancer immune regulation (Fig. 12K), its interaction network with DDX5 in DNA repair and pathway crosstalk (Figs. 1, 2, 3 and 4) may confer both direct and indirect effects on immune modulation; (v) FL118 is a small-molecule dual molecular glue degrader that binds DDX5 and UbE2T with high specificity and selectivity (Fig. 13), resulting in reduced or abrogated UbE2T and DDX5 expression in prostate cancer (Fig. 14). Genetic modulation of DDX5 or UbE2T, similar to FL118 treatment, alters the expression of their downstream target proteins (Fig. 15), and FL118 demonstrates strong therapeutic potential against advanced prostate cancer (Fig. 16), consistent with clinical relevance of DDX5 and UbE2T high expression in advanced prostate cancer tissues (Fig. 17); (vi) consistent with its dual targeting of DDX5 and UbE2T, FL118 demonstrates high efficacy against CRC (Fig. 18), PDAC (Figs. 19 and 20), osteosarcoma and Ewing sarcoma (Figs. 21, 22 and 23), and ovarian cancer (Fig. 24). Moreover, FL118 shows potential for treating glioma and glioblastoma (Fig. 25), particularly in light of recent findings that DDX5 prevents mitochondrial ROS-mediated ferroptosis in glioma stem cells and that FL118-mediated DDX5 targeting induces glioma stem cell ferroptosis [5]; (vii) cancer cells, including cancer stem cells, expressing ABCG2 (BCRP), ABCB1 (P-gp/MDR1), and/or ABCC1 (MRP1) exhibit resistance to common anticancer agents but not to FL118 (Fig. 26; Table 1). (viii) FL118 exhibits highly favorable pharmacokinetic profiles, characterized by accumulation in human tumors and rapid clearance from the bloodstream (Fig. 27; Tables 2 and 3); (ix) the distinct functions of DDX5 in normal tissues, cells, and organs versus cancer (Fig. 28); and (x) FL118 serves as a drug platform enabling the development of several novel FL118 analogs and derivatives with promising therapeutic potential (Figs. 29, 30 and 31); its methylenedioxy moiety structure is required for high efficacy against cancer (Fig. 32).

## Conclusion

The collective comprehensive evidence assembled in this review supports the following conclusions: DDX5 and UbE2T participate in multiple cancer-associated DNA repair pathways and pathway crosstalk; DDX5 functionally interfaces with several topoisomerase-related networks; DDX5 contributes to aggressive prostate cancer programs linked to EZH2 and NANOG; DDX5 also influences inflammatory and immune-regulatory pathways in the TME; FL118 is a selective dual molecular glue degrader targeting DDX5 and UbE2T; FL118 exhibits strong activity across multiple difficult-to-treat cancers including rare cancers; cancer cells resistant to common anticancer agents through ABC transporter expression remain sensitive to FL118; FL118 shows favorable pharmacokinetic and toxicology profiles; the distinct roles of DDX5 in normal tissues versus cancer further support its cancer therapeutic relevance; and FL118 serves as a drug platform for generating novel analogs, with the methylenedioxy moiety representing an important structural feature for high anticancer activity.

## Supplementary Information

Supplementary Material 1.

## Data Availability

No datasets were generated or analysed during the current study.

## Abbreviations

ABCG2: ATP-binding cassette sub-family G member 2
alt-NHEJ/alt-EJ/MMEJ: Alternative NHEJ/alternative end joining/microhomology-mediated end joining
BER: Base excision repair
BCRP: Breast cancer resistance protein
ChIP: Chromatin immunoprecipitation
c-NHEJ: Classical NHEJ
CRC: Colorectal cancer
CRPC: Castration-resistant PCa
CSC: Cancer stem cell
DDR: DNA damage response
DDX5: DEAD box RNA helicase 5
DNA-PKcs: DNA-dependent protein kinase catalytic subunit
DSB: Double strand break
EMT: Epithelial mesenchymal transition
ESCs: Embryonic stem cells
FA: Fanconi Anemia
GBM: Glioblastoma multiforme
GG-NER: Global genome NER
GSC: Glioma stem cell
HR: Homologous recombination
HRR: HR repair
ICLs: Interstrand crosslinks
IP: Immunoprecipitation
KD: Knockdown
KO: Knockout
mCRPC: Metastatic CRPC
mKras: Mutant Kras
MOA: Mechanisms of action
MTD: Maximum tolerated dose
NEPC: Neuroendocrine PCa
NER: Nucleotide excision repair
NHEJ: Non-homologous end joining
PCa: Prostate cancer
PCSCs: PCa stem cells
PDAC: Pancreatic ductal adenocarcinoma
PDX: Patient-derived xenograft
Pgp: P-glycoprotein
PK: Pharmacokinetics
PPI: Protein-protein interaction
SSBs: Single-strand breaks
ssDNA: Single strand DNA
TC-NER: Transcription-coupled NER
TF: Transcription factor
TLS: Translesion synthesis
TME: Tumor microenvironment
Top1: Topoisomerase 1
UbE2T: Ubiquitin-conjugating enzyme E2T
