# Supplementary material for: DDX5 (p68) and UbE2T as emerging superior cancer therapeutic targets: dual molecular glue target degradation by FL118 for conquering difficult-to-treat cancers
Source: J Exp Clin Cancer Res. 2026 May 18;45:156. doi: 10.1186/s13046-026-03733-3 (PMC13352852; doi:10.1186/s13046-026-03733-3)
Supplement: Supplementary file 1 — Supplementary Material 1. [file 13046_2026_3733_MOESM1_ESM.pdf]

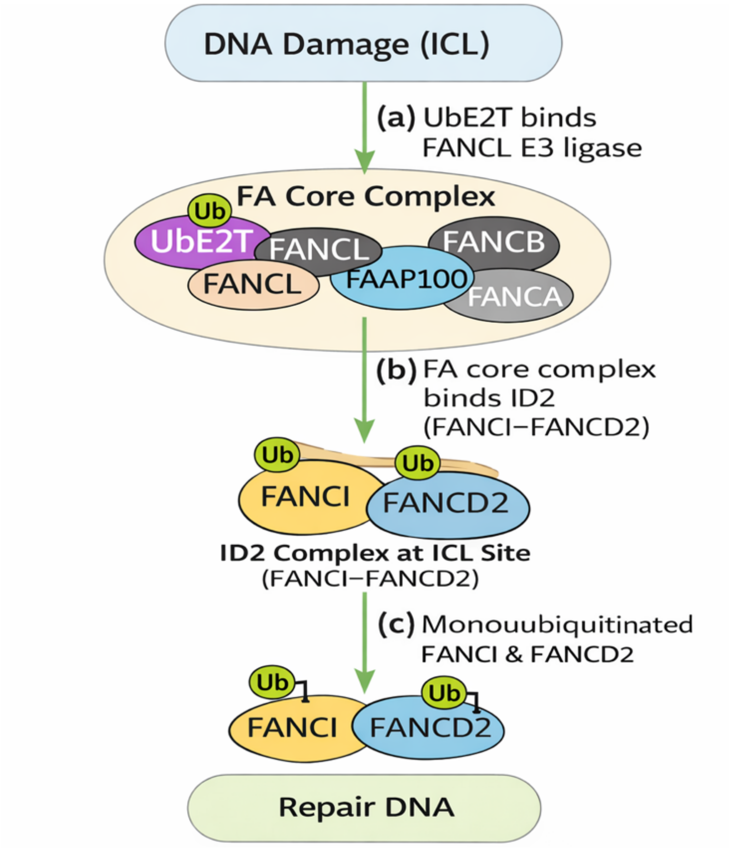

Supplementary Figure S1.

Simplified schematic of the research observation-based model for UbE2T physical and functional interactions with proteins in the FA interstrand crosslink (ICL) repair pathway:

(A) Upon DNA interstrand crosslink (ICL) damage, UbE2T binds the FANCL E3 ligase and helps assemble the FA core complex.

(B) The FA core complex is then recruited to the FANCI–FANCD2 (ID2) complex at the ICL site through UbE2T-associated interactions.

(C) In coordination with FANCL, UbE2T promotes monoubiquitination of FANCI and FANCD2, resulting in activation of the ID2 complex.

(D) The monoubiquitinated ID2 complex subsequently recruits downstream DNA repair factors, including BRCA1, BRCA2, RAD51, and others, to facilitate ICL repair.

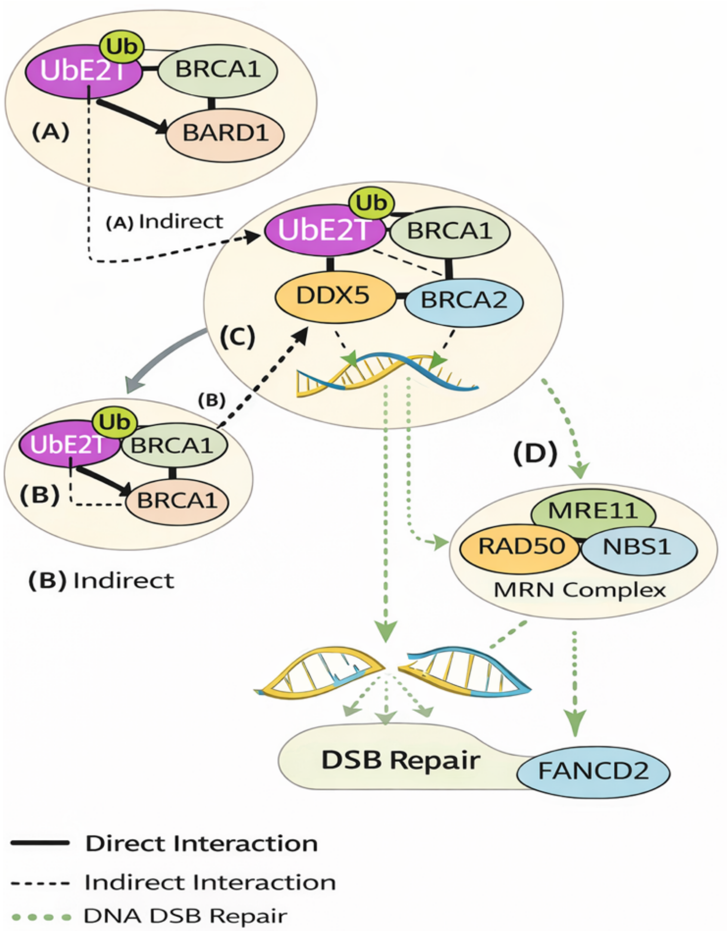

Supplementary Figure S2.

Schematic diagram of the expected UbE2T–DDX5 crosstalk through DNA repair-relevant proteins:

(A) UbE2T interacts with and colocalizes with the BRCA1–BARD1 complex.

(B) BRCA1 may provide an indirect link between UbE2T and DDX5.

(C) Available evidence supports direct and indirect interactions among UbE2T, BRCA1, DDX5, and BRCA2.

(D) The MRN (MRE11–RAD50–NBS1) complex stabilizes FANCD2 and mediates DNA double-strand break repair.

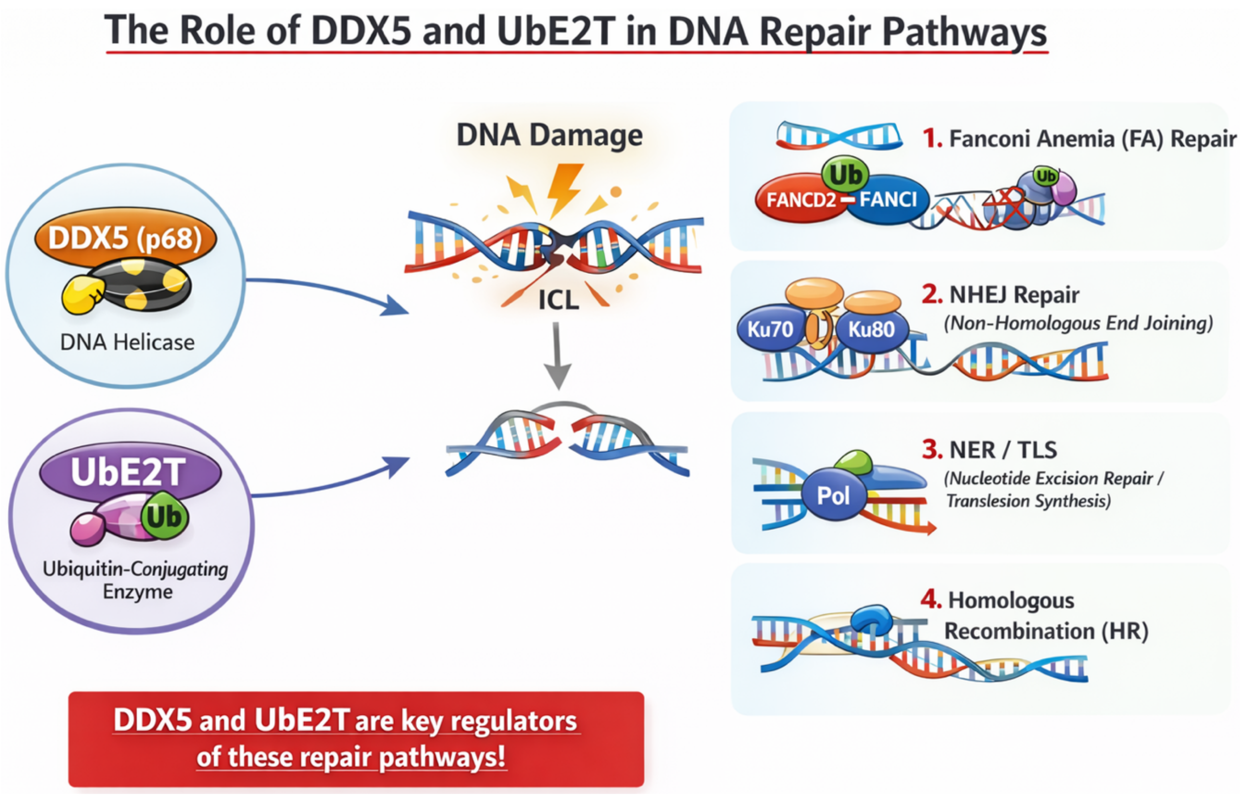

Supplementary Figure S3.

DDX5 and UbE2T help cancer cells repair damaged DNA. When DNA is damaged, cells activate several repair systems. **UbE2T** is especially important in the FA pathway through regulation of the **FANCD2-FANCI** complex, while **DDX5** may help coordinate broader repair responses. Together, these proteins may promote cancer cell survival under DNA-damaging stress. This simplified figure shows that **DDX5** and **UbE2T** may help control or support four major DNA repair pathways:

(1) Fanconi anemia (FA) repair, which is central for ICL recognition and processing

(2) Non-homologous end joining (NHEJ), which repairs DNA double-strand breaks.

(3) Nucleotide excision repair/translesion synthesis (NER/TLS), which helps remove lesions or bypass unrepaired DNA damage during replication

(4) Homologous recombination (HR), a high-fidelity pathway for repair of DNA double-strand breaks and stalled replication-associated lesions.

Overall, the figure illustrates that DDX5 and UbE2T are not isolated factors, but rather participate in a broader DNA repair network that may help cancer cells survive genotoxic stress.
